# Isochromene and Dihydroisobenzofuran Electrosynthesis by a Highly Regiodivergent Cyclization of 2‑Ethynylbenzaldehydes

**DOI:** 10.1021/acs.joc.5c00967

**Published:** 2025-06-30

**Authors:** Guilherme M. Martins, Guilherme B. Simoso, Pedro P. de Castro, Felipe de Lucca, Samuel R. Mendes, Timothy J. Brocksom, Kleber T. de Oliveira

**Affiliations:** † Department of Chemistry, Federal University of São Carlos, São Carlos, São Paulo 13565-905, Brazil; ‡ Department of Pharmacy, Federal University of Juiz de Fora—Campus Governador Valadares, Governador Valadares, Minas Gerais 35010-177, Brazil; § Department of Chemistry, State University of Santa Catarina, Joinville, Santa Catarina 89219-719, Brazil

## Abstract

The synthesis of isochromene and dihydroisobenzofuran compounds is presented through the electrochemical cyclization of 2-ethynylbenzaldehydes using silver as a sacrificial electrode. The results demonstrate exceptional regiodivergency, without the use of external oxidants or transition metal catalysts, with products reaching up to 97% isolated yield. Density functional theory (DFT) studies, along with control experiments, were conducted to elucidate a plausible reaction mechanism.

## Graphical abstract

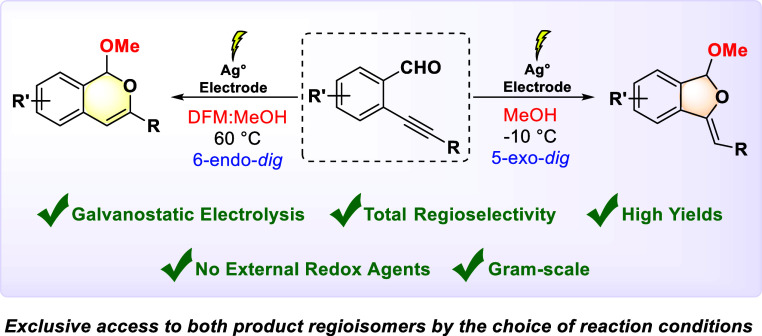

## Introduction

2-Ethynylbenzaldehydes are useful intermediates with remarkable versatility in cyclization reactions, leading to compounds with polycyclic cores, including heterocyclic derivatives such as isochromenes and dihydroisobenzofurans.^1–4^ Isochromene and dihydroisobenzofuran cores play a relevant role in medicinal chemistry due to their diverse biological activities and pharmaceutical applications and are found in many natural products.^5^ Isochromene derivatives stand out for their potential as antitumor agents, showing promising results against different types of cancer cells.^6,7^ In addition, these compounds also exhibit antifungal,^8^ antibacterial,^9^ and antimicrobial properties,^10^ highlighting their pharmacological effects and possible medicinal applications. Similarly, dihydroisobenzofuran derivatives demonstrate antiviral,^11,12^ anticancer,^13,14^ antidepressant activities,^15^ and are applied in perfume formulations.^16^ Synthetic methods explored for the synthesis of these derivatives include the use of transition metal catalysis (such as Pd,^17–22^ Ag,^23–26^ Au^27^), halocyclization^28–30^ or the use of Brønsted acids.^31–33^ The primary limitation of these protocols is that they typically yield the products as a mixture of regioisomers or require costly catalysts/ligands for the transformations. For example, the protocols presented in Scheme 1A,B.^34,35^

**Scheme 1:**
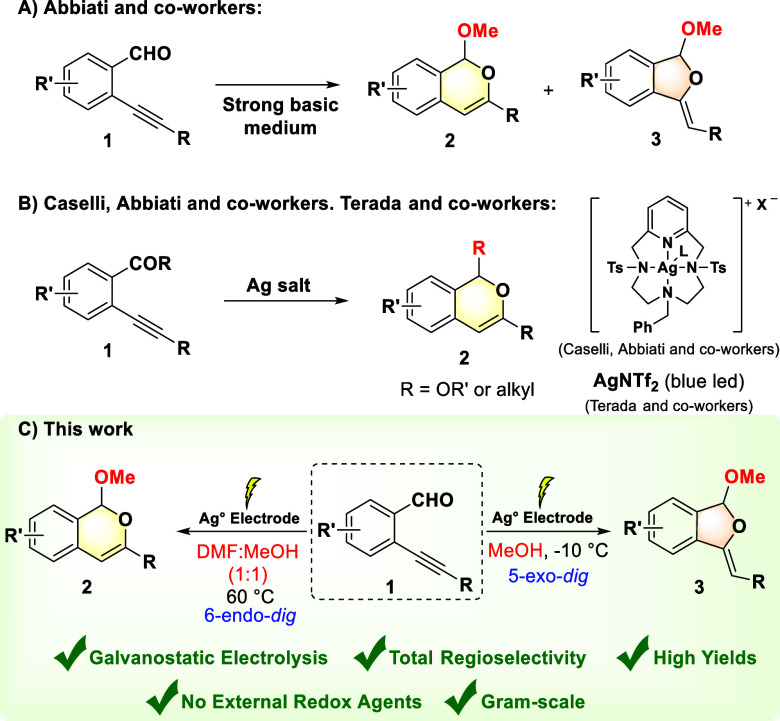
(A) Base-Promoted Synthesis of Dihydroisobenzofurans;^35^ (B) Synthesis of Isochromenes Catalyzed by Silver Salt;^23,24^ (C) Regiodivergent Electrosynthesis of Isochromene (**2**) and Dihydroisobenzofuran (**3**) Compounds

In this context, electrosynthesis offers several advantages,^36,37^ such as higher selectivities and organic transformations under milder conditions.^38–40^ In addition, electrochemical methods can be applied to a wide range of substrates and functional groups, making it a valuable tool for synthetic transformations.^41–49^ Finally, organic electrosynthesis can be scalable, making it suitable for both laboratory research and industrial applications. Moreover, methodologies such as flow chemistry can further enhance scalability, ensuring greater efficiency and reproducibility in larger-scale processes.^50–56^

The increasing interest in using sacrificial electrodes in organic electrosynthesis has become evident.^57^ These electrodes, typically made of metals with low oxidation potentials, release metal ions into the solution during electrochemical processes and can be applied as catalysts or coordinating agents in organic reactions. Metals such as magnesium, copper, zinc, aluminum, iron, and silver are often used as sacrificial electrodes in electro-organic transformations,^58–63^ but a key limitation is that the electrode becomes nonrecyclable due to the formation of metal salts. However, the benefits of this method can outweigh these drawbacks. These metals in solution can prevent the overoxidation of substrates, activate intermediates, and act as replacements for catalysts.^57^

Considering this, we propose a synthetic methodology for obtaining isochromenes and dihydroisobenzofurans through the electrosynthetic regiodivergent cyclization of 2-ethynylbenzaldehydes using a silver electrode (Scheme 1C), avoiding the formation of mixtures of regioisomers and eliminating the need for complex ligands/catalysts.

## Results and Discussion

In the initial optimization, several variables were considered, including electrolytes, solvents, electrodes, temperature, reaction time, among others (Table 1). The optimized standard conditions at 60 °C, using a mixture of MeOH/DMF (1:1) with 1 equiv of LiClO_4_ and AcOH, showed total regioselectivity in the formation of product **2a**, achieving a yield of 95% (determined by quantitative ^1^H NMR) and 93% isolated yield (entry 1). Notably, in this condition, product **3a** was not observed, and the complete consumption of starting material **1a** occurred in 3 h with a current of 6 mA. The absence of AcOH reduced the yield of product **2a** to 67%, while product **3a** was formed in 10% yield (entry 2). Without AcOH and at room temperature, we observed an increase in the formation of product **3a**. Temperature and the presence of AcOH were identified as significant factors for reaction efficiency and complete regioselectivity (entry 3). Decreasing the reaction time to 2 h resulted in 73% yield for **2a** (entry 4), while decreasing the constant current to 3 mA led to reduced yields, with 36% for **2a** (entry 5). Applying tetrabutylammonium perchlorate (TBAClO_4_) or TBABF_4_ as electrolytes instead of LiClO_4_ resulted in decreased yields for **2a**, with 72% and 21%, respectively (entries 6 and 7). The use of NaCl as an electrolyte was not effective (entry 8). Substituting DMF for toluene resulted in **2a** in 84% yield, with no formation of **3a** (entry 9). In contrast, DCM was not suitable for the transformation (entry 10). Applying sacrificial electrodes of Mg, Cu, Zn or SS did not afford products (entry 11). Using just MeOH as solvent without AcOH at room temperature, product **3a** was obtained in 82% yield and product **2a** in 15% (entry 12). Reducing the temperature to −10 °C enabled the totally regioselective preparation of product **3a** in 87% yield, whereas, under a current of 3 mA, the yield of product **3a** decreased to 48% (entries 14 and 15). The main variables influencing the regioselectivity of these products were the temperature and the absence of DMF. The total release of silver into the reaction was 2.7 equiv (see the Supporting Information for more information).

**Table 1:**
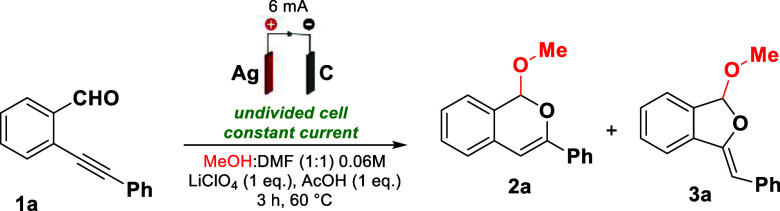
Optimization of the Reaction Conditions for the Synthesis of Isochromene and Dihydroisobenzofuran **2a** and **3a**

entry	changes from standard conditions	yield^b^	
		**2a**	**3a**
1	**none**	**95 (93)** ^c^	**0**
2	without AcOH	67	10
3	without AcOH, at rt	60	22
4	2 h instead 3 h	73	0
5	3 mA instead 6 mA	36	0
6	TBAClO_4_ instead of LiClO_4_	72	0
7	TBABF_4_ instead of LiClO_4_	21	0
8	NaCl instead of LiClO_4_	0	0
9	toluene instead DMF	84	0
10	DCM instead DMF	0	0
11	Mg, SS, Cu or Zn instead Ag	0	0
12	without DMF and AcOH, at rt	15	82
13	without DMF and AcOH, at 0 °C	5	84
14	**without DMF and AcOH, at** **–10 °C**	**0**	**87 (85)** ^c^
15	entry 14, at 3 mA	0	48

a Reaction conditions: **1a** (0.25 mmol), LiClO_4_ (0.25 mmol, 1 equiv), AcOH (0.25 mmol, 1.0 equiv) in MeOH/DMF (1:1, 0.06 M), Ag anode, C cathode, undivided cell, constant current = 6 mA, at 60 °C under air for 3.0 h.

b By ^1^H NMR using 1,3,5-trimethoxybenzene (TMB) as internal standard.

c Isolated yield.

After establishing the optimal reaction conditions, the methodology was evaluated for a representative substrate scope (Scheme 2, methods A and B). For derivatives of isochromene **2** (method A), the model compound **2a** was obtained in 95% yield. The transformation tolerated aryl groups in R^2^ bearing halides (derivatives **2b**–**2c**), affording products with isolated yields of up to 94%. Furthermore, the derivatives with *para or meta*-tolyl substituents (**2d**–**2e**) were successfully synthesized with a yield up to 96%. Aliphatic groups in R^2^ also proved fruitful, providing **2f** in 53% yield. The best yield (97%) was achieved when R^1^ was substituted with F, resulting in **2g**. Substrates with R^1^ = F and aryl groups in R^2^ containing halides exhibited excellent reactivity, with products obtained in 88–90% yields (**2h**–**2i**). Starting materials substituted with R^1^ = OMe presented a small decrease, both for R^2^ = Ph (product **2j**, 86% yield) and for R^2^ containing *para*-Cl substituent (product **2k**, 81% yield). Using 2-(phenylethynyl)­nicotinaldehyde, **2l** was obtained with an 84% yield, while using 2-(3-hydroxy-3-methylbut-1-yn-1-yl)­benzaldehyde, **2m** was obtained in 92% yield. It was envisioned that the methodology could be applied for the synthesis of 3,4-dihydro-1*H*-1,5-epoxybenzo­[*c*]­oxocine **2n**, however, even with an extended reaction time, only the starting material was recovered.

**Scheme 2:**
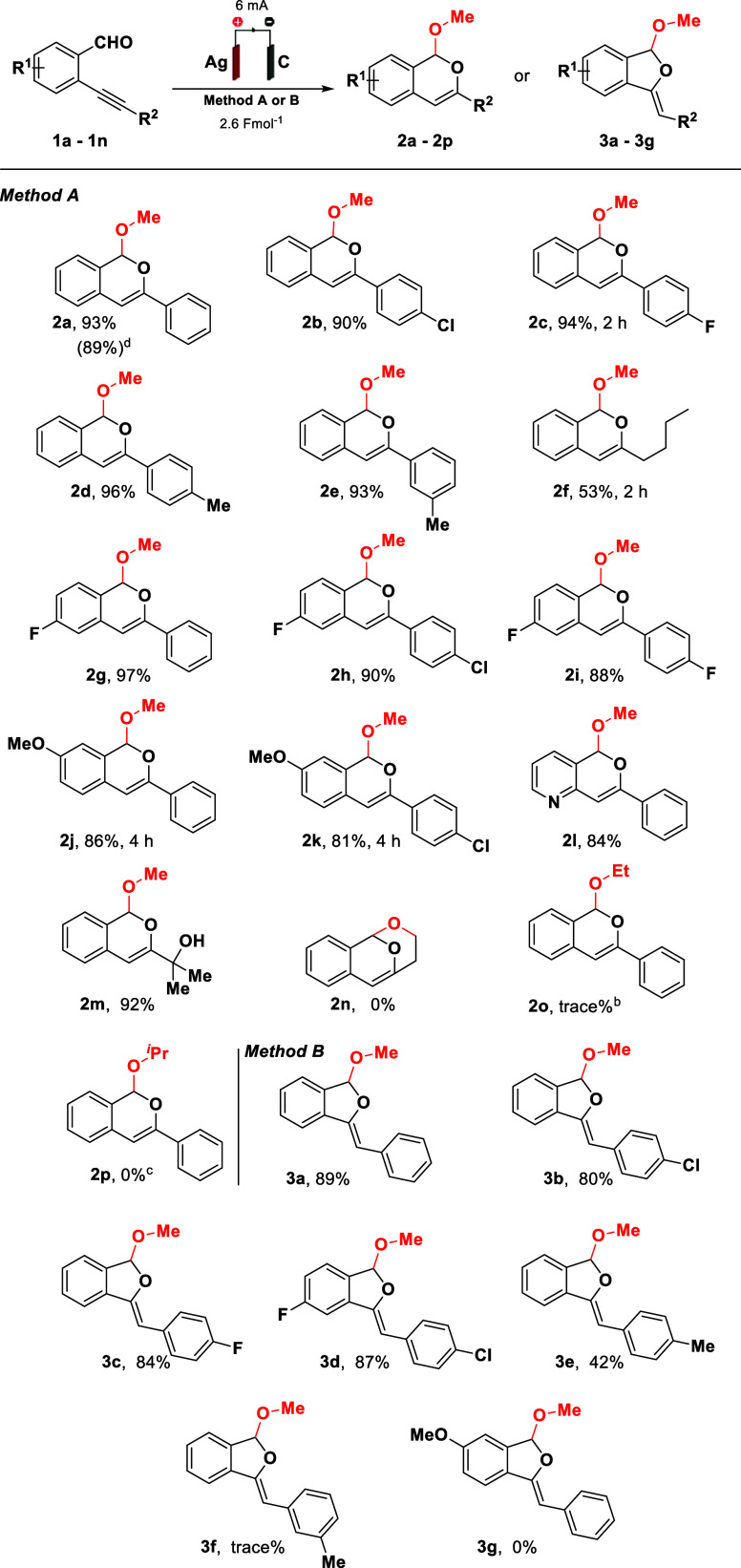
Substrate Scope for the Synthesis of Isochromene **2** and Dihydroisobenzofuran **3** Derivatives^a^

a Reaction conditions: Method A: **1** (0.25 mmol), LiClO_4_ (0.25 mmol, 1 equiv), AcOH (0.25 mmol, 1.0 equiv) in MeOH/DMF (1:1, 0.06 M), Ag anode, C cathode, undivided cell, constant current = 6 mA, at 60 °C under air for 3 h. Method B: **1** (0.25 mmol), LiClO_4_ (0.25 mmol, 1 equiv), in MeOH (0.06 M), Ag anode, C cathode, undivided cell, constant current = 6 mA, at −10 °C under air for 3 h. Isolated yield.
b Using EtOH instead of MeOH.
c Using ^*i*^PrOH instead of MeOH.
d Reaction carried out using a 5 mmol scale for 30 h at a constant current of 6 mA.

The use of different alcohols as solvents was evaluated, however, with EtOH, only traces of the product **2o** were observed by GC–MS, while with ^*i*^PrOH (**2p**), no products were observed, leaving only the starting material **1a**. Method A was scaled up to 5.0 mmol, with a reaction time of 30 h and using a constant current of 6 mA. This reaction yielded **2a** in 89% (1.059 g).

For derivatives of dihydroisobenzofuran **3** (method B), the model product **3a** was obtained in 89% yield. The reaction tolerated the incorporation of aryl groups in R^2^ bearing halides (**3b**–**3d**), affording products in up to 87% isolated yield. However, to our surprise, product **3e** (*para*-tolyl-substituent) was obtained in only 42% yield, while product **3f** (*meta*-tolyl-substituent) was formed only in trace amounts, as observed by GC–MS. The dihydroisobenzofuran **3g** was not detected, with only the starting material remaining, even at extended reaction times.

To gain insight into the reaction mechanism, a series of control experiments were performed (Scheme 3). To understand the role of the silver electrode, 1 equiv of silver salts such as AgOAc and Ag_2_O was applied using an inert graphite electrode under a current of 6 mA, however, no product was observed. This result not only highlights the relevance of Ag^+^ in the protocol but also underscores the crucial role of the anodic sacrificial electrode in protecting the reaction system, potentially preventing the degradation of isochromene or dihydroisobenzofuran products by the anode. Additionally, we evaluated the deuteration of compound **2a** using CD_3_OD. The experimental results indicated 60% deuteration, specifically at the 4-position of the isochromene ring. The anodic reaction using a divided cell (ceramic frit separator) was effective in the formation of isochromene **2a**, suggesting that acetate can also be formed during the reaction course and not only by acetic acid decomposition at the cathode. The anode was assembled with a silver electrode, while the cathode was composed of graphite.

**Scheme 3:**
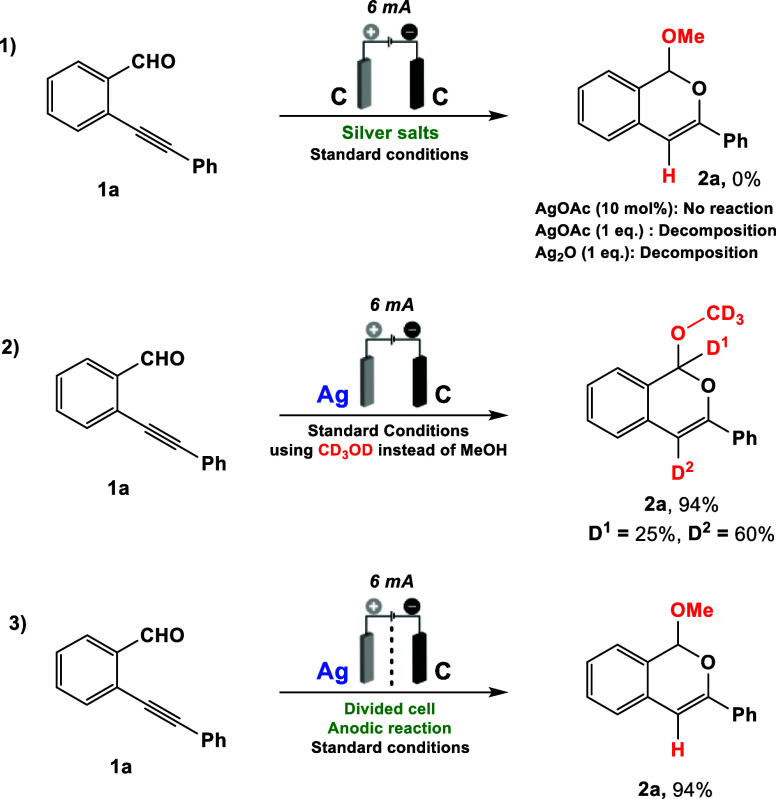
Control Experiments^a^

a Standard conditions: **1a** (0.25 mmol), LiClO_4_ (0.25 mmol, 1 equiv), AcOH (0.25 mmol, 1 equiv) in MeOH/DMF (1:1, 0.06 M), Ag anode, C cathode, undivided cell, constant current = 6 mA, at 60 °C under air for 3 h. Isolated yield.

To evaluate the dependence of the reaction on the applied current, ON/OFF experiments were conducted under standard electrochemical conditions. The transformation proceeded efficiently while the power source was switched ON. However, upon interruption of the current (OFF), the formation of product **2a** ceased immediately. When the electric current was reapplied (ON), the reaction continued, leading to the formation of product **2a**. These results demonstrate that continuous application of the electric current is essential for sustaining the transformation, highlighting the crucial role of electrochemical activation in the reaction pathway (see the Supporting Information for more information).

Considering the results obtained from the ON/OFF control experiments, in conjunction with the studies involving AgOAc and Ag_2_O-mediated transformations, we propose that the concentration and the well-controlled generation of Ag^+^ in the reaction medium influence the efficiency of the reaction. It was observed that the immediate introduction of a high concentration of Ag^+^ into the reaction medium leads to significant decomposition of the materials, which compromises the efficiency of the transformation. In contrast, the well-controlled and continuous electrochemical generation of Ag^+^ favor the reaction, while preventing substrate degradation. These findings indicate that a gradual and sustained formation of Ag^+^ is pivotal for achieving selective and efficient conversions.

The cyclic voltammetry experiment (Figure 1) showed a reductive peak in −1.3 V for **1a** and −0.5, and −0.6 mV for products **2a** and **3a**, respectively. These values suggest the possibility of parallel reactions or degradation during electrolysis. However, we believe that both MeOH and AcOH, besides playing fundamental roles in the reaction mechanism, also act in the protection of the electrolytic system, promoting cathodic processes and avoiding parallel reactions.

**Figure 1:**
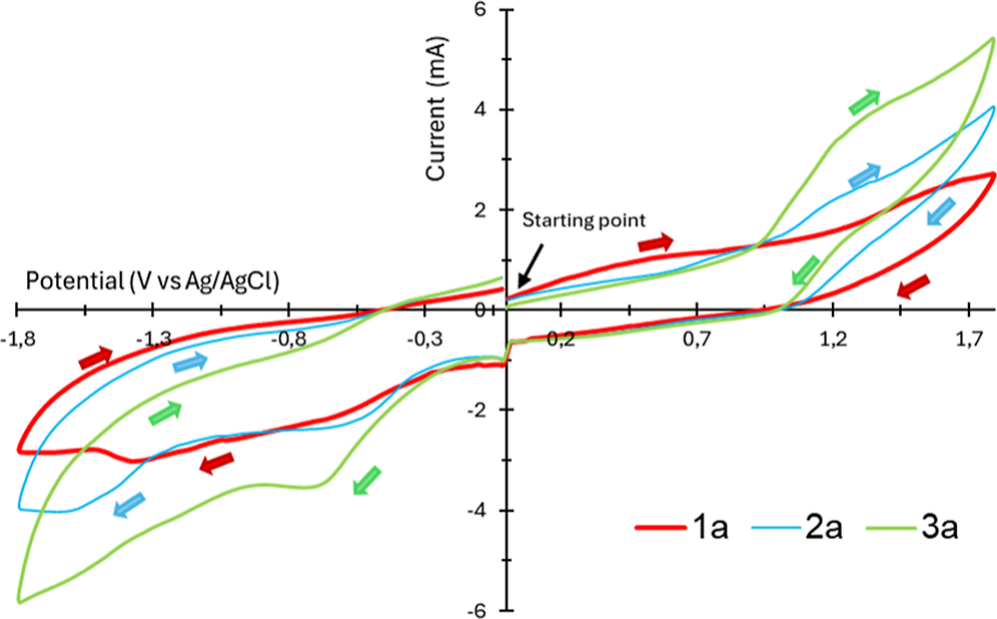
Cyclic voltammograms of **1a**, **2a** and **3a**. Working electrode: carbon electrodes; reference electrode: Ag/Ag^+^; supporting electrolyte: LiClO_4_ (0.06 mol L^–1^); scan rate: 10 mV s^–1^. Charting with IUPAC.

The reaction mechanism for the silver-catalyzed cyclization of 2-ethynylbenzaldehyde (**1**) was proposed based on the electrochemical data, DFT calculations, and previous literature reports.^64,65^ Several DFT calculations were performed to elucidate the mechanism involving isochromene formation (proposals 1–5 in the Supporting Information file). The most viable reaction pathway (Scheme 4 and proposal 4 in the Supporting Information file) initiates with the activation of the triple bond in **1** by Ag^+^ ions, facilitating the intramolecular cyclization via a 6-endo-*dig* pathway (Δ*G* ^‡^ of 17.25 kcal mol^–1^ against 20.25 kcal mol^–1^ for the 5-exo-*dig* cyclization). This is the rate-limiting step for this transformation and the energy gap between both pathways (ΔΔ*G* ^‡^ of 3.00 kcal mol^–1^) allows the kinetic controlled synthesis of the isochromene derivative. Next, the intermediate **A** undergoes a silver/hydrogen exchange mediated by AcOH, affording intermediate **B**, followed by a nucleophilic attack by MeOH to produce **C**. Subsequent deprotonation of **C** by acetate ion yields the isochromene **2**.

**Scheme 4:**
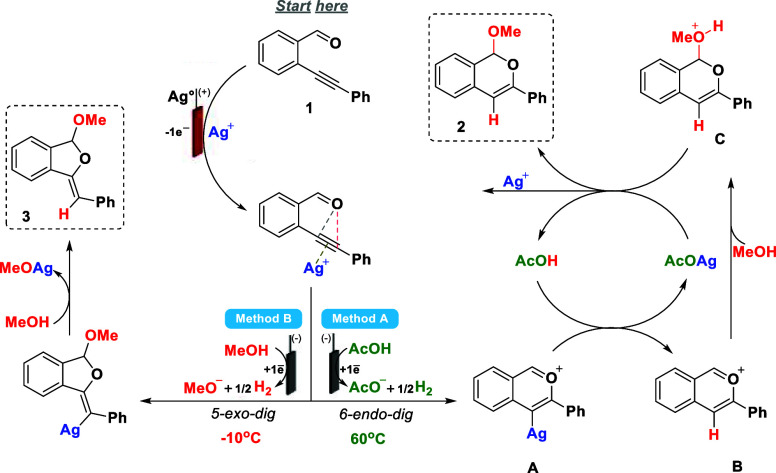
Plausible Reaction Mechanism

For the formation of dihydroisobenzofuran **3**, the reaction follows a similar reaction pathway to previous literature studies,^35^ involving the formation of the methoxide ion. In this case, the methoxide ion is formed at the cathode through the reduction of MeOH to MeO^–^ with the release of hydrogen gas. These findings are consistent with the DFT calculations that in the presence of silver methoxide the intramolecular cyclization via a 5-exo-*dig* of 2-ethynylbenzaldehyde to **3** (Δ*G* ^‡^ = 1.16 kcal mol^–1^) is more kinetically favorable than to **2** (Δ*G* ^‡^ = 5.86 kcal mol^–1^), which explains the selectivity toward the five-membered product (for full details see proposal 5 in the Supporting Information file).

## Conclusions

In conclusion, we have developed an efficient and highly regioselective electrochemical methodology for the synthesis of the isochromene or dihydroisobenzofuran moieties by the cyclization of 2-ethynbenzaldehydes. Total regioselectivity was achieved, resulting in yields of up to 97%, without requiring external oxidants or transition metal catalysts, despite, in the scope, some dihydrobenzofuran (5-member-rings) could not be obtained compared to isocromene (6-member-rings). To the best of our knowledge, this is a rare case of exclusive access to both product regioisomers by the suitable choice of electrochemical reaction conditions, such as solvent and temperature. In addition, to elucidate the reaction mechanism, several experiments were performed, including cyclic voltammetry to study the electrochemical properties of reagents, gram-scale to evaluate their viability on a larger scale, and control experiments. This combined approach to theoretical studies using density functional theory (DFT) calculations allowed a better comprehension of the reaction mechanism and the factors that influence regioselectivity.

## Experimental Section

### General Information

All purchased chemicals were used as received without further purification. Analytical TLC was performed on TLC plates (silica gel 60 F254) and visualized employing a UV lamp and/or acidic ethanolic vanillin solution (5% in 10% H_2_SO_4_) as a revelator. Yields refer to purified compounds which are spectroscopically pure. Both ^1^H and ^13^C­{^1^H} NMR spectra were recorded at 400 and 100 MHz, respectively. Chemical shifts are informed in ppm downfield from the signal of TMS, used as an internal standard, and the coupling constants (*J*) are expressed in Hertz (Hz). Chemical shifts are reported employing the following abbreviation pattern: s (singlet), d (doublet), dd (doublet of doublet), dt (doublet of triplet), t (triplet), q (quartet), and m (multiplet). Low-resolution mass spectra were obtained from a Shimadzu GC-MS-QP2020 NX mass spectrometer.

The calculations were carried out using the Gaussian 09 package (revision D01).^66^ The density functional theory (DFT) was employed in the optimization of the structures of all molecular complexes and transition states (TSs). The M06-2X functional (grid = ultrafine), the LanL2DZ basis set and the solvation model based on density (SMD) for *N*,*N*-dimethylformamide were used in the calculations.^67^ The TSs were optimized using the Berny algorithm, presented a single imaginary frequency and were fully characterized through the analysis of the intrinsic reaction coordinate (IRC).^68^ The thermodynamic properties were calculated using a temperature of 333.15 K (60 °C) (proposals 1–4, Supporting Information file) or 263.15 K (−10 °C) (proposal 5, Supporting Information file) and a pressure of 1 atm, aiming to reproduce the experimental conditions. The vibrational analysis of each species was carried out to confirm the identity of all stationary points and in the determination of the thermal corrections to enthalpy and Gibbs free energy.

Substituted 2-ethynylbenzaldehydes were prepared following literature protocols.^4,23,69^ The electrochemical reactions were carried out using a power supply (AFR—model FA3005P).

### General Procedure for the Synthesis of **2a–2m**

The electrochemical reactions were carried out in an undivided cell of 10 mL (glass bottle with plastic screw cap) equipped with a silver anode (99.9%—55 mm × 5 mm) and a carbon cathode (51 mm × 8 mm). Inside the electrochemical cell, the substrate (0.25 mmol), dimethylformamide (DMF) (2 mL), methanol (MeOH) (2 mL), lithium perchlorate (LiClO_4_) (0.25 mmol—27 mg), and acetic acid (AcOH) (0.25 mmol—14 μL) were added. A constant current of 6.0 mA at 60 °C was used for 3 h. After the reaction, the reaction mixture was filtered through a Büchner funnel with a porous plate (50 mL) with approximately half of the funnel containing common silica (70–230 mesh ASTM) in ethyl acetate (100 mL). The ethyl acetate solution was extracted with H_2_O (35 mL × 3) and dried over Na_2_SO_4_. After removal of the solvent under reduced pressure, some compounds were recrystallized or purified by chromatography on silica gel flash (elution: toluene). The products were dried under a high vacuum and the necessary analyses were performed.

#### 1-Methoxy-3-phenyl-1*H*-isochromene (**2a**)^70^

White solid, (56.5 mg, 0.24 mmol, 95% yield), mp 51.1–52.2 °C. The reaction was purified through recrystallization using petroleum ether. ^1^H NMR (400 MHz, CDCl_3_): δ 7.75 (d, *J* = 7.4 Hz, 2H), 7.36–7.26 (m, 4H), 7.23–7.15 (m, 3H), 6.55 (s, 1H), 6.08 (s, 1H), 3.54 (s, 3H). ^13^C­{^1^H} NMR (100 MHz, CDCl_3_): δ 148.4, 133.4, 129.2, 128.5, 127.8, 127.5, 126.0, 125.7, 124.8, 123.8, 123.5, 99.4, 98.7, 54.2. GC–MS (*m*/*z*, rel. int. %): 238 (M^+^, 24), 207 (100), 178 (40), 165 (10), 105 (14), 77 (22).

#### 3-(4-Chlorophenyl)-1-methoxy-1*H*-isochromene (**2b**)^71^

White solid, (61.2 mg, 0.22 mmol, 90% yield), mp 108.2–109.2 °C. The reaction was purified through recrystallization using petroleum ether. ^1^H NMR (400 MHz, CDCl_3_): δ 7.77–7.72 (m, 2H), 7.40–7.34 (m, 3H), 7.30–7.27 (m, 2H), 7.21 (d, *J* = 7.5 Hz, 1H), 6.58 (s, 1H), 6.13 (s, 1H), 3.59 (s, 3H). ^13^C­{^1^H} NMR (100 MHz, CDCl_3_): δ 148.4, 134.5, 132.9, 129.9, 129.5, 128.7, 127.0, 126.9, 126.0, 125.8, 124.6, 100.7, 99.8, 55.2. GC–MS (*m*/*z*, rel. int. %): 274 (9), 272 (M^+^, 28), 241 (100), 206 (14), 178 (29), 111 (8).

#### 3-(4-Fluorophenyl)-1-methoxy-1*H*-isochromene (**2c**)^72^

White solid, (60.2 mg, 0.24 mmol, 94% yield), mp 85.2–86.7 °C. The reaction was purified through recrystallization using petroleum ether. ^1^H NMR (400 MHz, CDCl_3_): δ 7.74–7.69 (m, 2H), 7.29 (ddd, *J* = 3.0, 5.8, 7.6 Hz, 1H), 7.22–7.17 (m, 2H), 7.14 (d, *J* = 7.4 Hz, 1H), 7.05–6.99 (m, 2H), 6.47 (s, 1H), 6.07 (s, 1H), 3.53 (s, 3H). ^13^C­{^1^H} NMR (100 MHz, CDCl_3_): δ 163.0 (d, *J* = 248.7 Hz), 148.6, 130.6 (d, *J* = 3.2 Hz), 130.0, 129.5, 126.8, 126.7 (d, *J* = 4.0 Hz), 126.6, 125.8, 124.5, 115.5 (d, *J* = 21.6 Hz), 100.1 (d, *J* = 2.2 Hz), 99.8, 55.2. ^19^F NMR (377 MHz, CDCl_3_): δ 112.4. GC–MS (*m*/*z*, rel. int. %): 256 (M^+^, 28), 225 (100), 198 (22), 196 (24), 177 (12), 95 (10).

#### 1-Methoxy-3-(*p*-tolyl)-1*H*-isochromene (**2d**)^18^

Pale yellow oil, (60.5 mg, 0.24 mmol, 96% yield). The reaction was purified through column chromatography on silica gel flash (elution: toluene). ^1^H NMR (400 MHz, CDCl_3_): δ 7.63–7.60 (m, 2H), 7.24 (ddd, *J* = 2.1, 6.7, 7.6 Hz, 1H), 7.18–7.09 (m, 5H), 6.46 (s, 1H), 6.03 (s, 1H), 3.49 (s, 3H), 2.27 (s, 3H). ^13^C­{^1^H} NMR (100 MHz, CDCl_3_): δ 148.5, 137.8, 130.6, 129.3, 128.4, 128.2, 125.9, 125.4, 124.8, 123.7, 123.4, 98.7, 98.6, 55.0, 20.3. GC–MS (*m*/*z*, rel. int. %): 252 (M^+^, 52), 221 (100), 178 (22), 165 (6), 119 (22), 91 (16).

#### 1-Methoxy-3-(*m*-tolyl)-1*H*-isochromene (**2e**)

Orange oil, (58 mg, 0.23 mmol, 93% yield). The reaction was purified through column chromatography on silica gel flash (elution: petroleum ether and ethyl acetate). ^1^H NMR (400 MHz, DMSO-*d*_6_): δ 7.64–7.56 (m, 2H), 7.33–7.25 (m, 3H), 7.22 (d, *J* = 7.5 Hz, 2H), 7.14 (d, *J* = 7.5 Hz, 1H), 6.79 (s, 1H), 6.18 (s, 1H), 3.44 (s, 3H), 2.31 (s, 3H). ^13^C­{^1^H} NMR (100 MHz, DMSO-*d*_6_): δ 162.27, 148.71, 137.81, 134.01, 130.00, 129.57, 129.29, 128.54, 127.11, 126.46, 126.07, 125.04, 124.20, 121.79, 98.88, 54.61, 21.08. GC–MS (*m*/*z*, rel. int. %): 252 (M^+^, 52), 221 (100), 178 (22), 165 (6), 119 (22), 91 (16). HRMS (ESI­(+)-TOF) *m*/*z*: [M + H]^+^ calcd for C_17_H_16_O_2_^+^, 253.1223; found, 253.1227.

#### 3-Butyl-1-methoxy-1*H*-isochromene (**2f**)^72^

Pale yellow oil, (28.9 mg, 0.13 mmol, 53% yield). The reaction was purified through column chromatography on silica gel flash (elution: toluene). ^1^H NMR (400 MHz, CDCl_3_): δ 7.24–7.17 (m, 1H), 7.25–7.09 (m, 2H), 6.97 (d, *J* = 7.6 Hz, 1H), 5.87 (s, 1H), 5.71 (s, 1H), 3.45 (s, 3H), 2.30–2.17 (m, 2H), 1.58–1.50 (m, 2H), 1.37–1.29 (m, 2H), 0.87 (t, *J* = 7.3 Hz, 3H). ^13^C­{^1^H} NMR (100 MHz, CDCl_3_): δ 154.2, 130.3, 129.2, 126.2, 125.9, 125.8, 123.4, 100.1, 99.6, 55.0, 33.6, 29.1, 22.1, 13.9. GC–MS (*m*/*z*, rel. int. %): 218 (M^+^, 24), 187 (100), 144 (6), 117 (12), 91 (15).

#### 6-Fluoro-1-methoxy-3-phenyl-1*H*-isochromene (**2g**)^71^

White solid, (62.1 mg, 0.24 mmol, 97% yield), mp 76.6–77.8 °C. The reaction was purified through recrystallization using petroleum ether. ^1^H NMR (400 MHz, CDCl_3_): δ 7.82–7.79 (m, 2H), 7.44–7.35 (m, 3H), 7.27–7.23 (m, 1H), 6.97–6.89 (m, 2H), 6.55 (s, 1H), 6.14 (s, 1H), 3.60 (s, 3H). ^13^C­{^1^H} NMR (100 MHz, CDCl_3_): δ 163.4 (d, *J* = 246.3 Hz), 150.5, 134.0, 132.5 (d, *J* = 9.4 Hz), 129.2, 128.5, 127.7 (d, *J* = 9.5 Hz), 125.0, 122.9 (d, *J* = 3.0 Hz), 113.5 (d, *J* = 22.3 Hz), 110.7 (d, *J* = 22.3 Hz), 99.7 (d, *J* = 2.5 Hz), 99.4, 55.2. ^19^F NMR (377 MHz, CDCl_3_): δ 112.3. GC–MS (*m*/*z*, rel. int. %): 256 (M^+^, 23), 225 (100), 198 (22), 196 (24), 177 (12), 73 (19).

#### 3-(4-Chlorophenyl)-6-fluoro-1-methoxy-1*H*-isochromene (**2h**)

White solid, (65.2 mg, 0.22 mmol, 90% yield), mp 99.0–100.0 °C. The reaction was purified through recrystallization using petroleum ether. ^1^H NMR (400 MHz, CDCl_3_): δ 7.75–7.72 (m, 2H), 7.40–7.36 (m, 2H), 7.27–7.23 (m, 1H), 6.98–6.88 (m, 2H), 6.53 (s, 1H), 6.14 (s, 1H), 3.58 (s, 3H). ^13^C­{^1^H} NMR (100 MHz, CDCl_3_): δ 163.4 (d, *J* = 247.0 Hz), 149.4, 134.9, 132.5, 132.2 (d, *J* = 9.6 Hz), 128.8, 127.7 (d, *J* = 9.2 Hz), 126.2, 122.9 (d, *J* = 3.0 Hz), 113.8 (d, *J* = 22.9 Hz), 110.9 (d, *J* = 22.4 Hz), 100.1 (d, *J* = 2.8 Hz), 99.4, 55.3. ^19^F NMR (377 MHz, CDCl_3_): δ 112.1. GC–MS (*m*/*z*, rel. int. %): 290 (M^+^, 24), 259 (100), 224 (16), 203 (25), 196 (37), 73 (31). HRMS (ESI­(+)-TOF) *m*/*z*: [M – OMe]^+^ calcd for C_15_H_9_ClFO^+^, 259.0326; found, 259.0325.

#### 6-Fluoro-3-(4-fluorophenyl)-1-methoxy-1*H*-isochromene (**2i**)

White solid, (60.3 mg, 0.22 mmol, 88% yield), mp 93.2–94.2 °C. The reaction was purified through recrystallization using petroleum ether. ^1^H NMR (400 MHz, CDCl_3_): δ 7.81–7.76 (m, 2H), 7.26–7.23 (m, 1H), 7.13–7.07 (m, 2H), 6.97–6.88 (m, 2H), 6.47 (s, 1H), 6.13 (s, 1H), 3.59 (s, 3H). ^13^C­{^1^H} NMR (100 MHz, CDCl_3_): δ 163.4 (d, *J* = 246.4 Hz), 163.3 (d, *J* = 248.8 Hz), 149.6, 132.4 (d, *J* = 9.5 Hz), 130.2 (d, *J* = 3.3 Hz), 127.7 (d, *J* = 8.9 Hz), 126.9 (d, *J* = 8.0 Hz), 122.8 (d, *J* = 3.1 Hz), 115.6 (d, *J* = 21.6 Hz), 113.6 (d, *J* = 22.4 Hz), 110.7 (d, *J* = 22.4 Hz), 99.5 (d, *J* = 3.0 Hz), 99.4 (d, *J* = 2.4 Hz), 55.2. ^19^F NMR (377 MHz, CDCl_3_): δ 112.2, 111.8. GC–MS (*m*/*z*, rel. int. %): 274 (M^+^, 23), 243 (100), 215 (20), 214 (24), 195 (12), 73 (8). HRMS (ESI­(+)-TOF) *m*/*z*: [M – OMe]^+^ calcd for C_15_H_9_F_2_O^+^, 243.0621; found, 243.0623.

#### 1,7-Dimethoxy-3-phenyl-1*H*-isochromene (**2j**)^72^

White solid, (57.6 mg, 0.22 mmol, 86% yield), mp 92.0–93.0 °C. The reaction was purified through recrystallization using petroleum ether. ^1^H NMR (400 MHz, CDCl_3_): δ 7.81–7.78 (m, 2H), 7.42–7.38 (m, 2H), 7.34–7.30 (m, 1H), 7.16 (d, *J* = 8.4 Hz, 1H), 6.93 (dd, *J* = 8.4, 2.6 Hz, 1H), 6.84 (d, *J* = 2.6 Hz, 1H), 6.58 (s, 1H), 6.10 (s, 1H), 3.84 (s, 3H), 3.60 (s, 3H). ^13^C­{^1^H} NMR (100 MHz, CDCl_3_): δ 158.7, 147.5, 134.6, 128.4, 128.4, 128.3, 125.9, 124.5, 123.3, 115.8, 110.8, 100.1, 99.7, 55.5, 55.2. GC–MS (*m*/*z*, rel. int. %): 268 (M^+^, 35), 237 (100), 221 (38), 207 (66), 165 (19), 73 (62).

#### 3-(4-Chlorophenyl)-1,7-dimethoxy-1*H*-isochromene (**2k**)

White solid, (62.2 mg, 0.20 mmol, 81% yield), mp 95.0–96.0 °C. The reaction was purified through recrystallization using petroleum ether. ^1^H NMR (400 MHz, CDCl_3_): δ 7.72–7.69 (m, 2H), 7.37–7.34 (m, 2H), 7.15 (d, *J* = 8.4 Hz, 1H), 6.93 (dd, *J* = 8.4, 2.6 Hz, 1H), 6.83 (d, *J* = 2.6 Hz, 1H), 6.55 (s, 1H), 6.08 (s, 1H), 3.84 (s, 3H), 3.58 (s, 3H). ^13^C­{^1^H} NMR (100 MHz, CDCl_3_): δ 158.9, 146.4, 134.0, 133.1, 128.6, 128.4, 126.0, 125.7, 122.9, 115.9, 110.8, 100.5, 99.8, 55.5, 55.2. GC–MS (*m*/*z*, rel. int. %): 302 (M^+^, 0), 272 (100), 257 (25), 209 (55), 194 (27), 165 (33). HRMS (ESI­(+)-TOF) *m*/*z*: [M – OMe]^+^ calcd for C_16_H_12_ClO_2_^+^, 271.0526; found, 271.0523.

#### 5-Methoxy-7-phenyl-5*H*-pyrano­[4,3-*b*]­pyridine (**2l**)

Yellow oil, (50.2 mg, 0.21 mmol, 84% yield). The reaction was purified through column chromatography on silica gel flash (elution: toluene). ^1^H NMR (400 MHz, CDCl_3_): δ 8.47 (d, *J* = 3.3 Hz, 1H), 7.78–7.76 (m, 2H), 7.52 (d, *J* = 9.0 Hz, 1H), 7.38–7.30 (m, 3H), 7.09 (dd, *J* = 4.9, 7.6 Hz, 1H), 6.80 (s, 1H), 6.14 (s, 1H), 3.55 (s, 3H). ^13^C­{^1^H} NMR (100 MHz, CDCl_3_): δ 153.8, 148.6, 148.3, 133.2, 132.5, 128.9, 127.6, 124.4, 121.6, 120.3, 99.7, 98.7, 54.5. GC–MS (*m*/*z*, rel. int. %): 239 (M^+^, 28), 209 (22), 208 (100), 180 (26), 152 (10), 77 (16). HRMS (ESI­(+)-TOF) *m*/*z*: [M + H]^+^ calcd for C_15_H_14_NO_2_^+^, 240,1019; found, 240,1024.

#### 2-(1-Methoxy-1*H*-isochromen-3-yl)­propan-2-ol (**2m**)

Colorless oil, (50.6 mg, 0.23 mmol, 92% yield). The reaction was purified through column chromatography on silica gel flash (elution: toluene). ^1^H NMR (400 MHz, DMSO-*d*_6_): δ 7.16 (td, *J* = 1.6, 7.3 Hz, 1H), 7.10–7.08 (m, 1H), 7.04 (td, *J* = 1.2, 7.3 Hz, 1H), 6.98 (dd, *J* = 1.2, 7.6 Hz, 1H), 5.99 (s, 1H), 5.90 (s, 1H), 4.93 (s, 1H), 3.29 (s, 3H), 1.22 (s, 3H), 1.19 (s, 3H). ^13^C­{^1^H} NMR (100 MHz, DMSO-*d*_6_): δ 160.0, 130.6, 129.6, 126.8, 126.5, 126.2, 124.1, 99.4, 96.9, 70.3, 55.2, 29.3, 28.5. HRMS (ESI­(+)-TOF) *m*/*z*: [M – OMe]^+^ calcd for C_12_H_13_O_2_^+^, 189.0916; found, 189.0919.

### General Procedure for the Synthesis of **3a–3e**

The electrochemical reactions were carried out in an undivided cell of 10 mL (glass bottle with plastic screw cap) equipped with a silver anode (99.9%—55 mm × 5 mm) and a carbon cathode (51 mm × 8 mm). Inside the electrochemical cell, the substrate (0.25 mmol), methanol (MeOH) (4 mL), and lithium perchlorate (LiClO_4_) (0.25 mmol—27 mg) were added. A constant current of 6.0 mA at −10 °C was used for 3 h. After the reaction is completed, the reaction mixture was filtered through a Büchner funnel with a porous plate (50 mL) with approximately half of the funnel containing common silica (70–230 mesh ASTM) in ethyl acetate (100 mL). The ethyl acetate solution was extracted with H_2_O (35 mL × 3) and dried over Na_2_SO_4_. After the solvent was removed under reduced pressure, the solids were recrystallized. The products were dried under high vacuum for 12 h, and the necessary analyses were performed.

#### (*Z*)-1-Benzylidene-3-methoxy-1,3-dihydroisobenzofuran (**3a**)^35^

Yellow oil, (52.9 mg, 0.22 mmol, 89% yield). ^1^H NMR (400 MHz, DMSO-*d*_6_): δ 7.81–7.75 (m, 3H), 7.55–7.50 (m, 2H), 7.48–7.44 (m, 1H), 7.37 (t, *J* = 7.7 Hz, 2H), 7.19 (t, *J* = 7.4 Hz, 1H), 6.66 (s, 1H), 6.25 (s, 1H), 3.48 (s, 3H). ^13^C­{^1^H} NMR (100 MHz, DMSO-*d*_6_): δ 153.2, 137.4, 136.2, 135.3, 130.6, 129.7, 128.9, 128.5, 126.3, 123.9, 120.5, 107.9, 98.2, 55.1. GC–MS (*m*/*z*, rel. int. %): 238 (M^+^, 100), 207 (52), 206 (56), 179 (92), 178 (91), 89 (28).

#### (*Z*)-1-(4-Chlorobenzylidene)-3-methoxy-1,3-dihydroisobenzofuran (**3b**)^4^

White solid, (54.4 mg, 0.20 mmol, 80% yield), mp 72.2–73.5 °C. The reaction was purified through recrystallization using petroleum ether. ^1^H NMR (400 MHz, DMSO-*d*_6_): δ 7.82 (d, *J* = 7.8 Hz, 1H), 7.78 (d, *J* = 8.6 Hz, 2H), 7.59–7.55 (m, 2H), 7.52–7.48 (m, 1H), 7.45 (d, *J* = 8.6 Hz, 2H), 6.71 (s, 1H), 6.30 (s, 1H), 3.50 (s, 3H). ^13^C­{^1^H} NMR (100 MHz, DMSO-*d*_6_): δ 153.8, 137.5, 135.2, 135.0, 130.6, 130.4, 130.0, 129.9, 128.9, 123.9, 120.6, 108.1, 97.0, 55.1. GC–MS (*m*/*z*, rel. int. %): 274 (8), 272 (M^+^, 23), 241 (13), 240 (9), 178 (19), 44 (100).

#### (*Z*)-1-(4-Fluorobenzylidene)-3-methoxy-1,3-dihydroisobenzofuran (**3c**)

Yellow oil, (53.8 mg, 0.21 mmol, 84% yield). ^1^H NMR (400 MHz, DMSO-*d*_6_): δ 7.80–7.75 (m, 3H), 7.52–7.49 (m, 2H), 7.46–7.42 (m, 1H), 7.19 (d, *J* = 8.9 Hz, 2H), 6.64 (s, 1H), 6.25 (s, 1H), 3.46 (s, 3H). ^13^C­{^1^H} NMR (100 MHz, DMSO-*d*_6_): δ 160.8 (d, *J* = 243.9 Hz), 152.9 (d, *J* = 2.9 Hz), 137.1, 135.2, 132.7 (d, *J* = 3.1 Hz), 130.6, 130.2 (d, *J* = 7.7 Hz), 129.7, 123.9, 120.4, 115.8 (d, *J* = 21.2 Hz), 107.9, 97.1, 55.0. ^19^F NMR (377 MHz, CDCl_3_): δ 115.8. GC–MS (*m*/*z*, rel. int. %): 256 (M^+^, 100), 224 (53), 223 (50), 196 (67), 177 (19), 98 (14). HRMS (ESI­(+)-TOF) *m*/*z*: [M + H]^+^ calcd for C_16_H_13_FO_2_^+^, 257.0972; found, 257.0972.

#### (*Z*)-3-(4-Chlorobenzylidene)-5-fluoro-1-methoxy-1,3-dihydroisobenzofuran (**3d**)

White solid, (63.1 mg, 0.22 mmol, 87% yield), mp 103.6–104.8 °C. The reaction was purified through recrystallization using petroleum ether. ^1^H NMR (400 MHz, DMSO-*d*_6_): δ 7.73–7.68 (m, 3H), 7.56 (dd, *J* = 4.9, 8.3 Hz, 2H), 7.45–7.41 (m, 2H), 7.32–7.27 (m, 1H), 6.65 (s, 1H), 6.34 (s, 1H), 3.47 (s, 3H). ^13^C­{^1^H} NMR (100 MHz, DMSO-*d*_6_): δ 164.0 (d, *J* = 245.0 Hz), 152.8 (d, *J* = 3.6 Hz), 137.6 (d, *J* = 10.4 Hz), 134.8, 133.6, 130.8, 130.1, 129.0, 126.1 (d, *J* = 9.9 Hz), 117.4 (d, *J* = 24.2 Hz), 107.7, 107.4 (d, *J* = 24.8 Hz), 98.2, 55.2. ^19^F NMR (377 MHz, CDCl_3_): δ 111.7. GC–MS (*m*/*z*, rel. int. %): 290 (M^+^, 60), 259 (32), 223 (21), 207 (33), 196 (42), 44 (100). HRMS (ESI­(+)-TOF) *m*/*z*: [M – OMe]^+^ calcd for C_15_H_9_ClFO^+^, 259,0326; found, 259.0327.

#### (*Z*)-1-Methoxy-3-(4-methylbenzylidene)-1,3-dihydroisobenzofuran (**3e**)^35^

Yellow oil, (53.5 mg, 0.21 mmol, 42% yield). ^1^H NMR (400 MHz, DMSO-*d*_6_): δ 7.82 (d, *J* = 8.6 Hz, 1H), 7.69 (d, *J* = 8.2 Hz, 2H), 7.60–7.55 (m, 2H), 7.50 (dd, *J* = 1.0, 7.2 Hz, 1H), 7.23 (d, *J* = 7.9 Hz, 2H), 6.70 (s, 1H), 6.25 (s, 1H), 3.51 (s, 3H), 2.35 (s, 3H). ^13^C­{^1^H} NMR (100 MHz, DMSO-*d*_6_): δ 152.5, 137.2, 135.5, 135.4, 133.3, 130.6, 129.6, 129.5, 128.5, 123.8, 120.4, 107.7, 98.2, 55.0, 21.3. GC–MS (*m*/*z*, rel. int. %): 252 (M^+^, 100), 221 (36), 220 (37), 193 (47), 178 (52), 165 (17).

## Supplementary Material



## Data Availability

The data underlying this study are available in the published article and its Supporting Information.

## Notes

General procedure; scaling up synthesis; general procedure for cyclic voltammetry; control experiments; calculation of silver release; characterization data of products; ^1^H and ^13^C NMR spectra of all synthesized compounds; HRMS and theoretical calculations (PDF)
