# Supplementary material for: Isochromene and Dihydroisobenzofuran Electrosynthesis by a Highly Regiodivergent Cyclization of 2‑Ethynylbenzaldehydes
Source: J Org Chem. 2025 Jun 30;90(27):9532–40. doi: 10.1021/acs.joc.5c00967 (PMC12261327; doi:10.1021/acs.joc.5c00967)
Supplement: Supplementary file 1 [file jo5c00967_si_001.pdf]

Isochromene and Dihydroisobenzofuran Electrosynthesis by a Highly Regiodivergent Cyclization of 2-Ethynylbenzaldehydes

Guilherme M. Martins,*[a] Guilherme B. Simoso,[a] Pedro P. de Castro,[a,b] Felipe de Lucca,[a] Samuel R. Mendes,[c] Timothy J. Brocksom[a] and Kleber T. de Oliveira*[a]

[a] Department of Chemistry, Federal University of São Carlos, São Carlos, São Paulo, 13565-905, Brazil.
[b] Department of Pharmacy, Federal University of Juiz de Fora – Campus Governador Valadares, Governador Valadares, Minas Gerais, 35010-177, Brazil.
[c] Department of Chemistry, State University of Santa Catarina, Joinville, Santa Catarina, 89219-719, Brazil.

*E-mail: guilherme.martins@ufscar.br, kleber.oliveira@ufscar.br

Table of Contents

1. GENERAL CONSIDERATIONS S2
2. GENERAL PROCEDURE S3
2.1 Compounds 2a-2m S3
2.2 Compounds 3a-3e S4
3. SCALING UP SYNTHESIS PROCEDURE FOR 2a S5
4. GENERAL PROCEDURE FOR CYCLIC VOLTAMMETRY S6
5. CONTROL EXPERIMENTS S7
6. ELETRIC CHARGE S10
7. CALCULATION OF SILVER RELEASE S11
8. CHARACTERIZATION DATA OF PRODUCTS S12
8.1 Compounds 2a-2m S12
8.2 Compounds 3a-3e S16
9. NMR SPECTRA S19
9.1 Compounds 2a-2m S19
9.2 Compounds 3a-3e S34
10. THEORETICAL CALCULATIONS - GENERAL REMARKS S40
11. GENERAL OVERVIEW OF THE EVALUATED MECHANISMS S41
12. ENERGY PROFILE (ΔG) OF THE EVALUATED MECHANISMS S44
13. IMAGINARY FREQUENCIES FOR ALL TRANSITION STATES, INTRINSIC REACTION COORDINATES AND SCAN DATA S49
14. ELECTRONIC ENERGIES (E), ENTHALPIES (H) AND GIBBS FREE ENERGIES (G) OF ALL OPTIMIZED STRUCTURES S57
15. ELECTRONIC ENERGIES (ΔE), ENTHALPIES (ΔH) AND GIBBS FREE ENERGIES (ΔG) VARIATION ALONG THE REACTION PATHWAY S60
16. COORDINATES OF OPTIMIZED STATIONARY POINTS S63
17. REFERENCES S80

1. GENERAL CONSIDERATIONS

All purchased chemicals were used as received without further purification. Analytical TLC was performed on TLC plates (silica gel 60 F254) and visualized employing a UV lamp and/or acidic ethanolic vanillin solution (5% in 10% H_2SO_4) as a revelator. Yields refer to purified compounds which are spectroscopically pure. Both ^1H and $^{13}C\{^1H\}$ NMR spectra were recorded at 400 and 100 MHz, respectively. Chemical shifts are informed in ppm downfield from the signal of TMS, used as an internal standard, and the coupling constants (J) are expressed in Hertz (Hz). Chemical shifts are reported employing the following abbreviation pattern: s (singlet), d (doublet), dd (doublet of doublet), dt (doublet of triplet), t (triplet), q (quartet), and m (multiplet). Low-resolution mass spectra were obtained from a Shimadzu GC-MS-QP2020 NX mass spectrometer.

Substituted 2-ethynylbenzaldehydes were prepared following literature protocols.[1–3] The electrochemical reactions were carried out using a power supply (AFR – model FA3005P).

Figure S1. The power source used in the electrochemical reactions.

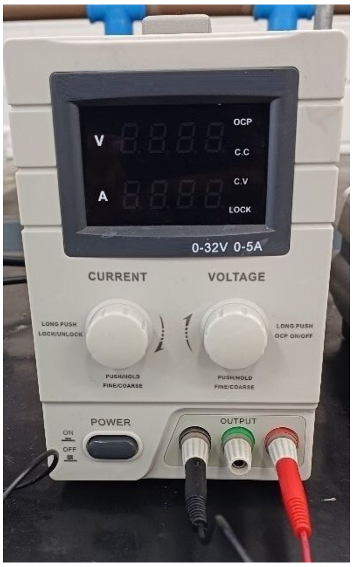

2. GENERAL PROCEDURE
2.1 Compounds 2a-2m

The electrochemical reactions were carried out in an undivided cell of 10 mL (glass bottle with plastic screw cap) equipped with a silver anode (99.9% - 55 mm x 5 mm) and a carbon cathode (51 mm x 8 mm), with an interelectrode distance of 7.5 mm, as shown in **Figure S2**. Inside the electrochemical cell, the substrate (0.25 mmol), dimethylformamide (DMF) (2 mL), methanol (MeOH) (2 mL), lithium perchlorate ($LiClO_4$) (0.25 mmol - 27 mg), and acetic acid (AcOH) (0.25 mmol - 14 µL) were added. A constant current of 6.0 mA at 60 °C was used for 3 hours. After the reaction, the reaction mixture was filtered through a Büchner funnel with a porous plate (50 mL) with approximately half of the funnel containing common silica (70-230 mesh ASTM) in ethyl acetate (100 mL). The ethyl acetate solution was extracted with H_2O (35 mL x 3) and dried over Na_2SO_4. After removal of the solvent under reduced pressure, some compounds were recrystallized or purified by chromatography on silica gel flash (elution: toluene). The products were dried under a high vacuum and the necessary analyses were performed.

Figure S2. Electrochemical cell and reactional system for **2a-2m** compounds.

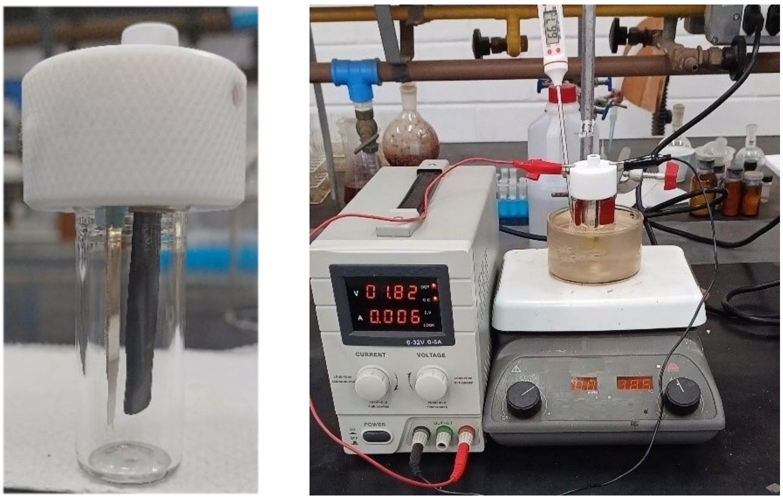

Figure S3. General scheme for the synthesis of **2a-2m** compounds.

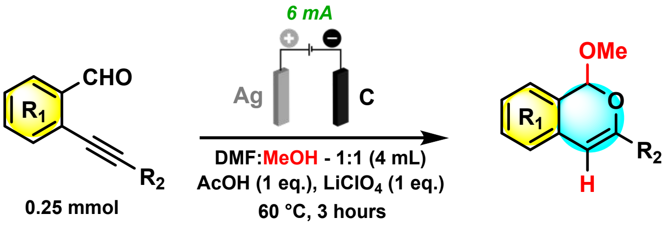

2.2 Compounds 3a-3e

The electrochemical reactions were carried out in an undivided cell of 10 mL (glass bottle with plastic screw cap) equipped with a silver anode (99.9% - 55 mm x 5 mm) and a carbon cathode (51 mm x 8 mm), as shown in **Figure S4**. Inside the electrochemical cell, the substrate (0.25 mmol), methanol (MeOH) (4 mL), and lithium perchlorate ($LiClO_4$) (0.25 mmol - 27 mg) were added. A constant current of 6.0 mA at -10 °C was used for 3 hours. After the reaction is completed, the reaction mixture was filtered through a Büchner funnel with a porous plate (50 mL) with approximately half of the funnel containing common silica (70-230 mesh ASTM) in ethyl acetate (100 mL). The ethyl acetate solution was extracted with H_2O (35 mL x 3) and dried over Na_2SO_4. After the solvent was removed under reduced pressure, the solids were recrystallized. The products were dried under high vacuum for 12 hours, and the necessary analyses were performed.

Figure S4. Electrochemical cell and reactional system for **3a-3e** compounds.

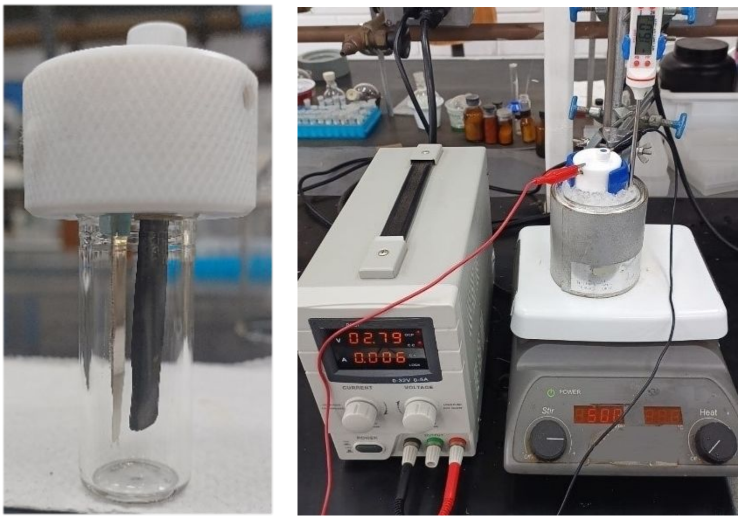

Figure S5. General scheme for the synthesis of **3a-3e** compounds.

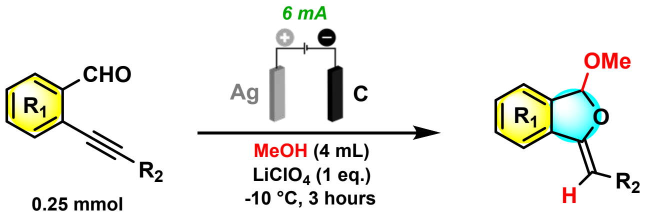

3. SCALING UP SYNTHESIS PROCEDURE FOR 2a

The scaling up of the electrochemical reaction was carried out in an undivided cell of 100 mL (glass bottle with plastic screw cap) equipped with three silver anodes (99.9% - 70 mm x 5 mm, totaling 70 mm x 15 mm) and one carbon cathode (53 mm x 25 mm), as shown in **Figure S6**. Inside the electrochemical cell, 2-(phenylethynyl)benzaldehyde (**1a**) (5 mmol – 1.03 mg), dimethylformamide (DMF) (30 mL), methanol (MeOH) (30 mL), lithium perchlorate ($LiClO_4$) (5 mmol - 540 mg), and acetic acid (AcOH) (5 mmol - 28 μL) were added. A constant current of 6.0 mA at 60 °C was used for 30 hours. After the reaction is completed, the reaction mixture was filtered through a Büchner funnel with a porous plate (50 mL) with approximately half of the funnel containing common silica (70-230 mesh ASTM) in ethyl acetate (500 mL). The ethyl acetate solution was extracted with H_2O (170 mL x 3) and dried over Na_2SO_4. After the solvent was removed under reduced pressure, the product was purified through column chromatography on silica gel flash (elution: toluene) to afford product **2a** as a white solid (1.059 mg, 4.45 mmol, 89% yield).

Figure S6. Reactional system of the scaling up of **2a**.

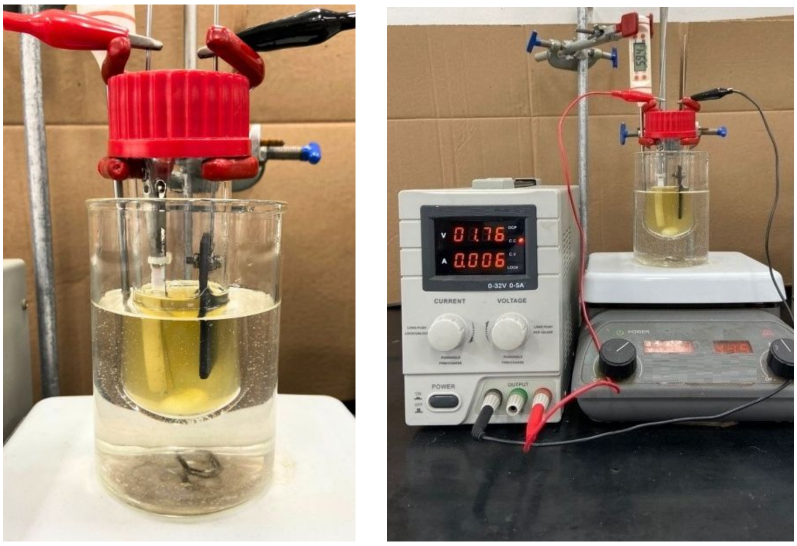

4. GENERAL PROCEDURE FOR CYCLIC VOLTAMMETRY

Cyclic voltammetry was performed with a conventional three electrode electrochemical cell with IKA ElectraSyn 2.0, charting with IUPAC. The redox properties of each compound (2-(phenylethynyl)benzaldehyde (**1a**), 1-methoxy-3-phenyl-1*H*-isochromene (**2a**), (*Z*)-1-benzylidene-3-methoxy-1,3-dihydroisobenzofuran (**3a**)) were measured in anhydrous acetonitrile (CH_3CN) containing lithium perchlorate ($LiClO_4$) at 0.06 mol·L^{-1} as the supporting electrolyte at room temperature. Prior to use, the acetonitrile solvent was thoroughly degassed by three freeze-pump-thaw (FPT) cycles to remove dissolved oxygen and other gases. The working electrode was a glassy carbon (GC) electrode, and no polishing was performed prior to use. The counter electrode was also a carbon electrode from the same CV kit. The reference electrode consisted of an Ag/Ag^+ electrode filled with a 3 M KCl solution, containing a silver wire as the internal reference. The sample concentration was adjusted to 0.06 mol·L^{-1}, and the potential scan was conducted from –1.8 V to +1.8 V at a scan rate of 10 mV·s^{-1}.

Figure S7. Cyclic voltammogram of **1a**, **2a** and **3a**.

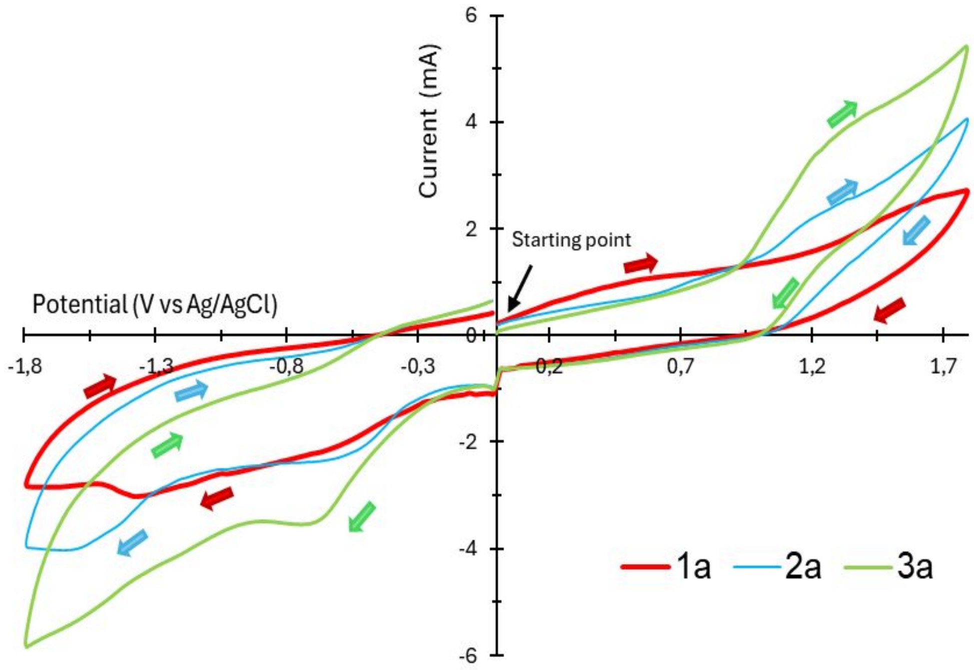

5. CONTROL EXPERIMENTS

Figure S8. Control experiments. Standard conditions: **1a** (0.25 mmol), $LiClO_4$ (0.25 mmol, 1 eq.), AcOH (0.25 mmol, 1 eq) in MeOH:DMF (1:1, 0.06M), Ag anode, C cathode, undivided cell, constant current = 6 mA, at 60 °C under air for 3 h. Isolated yield.

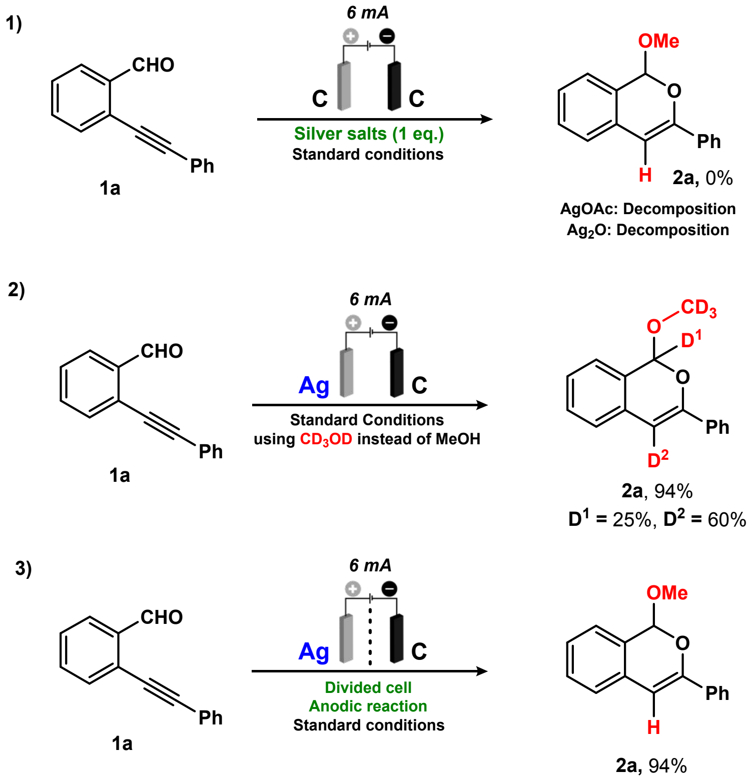

Figure S9. Divided-cell setup.

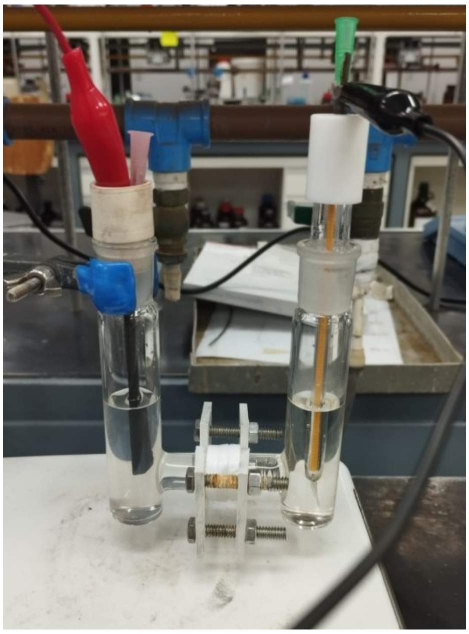

Table S1. ON/OFF Experiments.

Current	Reaction time (min)	Product 2a (%)	Starting material 1a (%)
ON	0	0	100
ON	30	24,19	75,81
OFF	45	34,42	66,58
OFF	60	34,75	65,25
OFF	75	36,57	63,43
ON	90	57,97	42,03
ON	105	62,74	37,26
ON	120	72,27	27,73

Figure S10. ON/OFF Diagram.

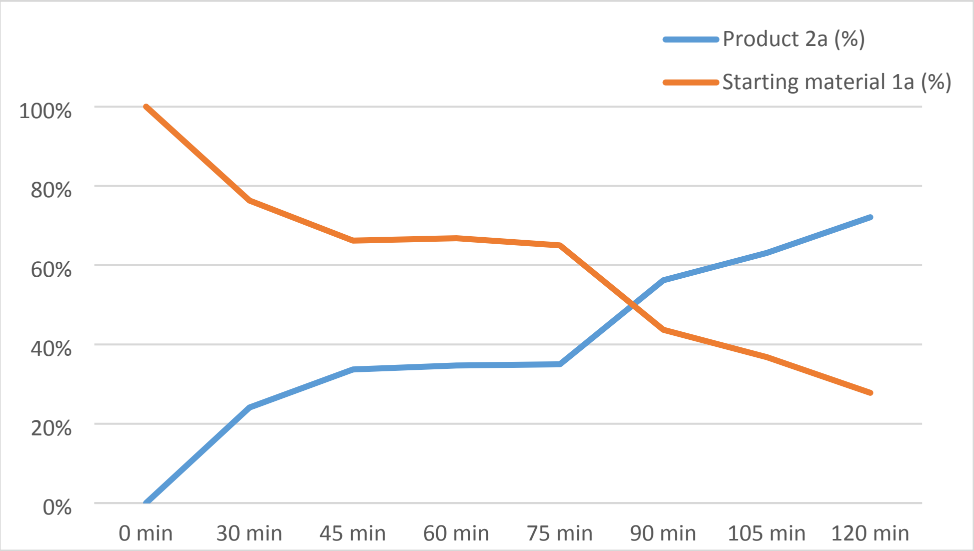

Figure S11. GC-MS analysis of the ON/OFF experiments.

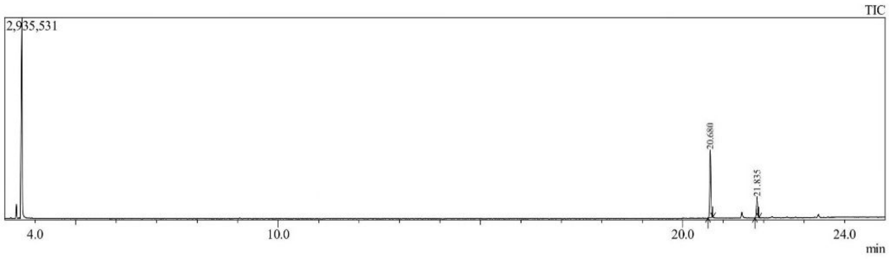

Peak Report TIC

Peak#	R.Time	I.Time	F.Time	Area	Area%	Height	Height%	A/H	Mark	Name
1	20.680	20.625	20.745	1947726	75.81	977647	76.34	1.99	MI	MP
2	21.835	21.785	21.885	621661	24.19	302937	23.66	2.05	MI	P
				2569387	100.00	1280584	100.00			

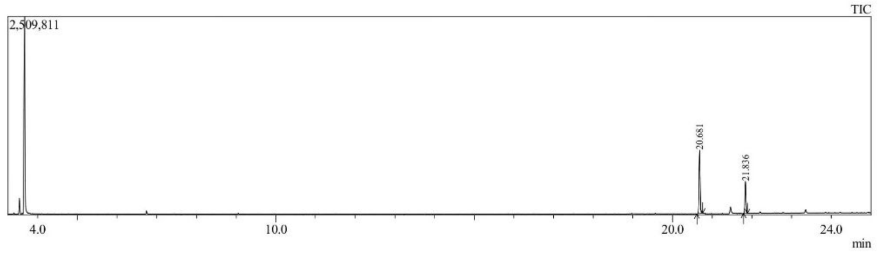

Peak Report TIC

Peak#	R.Time	I.Time	F.Time	Area	Area%	Height	Height%	A/H	Mark	Name
1	20.681	20.615	20.755	1604830	66.58	799532	66.29	2.01	MI	MP
2	21.836	21.790	21.885	805591	33.42	406598	33.71	1.98	MI	P
				2410421	100.00	1206130	100.00			

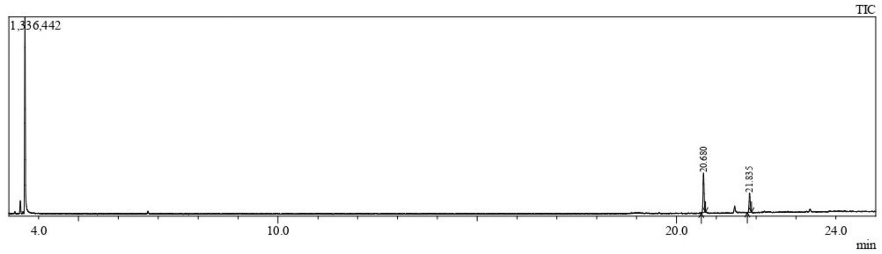

Peak Report TIC

Peak#	R.Time	I.Time	F.Time	Area	Area%	Height	Height%	A/H	Mark	Name
1	20.680	20.625	20.730	505414	65.25	263215	66.87	1.92	MI	MP
2	21.835	21.780	21.880	269191	34.75	130413	33.13	2.06	MI	P
				774605	100.00	393628	100.00			

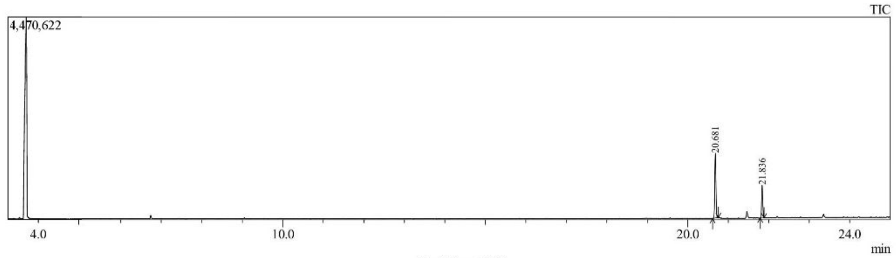

Peak Report TIC

Peak#	R.Time	I.Time	F.Time	Area	Area%	Height	Height%	A/H	Mark	Name
1	20.681	20.625	20.740	2820302	36.57	1401720	38.07	2.01	MI	MP
2	21.836	21.790	21.905	4892116	63.43	2280136	61.93	2.15	MI	P
				7712418	100.00	3681856	100.00			

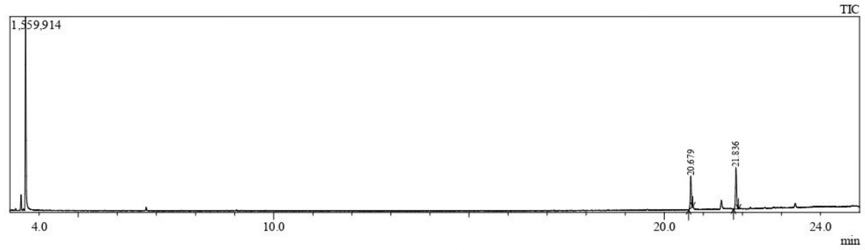

Peak Report TIC

Peak#	R.Time	I.Time	F.Time	Area	Area%	Height	Height%	A/H	Mark	Name
1	20.679	20.625	20.730	493594	42.03	256417	43.79	1.92	MI	MP
2	21.836	21.770	21.900	680792	57.97	329124	56.21	2.07	MI	P
				1174386	100.00	585541	100.00			

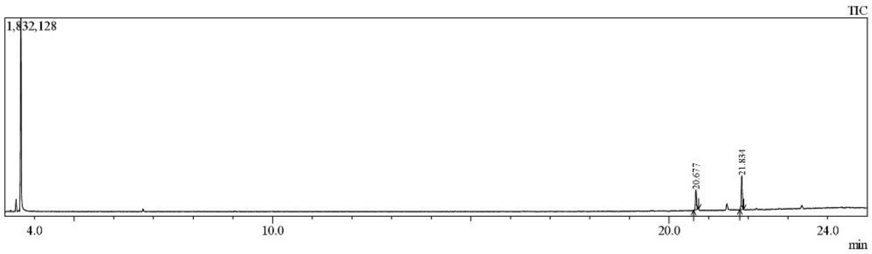

Peak Report TIC

Peak#	R.Time	I.Time	F.Time	Area	Area%	Height	Height%	A/H	Mark	Name
1	20.677	20.620	20.745	368671	37.26	182273	36.83	2.02	MI	
2	21.834	21.785	21.875	620897	62.74	312606	63.17	1.99	MI	
				989568	100.00	494879	100.00			

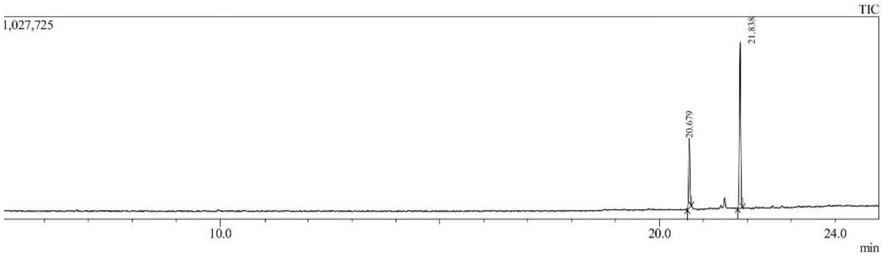

Peak Report TIC

Peak#	R.Time	I.Time	F.Time	Area	Area%	Height	Height%	A/H	Mark	Name
1	20.679	20.635	20.725	677726	27.73	352863	29.48	1.92	MI	MP
2	21.838	21.785	21.895	1765876	72.27	844074	70.52	2.09	MI	P
				2443602	100.00	1196937	100.00			

6. ELETRIC CHARGE

The Faradays per mole (F/mol) represents the amount of electric charge required to drive an electrochemical reaction per mole of substance. This is based on Faraday's constant, which defines the charge of one mole of electrons as approximately 96,485 Coulombs. The value of F/mol provides insight into how many moles of electrons are transferred in each reaction, helping to quantify the relationship between the applied charge and the amount of material undergoing electrochemical conversion. The calculation of F/mol is crucial for understanding the efficiency and stoichiometry of electrochemical processes.[4]

To calculate, the total charge (Q) is required, which is expressed in Coulombs (C) or Ampere-seconds (A·s). This is determined by multiplying the current (I) in amperes by the time (t) in seconds. Additionally, the amount of reagent used (n), in mols, is needed. For illustration, our reactions were carried out over 3 hours (or 10,800 seconds) with a current of 6 mA (or $6 \cdot 10^{-3}$ A), and the total charge is:

$$Q = I \times t = 6 \times 10^{-3}\, A \times 10800\, s = 64{,}8\, C$$

A total of 0.25 mmol or 2.5×10^{-4} mol of reagent was used. The charge per mole of reagent is:

$$\frac{Q}{n} = \frac{64{,}8\, C}{2{,}5 \times 10^{-4}} = 259200\, {}^{C}/_{mol}$$

Normalizing by the Faraday's constant:

$${}^{F}/_{mol} = \frac{259200\ C/mol}{F} = \frac{259200\ C/mol}{96485\ C/mol} \approx 2.69$$

7. CALCULATION OF SILVER RELEASE

To calculate the release of silver during the reaction, we can weigh the electrode before and after the reaction or use the equation that relates to Faraday's laws:

$$n = \frac{Q}{ne \times F}$$

Where *n* is the amount of silver released in mols, *Q* is the charge in Coulombs, *ne* is the number of electrons in the reaction, and *F* is Faraday's constant.:

$$n = \frac{64{,}8\ C}{1 \times 96485\ C/mol} = 0{,}000672\ mol\ or\ 0{,}672\ mmol$$

This amount of silver corresponds to 2.69 equivalents relative to the reagent concentration.

8. CHARACTERIZATION DATA OF PRODUCTS

8.1 Compounds 2a-2m

1-methoxy-3-phenyl-1H-isochromene[5] ***2a***

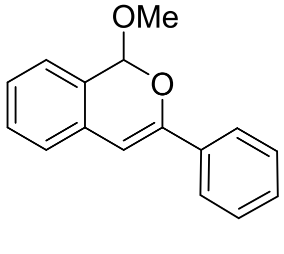

White solid, (56.5 mg, 0.24 mmol, 93% yield), m.p.: 51.1 – 52.2 °C. The reaction was purified through recrystallization using petroleum ether. ^{1}H NMR (400 MHz, $CDCl_3$): δ = 7.75 (d, *J* = 7.4 Hz, 2H), 7.36 – 7.26 (m, 4H), 7.23 – 7.15 (m, 3H), 6.55 (s, 1H), 6.08 (s, 1H), 3.54 (s, 3H). ^{13}C{^{1}H} NMR (100 MHz, $CDCl_3$): δ = 148.4, 133.4, 129.2, 128.5, 127.8, 127.5, 126.0, 125.7, 124.8, 123.8, 123.5, 99.4, 98.7, 54.2. GC-MS (*m/z*, rel. int. %): 238 (M^+, 24), 207 (100), 178 (40), 165 (10), 105 (14), 77 (22).

3-(4-chlorophenyl)-1-methoxy-1H-isochromene[6] ***2b***

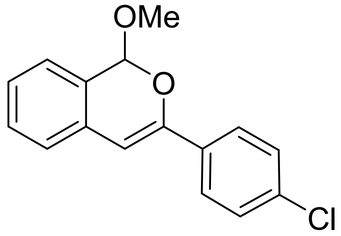

White solid, (61.2 mg, 0.22 mmol, 90% yield), m.p.: 108.2 – 109.2 °C. The reaction was purified through recrystallization using petroleum ether. ^{1}H NMR (400 MHz, $CDCl_3$): δ = 7.77 – 7.72 (m, 2H), 7.40 – 7.34 (m, 3H), 7.30-7.27 (m, 2H), 7.21 (d, *J* = 7.5 Hz, 1H), 6.58 (s, 1H), 6.13 (s, 1H), 3.59 (s, 3H). ^{13}C{^{1}H} NMR (100 MHz, $CDCl_3$): δ = 148.4, 134.5, 132.9, 129.9, 129.5, 128.7, 127.0, 126.9, 126.0, 125.8, 124.6, 100.7, 99.8, 55.2. GC-MS (*m/z*, rel. int. %): 274 (9), 272 (M^+, 28), 241 (100), 206 (14), 178 (29), 111 (8).

3-(4-fluorophenyl)-1-methoxy-1H-isochromene[7] ***2c***

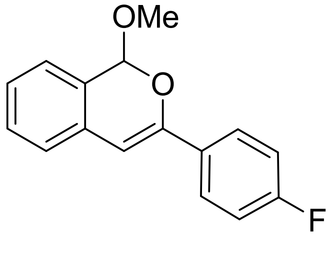

White solid, (60.2 mg, 0.24 mmol, 94% yield), m.p.: 85.2 – 86.7 °C. The reaction was purified through recrystallization using petroleum ether. ^{1}H NMR (400 MHz, $CDCl_3$): δ = 7.74 – 7.69 (m, 2H), 7.29 (ddd, *J* = 3.0, 5.8, 7.6 Hz, 1H), 7.22 – 7.17 (m, 2H), 7.14 (d, *J* = 7.4 Hz, 1H), 7.05 – 6.99 (m, 2H), 6.47 (s, 1H), 6.07 (s, 1H), 3.53 (s, 3H). ^{13}C{^{1}H} NMR (100 MHz, $CDCl_3$): δ = 163.0 (d, *J* = 248.7 Hz), 148.6, 130.6 (d, *J* = 3.2 Hz), 130.0, 129.5, 126.8, 126.7 (d, *J* = 4.0 Hz), 126.6, 125.8, 124.5, 115.5 (d, *J* = 21.6 Hz), 100.1 (d, *J* = 2.2 Hz), 99.8, 55.2. ^{19}F NMR (377 MHz, $CDCl_3$): δ = 112.4. GC-MS (*m/z*, rel. int. %): 256 (M^+, 28), 225 (100), 198 (22), 196 (24), 177 (12), 95 (10).

1-methoxy-3-(p-tolyl)-1H-isochromene[8] ***2d***

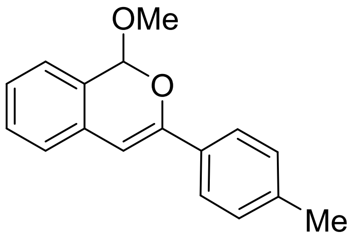

Pale yellow oil, (60.5 mg, 0.24 mmol, 96% yield). The reaction was purified through column chromatography on silica gel flash (elution: toluene). ^{1}H NMR (400 MHz, $CDCl_3$): δ = 7.63 – 7.60 (m, 2H), 7.24 (ddd, *J* = 2.1, 6.7, 7.6 Hz, 1H), 7.18 – 7.09 (m, 5H), 6.46 (s, 1H), 6.03 (s, 1H), 3.49 (s, 3H), 2.27 (s, 3H). $^{13}C\{^1H\}$ NMR (100 MHz, $CDCl_3$): δ = 148.5, 137.8, 130.6, 129.3, 128.4, 128.2, 125.9, 125.4, 124.8, 123.7, 123.4, 98.7, 98.6, 55.0, 20.3. GC-MS (*m/z*, rel. int. %): 252 (M^+, 52), 221 (100), 178 (22), 165 (6), 119 (22), 91 (16).

1-methoxy-3-(m-tolyl)-1H-isochromene ***2e***

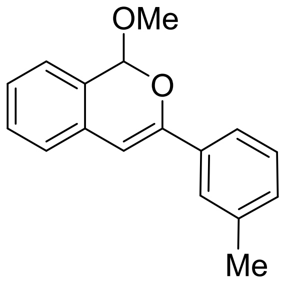

Orange oil, (58 mg, 0.23 mmol, 93% yield). The reaction was purified through column chromatography on silica gel flash (elution: petroleum ether and ethyl acetate). ^{1}H NMR (400 MHz, DMSO-d_6) δ 7.64 – 7.56 (m, 2H), 7.33 – 7.25 (m, 3H), 7.22 (d, *J* = 7.5 Hz, 2H), 7.14 (d, *J* = 7.5 Hz, 1H), 6.79 (s, 1H), 6.18 (s, 1H), 3.44 (s, 3H), 2.31 (s, 3H). $^{13}C\{^1H\}$ NMR (100 MHz, DMSO-d_6): δ = 162.27, 148.71, 137.81, 134.01, 130.00, 129.57, 129.29, 128.54, 127.11, 126.46, 126.07, 125.04, 124.20, 121.79, 98.88, 54.61, 21.08. GC-MS (*m/z*, rel. int. %): 252 (M+, 52), 221 (100), 178 (22), 165 (6), 119 (22), 91 (16). HRMS (ESI(+)-TOF) *m/z*: $[M+H]^+$ Calcd for $C_{17}H_{16}O_2^+$ 253.1223; Found 253.1227.

3-butyl-1-methoxy-1H-isochromene[7] ***2f***

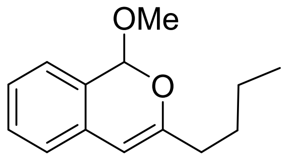

Pale yellow oil, (28.9 mg, 0.13 mmol, 53% yield). The reaction was purified through column chromatography on silica gel flash (elution: toluene). ^{1}H NMR (400 MHz, $CDCl_3$): δ = 7.24 – 7.17 (m, 1H), 7.25-7.09 (m, 2H), 6.97 (d, *J* = 7.6 Hz, 1H), 5.87 (s, 1H), 5.71 (s, 1H), 3.45 (s, 3H), 2.30-2.17 (m, 2H), 1.58 – 1.50 (m, 2H), 1.37 – 1.29 (m, 2H), 0.87 (t, *J* = 7.3 Hz, 3H). $^{13}C\{^1H\}$ NMR (100 MHz, $CDCl_3$): δ = 154.2, 130.3, 129.2, 126.2, 125.9, 125.8, 123.4, 100.1, 99.6, 55.0, 33.6, 29.1, 22.1, 13.9. GC-MS (*m/z*, rel. int. %): 218 (M^+, 24), 187 (100), 144 (6), 117 (12), 91 (15).

6-fluoro-1-methoxy-3-phenyl-1H-isochromene[6] ***2g***

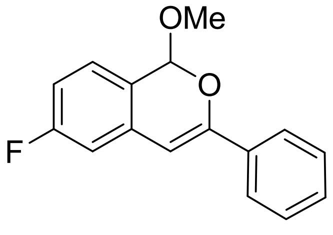

White solid, (62.1 mg, 0.24 mmol, 97% yield), m.p.: 76.6 – 77.8 °C. The reaction was purified through recrystallization using petroleum ether. ^{1}H NMR (400 MHz, $CDCl_3$): δ = 7.82-7.79 (m, 2H), 7.44 – 7.35 (m, 3H), 7.27 – 7.23 (m, 1H), 6.97-6.89 (m, 2H), 6.55 (s, 1H), 6.14 (s, 1H), 3.60 (s, 3H). ^{13}C {^{1}H}NMR (100 MHz, $CDCl_3$): δ = 163.4 (d, *J* = 246.3 Hz), 150.5, 134.0, 132.5 (d, *J* = 9.4 Hz), 129.2, 128.5, 127.7 (d, *J* = 9.5 Hz), 125.0, 122.9 (d, *J* = 3.0 Hz), 113.5 (d, *J* = 22.3 Hz), 110.7 (d, *J* = 22.3 Hz), 99.7 (d, *J* = 2.5 Hz), 99.4, 55.2. ^{19}F NMR (377 MHz, $CDCl_3$): δ = 112.3. GC-MS (*m/z*, rel. int. %): 256 (M^+, 23), 225 (100), 198 (22), 196 (24), 177 (12), 73 (19).

3-(4-chlorophenyl)-6-fluoro-1-methoxy-1H-isochromene ***2h***

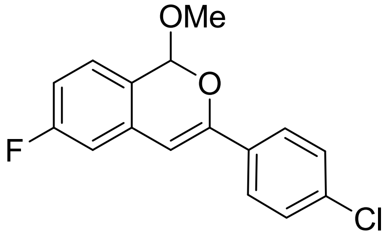

White solid, (65.2 mg, 0.22 mmol, 90% yield), m.p.: 99.0 – 100.0 °C. The reaction was purified through recrystallization using petroleum ether. ^{1}H NMR (400 MHz, $CDCl_3$): δ = 7.75-7.72 (m, 2H), 7.40-7.36 (m, 2H), 7.27-7.23 (m, 1H), 6.98-6.88 (m, 2H), 6.53 (s, 1H), 6.14 (s, 1H), 3.58 (s, 3H). ^{13}C{^{1}H} NMR (100 MHz, $CDCl_3$): δ = 163.4 (d, *J* = 247.0 Hz), 149.4, 134.9, 132.5, 132.2 (d, *J* = 9.6 Hz), 128.8, 127.7 (d, *J* = 9.2 Hz), 126.2, 122.9 (d, *J* = 3.0 Hz), 113.8 (d, *J* = 22.9 Hz), 110.9 (d, *J* = 22.4 Hz), 100.1 (d, *J* = 2.8 Hz), 99.4, 55.3. ^{19}F NMR (377 MHz, $CDCl_3$): δ = 112.1. GC-MS (*m/z*, rel. int. %): 290 (M^+, 24), 259 (100), 224 (16), 203 (25), 196 (37), 73 (31). HRMS (ESI(+)-TOF) *m/z*: $[M\text{-}OMe]^+$ Calcd for $C_{15}H_9ClFO^+$ 259.0326; Found 259.0325.

6-fluoro-3-(4-fluorophenyl)-1-methoxy-1H-isochromene ***2i***

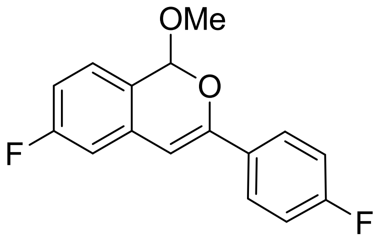

White solid, (60.3 mg, 0.22 mmol, 88% yield), m.p.: 93.2 – 94.2 °C. The reaction was purified through recrystallization using petroleum ether. ^{1}H NMR (400 MHz, $CDCl_3$): δ = 7.81-7.76 (m, 2H), 7.26-7.23 (m, 1H),

7.13-7.07 (m, 2H), 6.97-6.88 (m, 2H), 6.47 (s, 1H), 6.13 (s, 1H), 3.59 (s, 3H). $^{13}C\{^1H\}$ NMR (100 MHz, $CDCl_3$): δ = 163.4 (d, *J* = 246.4 Hz), 163.3 (d, *J* = 248.8 Hz), 149.6, 132.4 (d, *J* = 9.5 Hz), 130.2 (d, *J* = 3.3 Hz), 127.7 (d, *J* = 8.9 Hz), 126.9 (d, *J* = 8.0 Hz), 122.8 (d, *J* = 3.1 Hz), 115.6 (d, *J* = 21.6 Hz), 113.6 (d, *J* = 22.4 Hz), 110.7 (d, *J* = 22.4 Hz), 99.5 (d, *J* = 3.0 Hz), 99.4 (d, *J* = 2.4 Hz), 55.2. ^{19}F NMR (377 MHz, $CDCl_3$): δ = 112.2, 111.8. GC-MS (*m/z*, rel. int. %): 274 (M^+, 23), 243 (100), 215 (20), 214 (24), 195 (12), 73 (8). HRMS (ESI(+)-TOF) *m/z*: $[M-OMe]^+$ Calcd for $C_{15}H_9F_2O^+$ 243.0621; Found 243.0623.

1,7-dimethoxy-3-phenyl-1H-isochromene[7] ***2j***

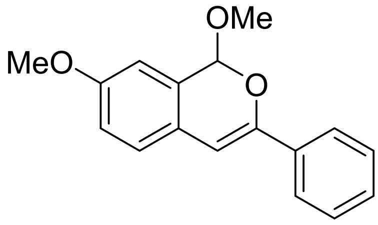

White solid, (57.6 mg, 0.22 mmol, 86% yield), m.p.: 92.0 – 93.0 °C. The reaction was purified through recrystallization using petroleum ether. ^1H NMR (400 MHz, $CDCl_3$): δ = 7.81-7.78 (m, 2H), 7.42-7.38 (m, 2H), 7.34-7.30 (m, 1H), 7.16 (d, *J* = 8.4 Hz, 1H), 6.93 (dd, *J* = 8.4, 2.6 Hz, 1H), 6.84 (d, *J* = 2.6 Hz, 1H), 6.58 (s, 1H), 6.10 (s, 1H), 3.84 (s, 3H), 3.60 (s, 3H). $^{13}C\{^1H\}$ NMR (100 MHz, $CDCl_3$): δ = 158.7, 147.5, 134.6, 128.4, 128.4, 128.3, 125.9, 124.5, 123.3, 115.8, 110.8, 100.1, 99.7, 55.5, 55.2. GC-MS (*m/z*, rel. int. %): 268 (M^+, 35), 237 (100), 221 (38), 207 (66), 165 (19), 73 (62).

3-(4-chlorophenyl)-1,7-dimethoxy-1H-isochromene ***2k***

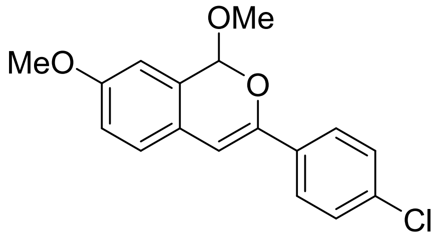

White solid, (62.2 mg, 0.20 mmol, 81% yield), m.p.: 95.0 – 96.0 °C. The reaction was purified through recrystallization using petroleum ether. ^1H NMR (400 MHz, $CDCl_3$): δ = 7.72-7.69 (m, 2H), 7.37-7.34 (m, 2H), 7.15 (d, *J* = 8.4 Hz, 1H), 6.93 (dd, *J* = 8.4, 2.6 Hz, 1H), 6.83 (d, *J* = 2.6 Hz, 1H), 6.55 (s, 1H), 6.08 (s, 1H), 3.84 (s, 3H), 3.58 (s, 3H). $^{13}C\{^1H\}$ NMR (100 MHz, $CDCl_3$): δ = 158.9, 146.4, 134.0, 133.1, 128.6, 128.4, 126.0, 125.7, 122.9, 115.9, 110.8, 100.5, 99.8, 55.5, 55.2. GC-MS (*m/z*, rel. int. %): 302 (M^+, 0), 272 (100), 257 (25), 209 (55), 194 (27), 165 (33). HRMS (ESI(+)-TOF) *m/z*: $[M-OMe]^+$ Calcd for $C_{16}H_{12}ClO_2^+$ 271.0526; Found 271.0523.

5-methoxy-7-phenyl-5H-pyrano[4,3-b]pyridine ***2l***

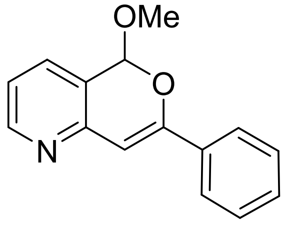

Yellow oil, (50.2 mg, 0.21 mmol, 84% yield). The reaction was purified through column chromatography on silica gel flash (elution: toluene). ^{1}H NMR (400 MHz, $CDCl_3$): δ = 8.47 (d, *J* = 3.3 Hz, 1H), 7.78 – 7.76 (m, 2H), 7.52 (d, *J* = 9.0 Hz, 1H), 7.38 – 7.30 (m, 3H), 7.09 (dd, *J* = 4.9, 7.6 Hz, 1H), 6.80 (s, 1H), 6.14 (s, 1H), 3.55 (s, 3H). ^{13}C{^{1}H} NMR (100 MHz, $CDCl_3$): δ = 153.8, 148.6, 148.3, 133.2, 132.5, 128.9, 127.6, 124.4, 121.6, 120.3, 99.7, 98.7, 54.5. GC-MS (*m/z*, rel. int. %): 239 (M^+, 28), 209 (22), 208 (100), 180 (26), 152 (10), 77 (16). HRMS (ESI(+)-TOF) *m/z*: $[M+H]^+$ Calcd for $C_{15}H_{14}NO_2^+$ 240,1019; Found 240,1024.

2-(1-methoxy-1H-isochromen-3-yl)propan-2-ol ***2m***

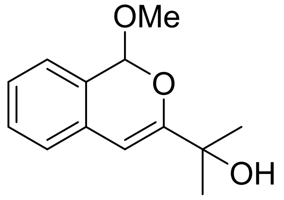

Colorless oil, (50.6 mg, 0.23 mmol, 92% yield). The reaction was purified through column chromatography on silica gel flash (elution: toluene). ^{1}H NMR (400 MHz, DMSO-d_6): δ = 7.16 (td, *J* = 1.6, 7.3 Hz, 1H), 7.10 – 7.08 (m, 1H), 7.04 (td, *J* = 1.2, 7.3 Hz, 1H), 6.98 (dd, *J* = 1.2, 7.6 Hz, 1H), 5.99 (s, 1H), 5.90 (s, 1H), 4.93 (s, 1H), 3.29 (s, 3H), 1.22 (s, 3H), 1.19 (s, 3H). ^{13}C{^{1}H} NMR (100 MHz, DMSO-d_6): δ = 160.0, 130.6, 129.6, 126.8, 126.5, 126.2, 124.1, 99.4, 96.9, 70.3, 55.2, 29.3, 28.5. HRMS (ESI(+)-TOF) *m/z*: $[M-OMe]^+$ Calcd for $C_{12}H_{13}O_2^+$ 189.0916; Found 189.0919.

8.2 Compounds 3a-3e

(Z)-1-benzylidene-3-methoxy-1,3-dihydroisobenzofuran[9] ***3a***

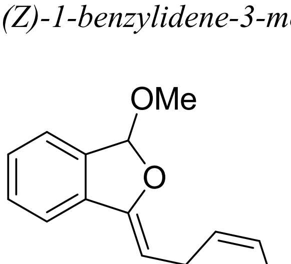

Yellow oil, (52.9 mg, 0.22 mmol, 89% yield). ^{1}H NMR (400 MHz, DMSO-d_6): δ = 7.81 – 7.75 (m, 3H), 7.55 – 7.50 (m, 2H), 7.48 – 7.44 (m, 1H), 7.37 (t, *J* = 7.7 Hz, 2H), 7.19 (t, *J* = 7.4 Hz, 1H), 6.66 (s, 1H), 6.25 (s, 1H), 3.48 (s, 3H). ^{13}C{^{1}H} NMR (100 MHz, DMSO-d_6): δ = 153.2, 137.4, 136.2, 135.3, 130.6, 129.7, 128.9, 128.5, 126.3, 123.9, 120.5, 107.9, 98.2, 55.1. GC-MS (*m/z*, rel. int. %): 238 (M^+, 100), 207 (52), 206 (56), 179 (92), 178 (91), 89 (28).

(Z)-1-(4-chlorobenzylidene)-3-methoxy-1,3-dihydroisobenzofuran[1] ***3b***

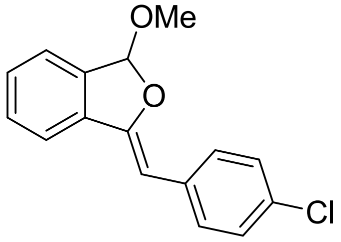

White solid, (54.4 mg, 0.20 mmol, 80% yield), m.p.: 72.2 – 73.5 °C. The reaction was purified through recrystallization using petroleum ether. ^{1}H NMR (400 MHz, DMSO-d_6): δ = 7.82 (d, *J* = 7.8 Hz, 1H), 7.78 (d, *J* = 8.6 Hz, 2H), 7.59 – 7.55 (m, 2H), 7.52 – 7.48 (m, 1H), 7.45 (d, *J* = 8.6 Hz, 2H), 6.71 (s, 1H), 6.30 (s, 1H), 3.50 (s, 3H). ^{13}C{^{1}H} NMR (100 MHz, DMSO-d_6): δ = 153.8, 137.5, 135.2, 135.0, 130.6, 130.4, 130.0, 129.9, 128.9, 123.9, 120.6, 108.1, 97.0, 55.1. GC-MS (*m/z*, rel. int. %): 274 (8), 272 (M$^+$, 23), 241 (13), 240 (9), 178 (19), 44 (100).

(Z)-1-(4-fluorobenzylidene)-3-methoxy-1,3-dihydroisobenzofuran ***3c***

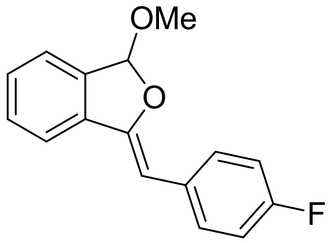

Yellow oil, (53.8 mg, 0.21 mmol, 84% yield). ^{1}H NMR (400 MHz, DMSO-d_6): δ = 7.80 – 7.75 (m, 3H), 7.52 – 7.49 (m, 2H), 7.46 – 7.42 (m, 1H), 7.19 (d, *J* = 8.9 Hz, 2H), 6.64 (s, 1H), 6.25 (s, 1H), 3.46 (s, 3H). ^{13}C{^{1}H} NMR (100 MHz, DMSO-d_6): δ = 160.8 (d, *J* = 243.9 Hz), 152.9 (d, *J* = 2.9 Hz), 137.1, 135.2, 132.7 (d, *J* = 3.1 Hz), 130.6, 130.2 (d, *J* = 7.7 Hz), 129.7, 123.9, 120.4, 115.8 (d, *J* = 21.2 Hz), 107.9, 97.1, 55.0. ^{19}F NMR (377 MHz, $CDCl_3$): δ = 115.8. GC-MS (*m/z*, rel. int. %): 256 (M$^+$, 100), 224 (53), 223 (50), 196 (67), 177 (19), 98 (14). HRMS (ESI(+)-TOF) *m/z*: [M+H]$^+$ Calcd for $C_{16}H_{13}FO_2^+$ 257.0972; Found 257.0972.

(Z)-3-(4-chlorobenzylidene)-5-fluoro-1-methoxy-1,3-dihydroisobenzofuran ***3d***

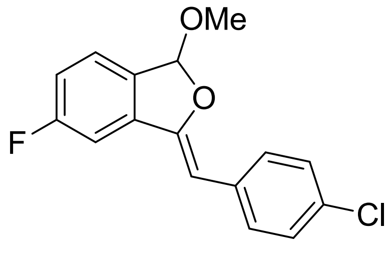

White solid, (63.1 mg, 0.22 mmol, 87% yield), m.p.: 103.6 – 104.8 °C. The reaction was purified through recrystallization using petroleum ether. ^{1}H NMR (400 MHz, DMSO-d_6): δ = 7.73 – 7.68 (m, 3H), 7.56 (dd, *J* = 4.9, 8.3 Hz, 2H), 7.45 – 7.41 (m, 2H), 7.32 – 7.27 (m, 1H), 6.65 (s, 1H), 6.34 (s, 1H), 3.47 (s, 3H). ^{13}C{^{1}H} NMR (100 MHz, DMSO-d_6): δ = 164.0 (d, *J* = 245.0 Hz), 152.8 (d, *J* = 3.6 Hz), 137.6 (d, *J* = 10.4 Hz), 134.8, 133.6, 130.8, 130.1, 129.0, 126.1 (d, *J* = 9.9 Hz), 117.4 (d, *J* = 24.2 Hz), 107.7, 107.4 (d, *J* = 24.8 Hz), 98.2, 55.2. ^{19}F NMR (377 MHz, $CDCl_3$): δ = 111.7. GC-MS (*m/z*, rel. int. %): 290 (M$^+$, 60), 259 (32), 223 (21), 207 (33), 196 (42), 44 (100). HRMS (ESI(+)-TOF) *m/z*: [M-OMe]$^+$ Calcd for $C_{15}H_9ClFO^+$ 259,0326; Found 259,0327.

(Z)-1-methoxy-3-(4-methylbenzylidene)-1,3-dihydroisobenzofuran[9] ***3e***

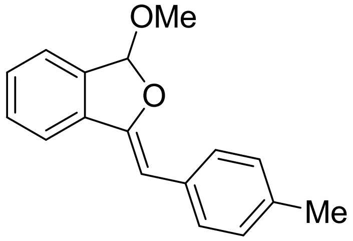

Yellow oil, (53.5 mg, 0.21 mmol, 42% yield). ^{1}H NMR (400 MHz, DMSO-d_6): δ = 7.82 (d, J = 8.6 Hz, 1H), 7.69 (d, J = 8.2 Hz, 2H), 7.60 – 7.55 (m, 2H), 7.50 (dd, J = 1.0, 7.2 Hz, 1H), 7.23 (d, J = 7.9 Hz, 2H), 6.70 (s, 1H), 6.25 (s, 1H), 3.51 (s, 3H), 2.35 (s, 3H). ^{13}C{^{1}H} NMR (100 MHz, DMSO-d_6): δ = 152.5, 137.2, 135.5, 135.4, 133.3, 130.6, 129.6, 129.5, 128.5, 123.8, 120.4, 107.7, 98.2, 55.0, 21.3. GC-MS (*m/z*, rel. int. %): 252 (M^+, 100), 221 (36), 220 (37), 193 (47), 178 (52), 165 (17).

9. NMR SPECTRA

9.1 Compounds 2a-2m

^{1}H NMR (400 MHz, $CDCl_3$) of compound **2a**.

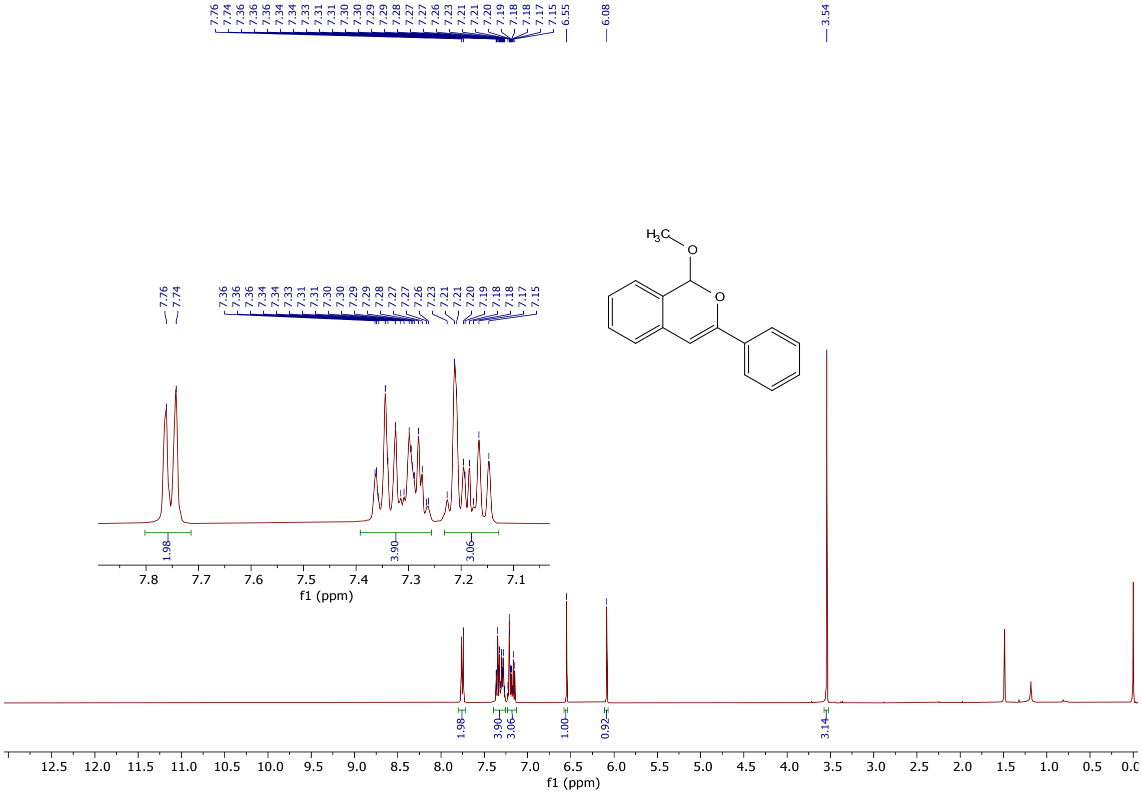

$^{13}C\{^1H\}$ NMR (100 MHz, $CDCl_3$) of compound **2a**.

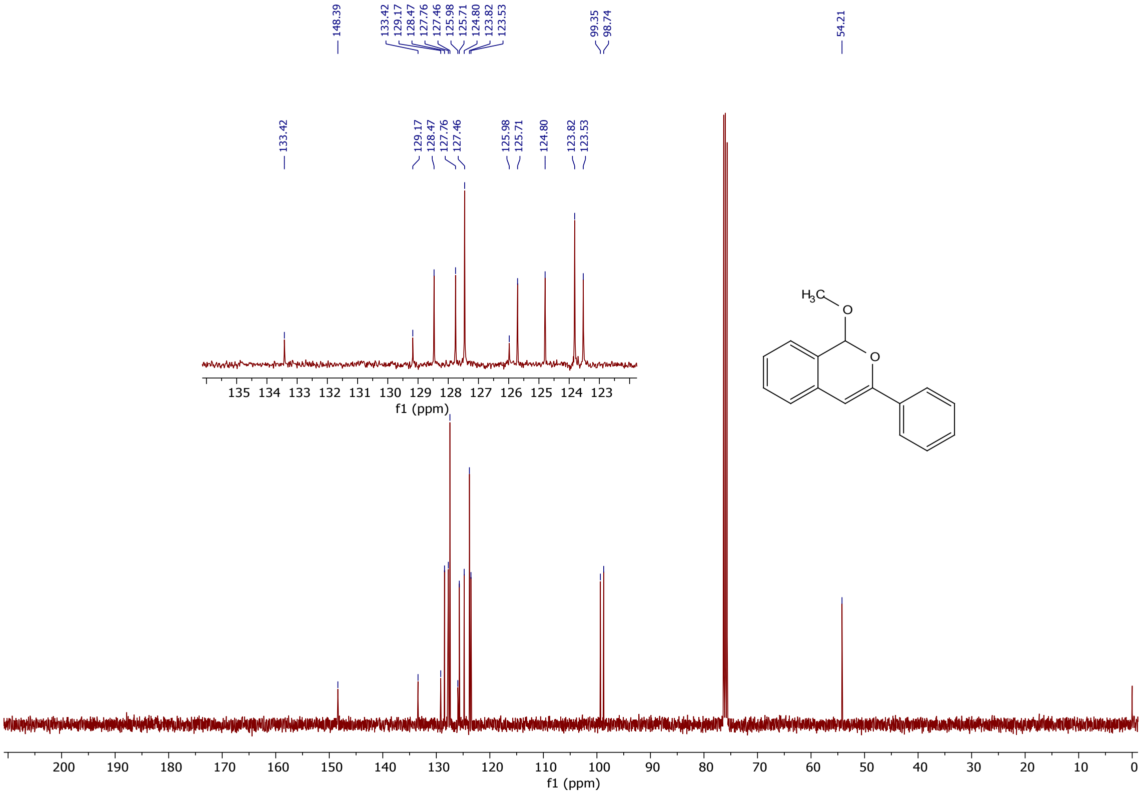

^{1}H NMR (400 MHz, $CDCl_3$) of compound **2b**.

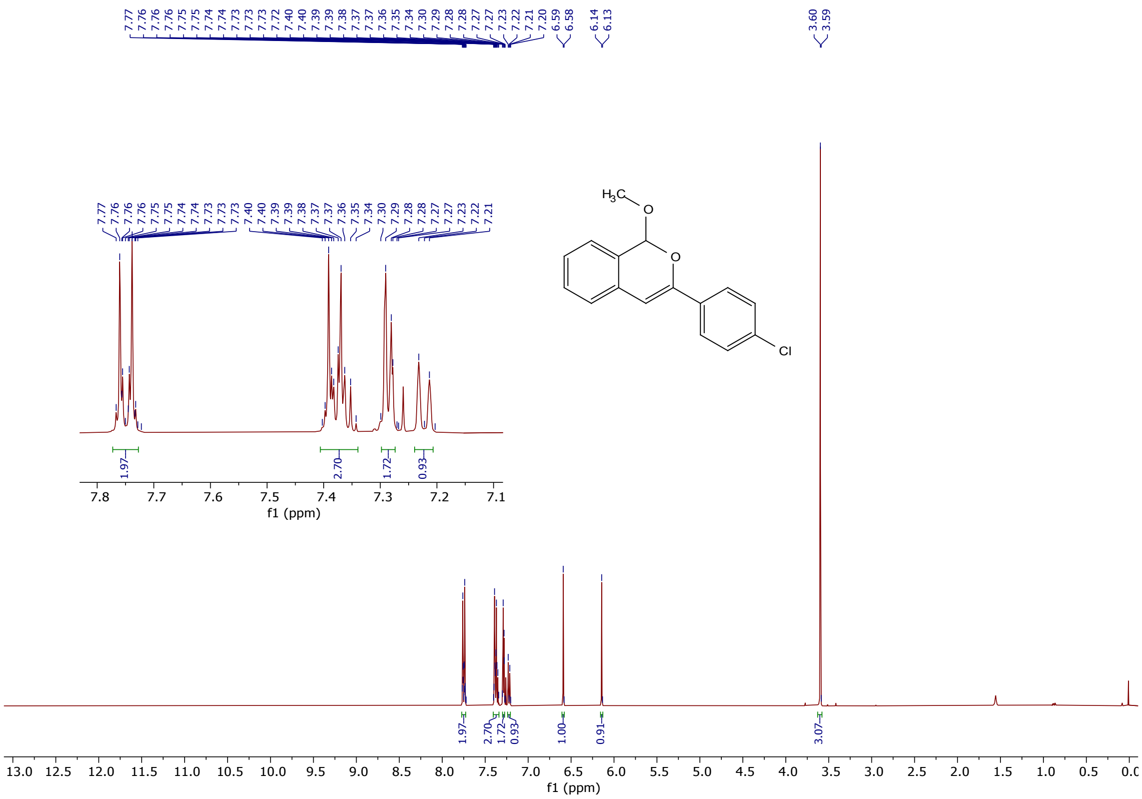

^{13}C{^{1}H} NMR (100 MHz, $CDCl_3$) of compound **2b**.

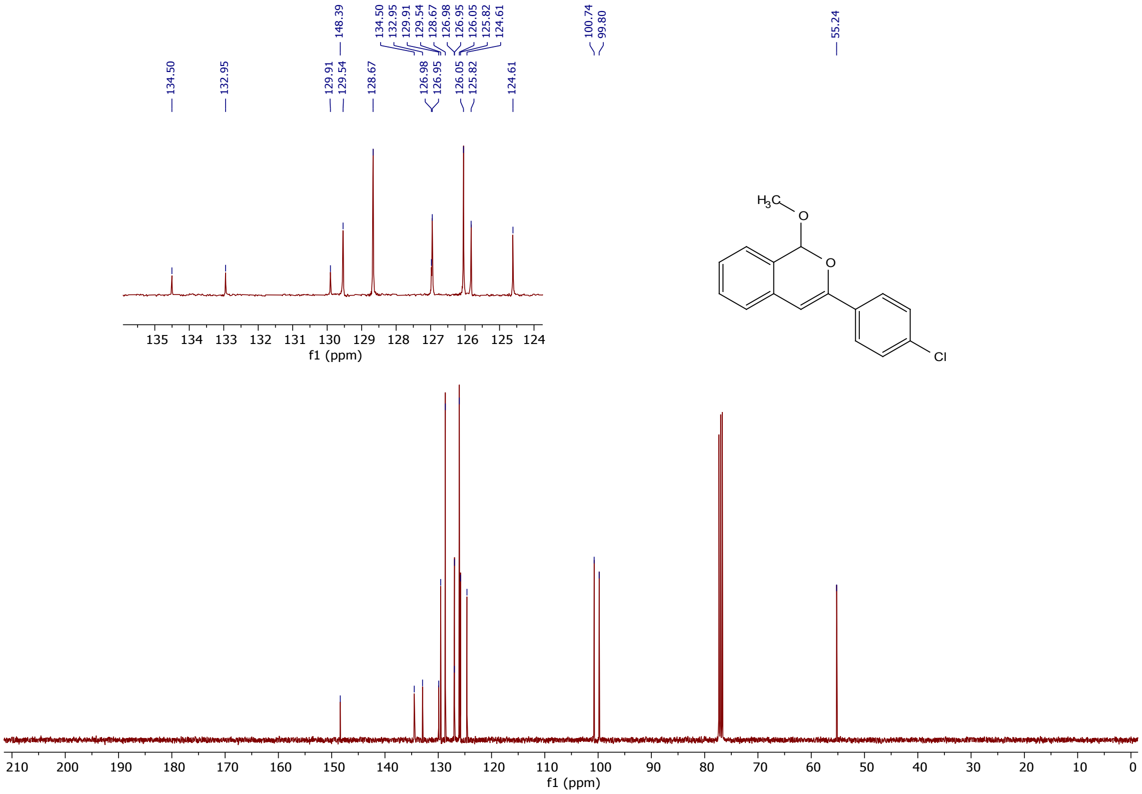

^{1}H NMR (400 MHz, $CDCl_3$) of compound **2c**.

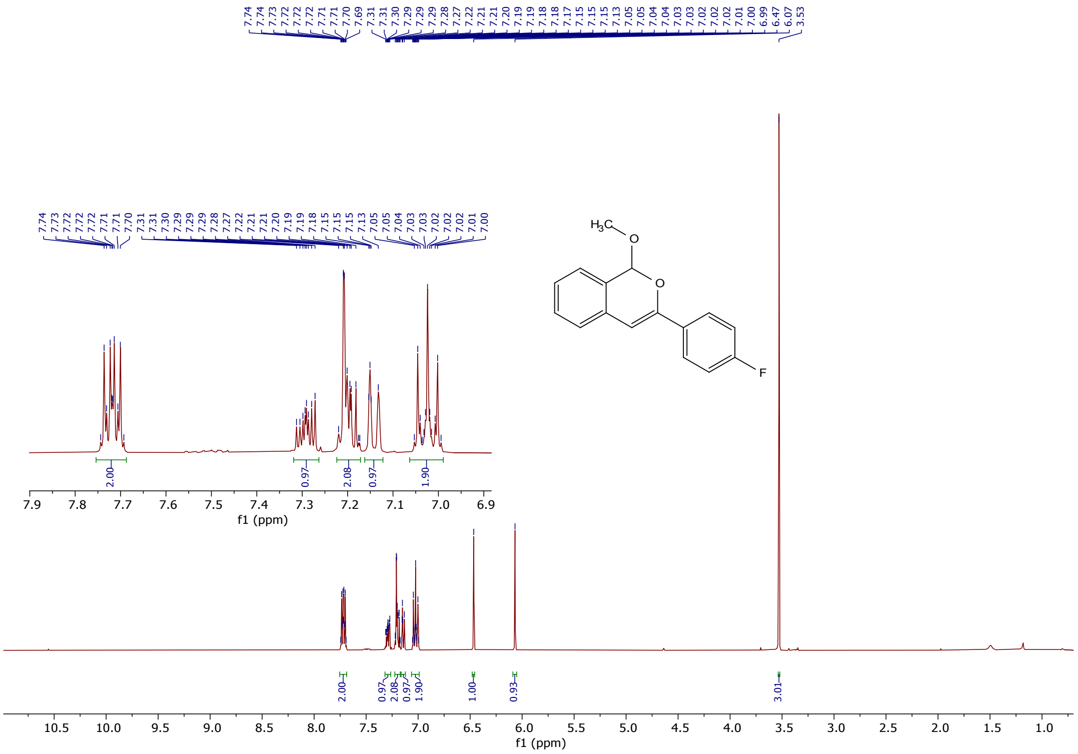

^{13}C{^{1}H} NMR (100 MHz, $CDCl_3$) of compound **2c**.

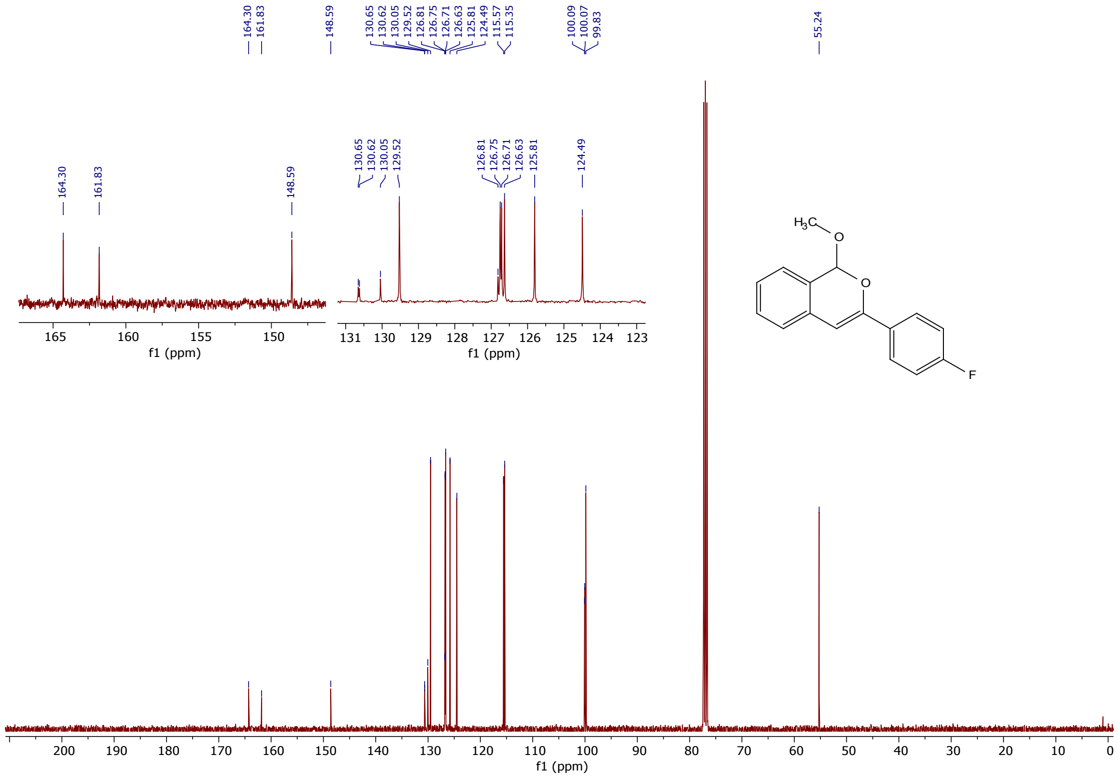

^{19}F NMR (377 MHz, $CDCl_3$) of compound **2c**.

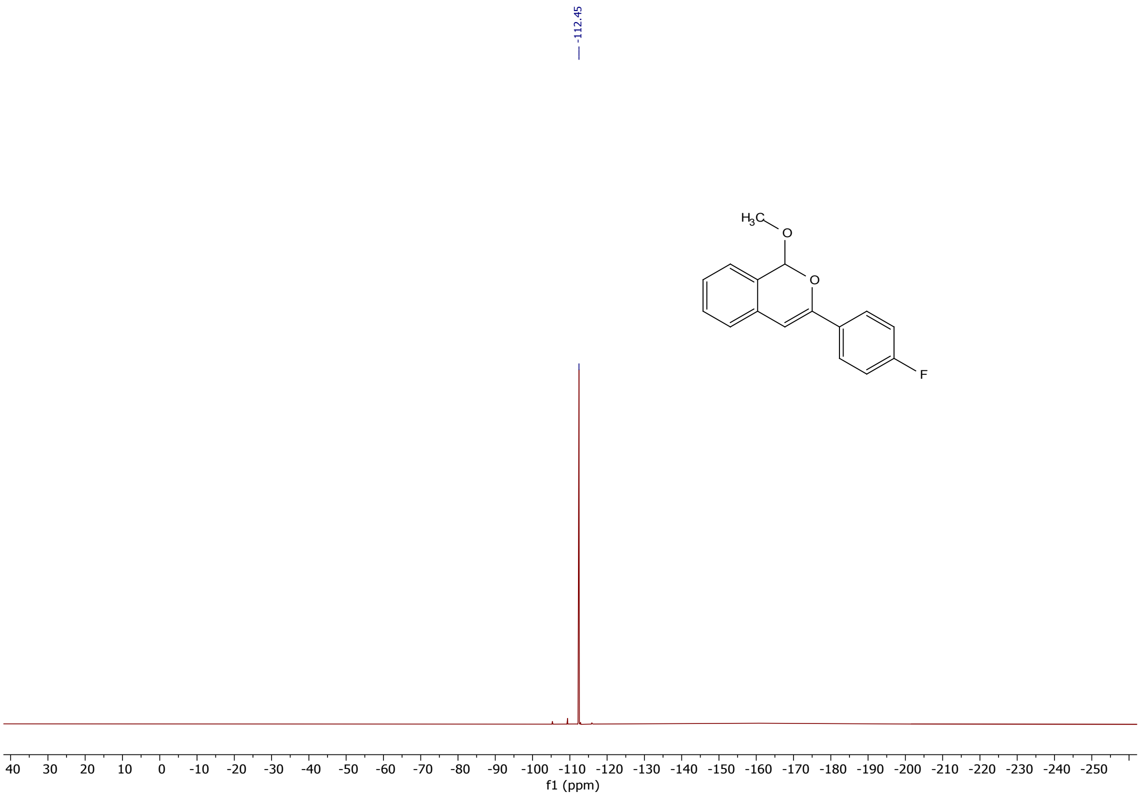

^{1}H NMR (400 MHz, $CDCl_3$) of compound **2d**.

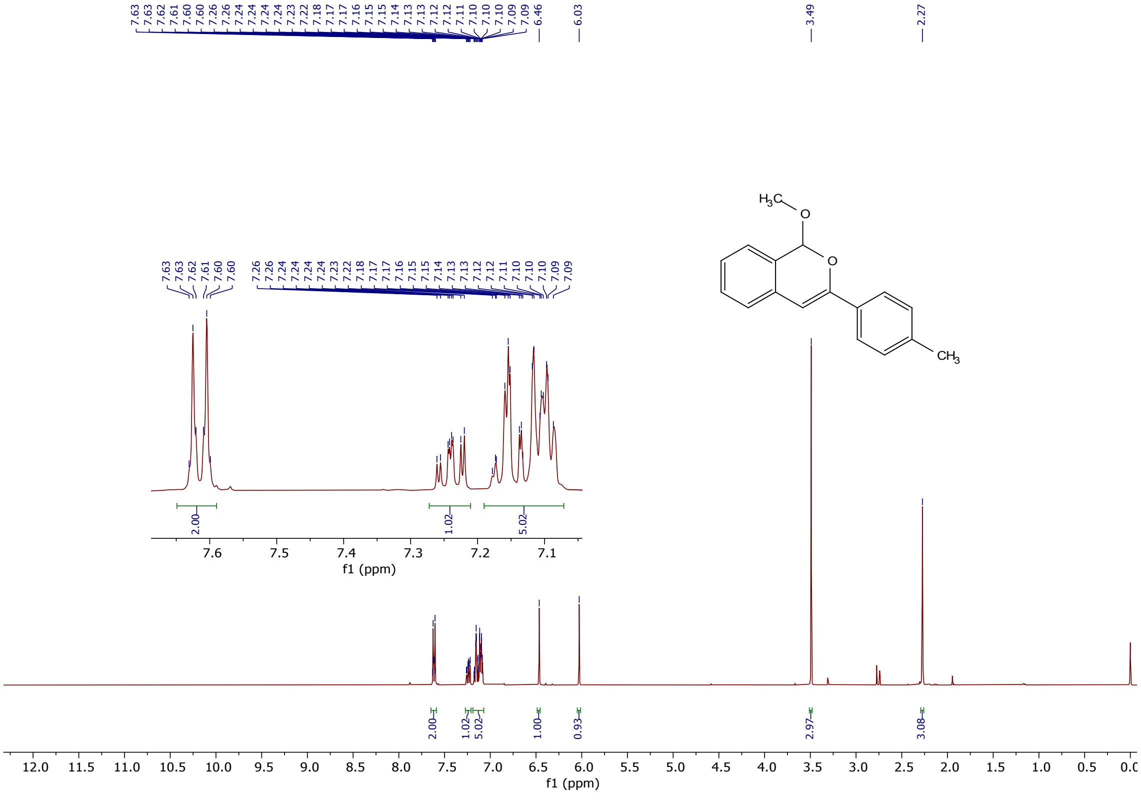

$^{13}C\{^1H\}$ NMR (100 MHz, $CDCl_3$) of compound **2d**.

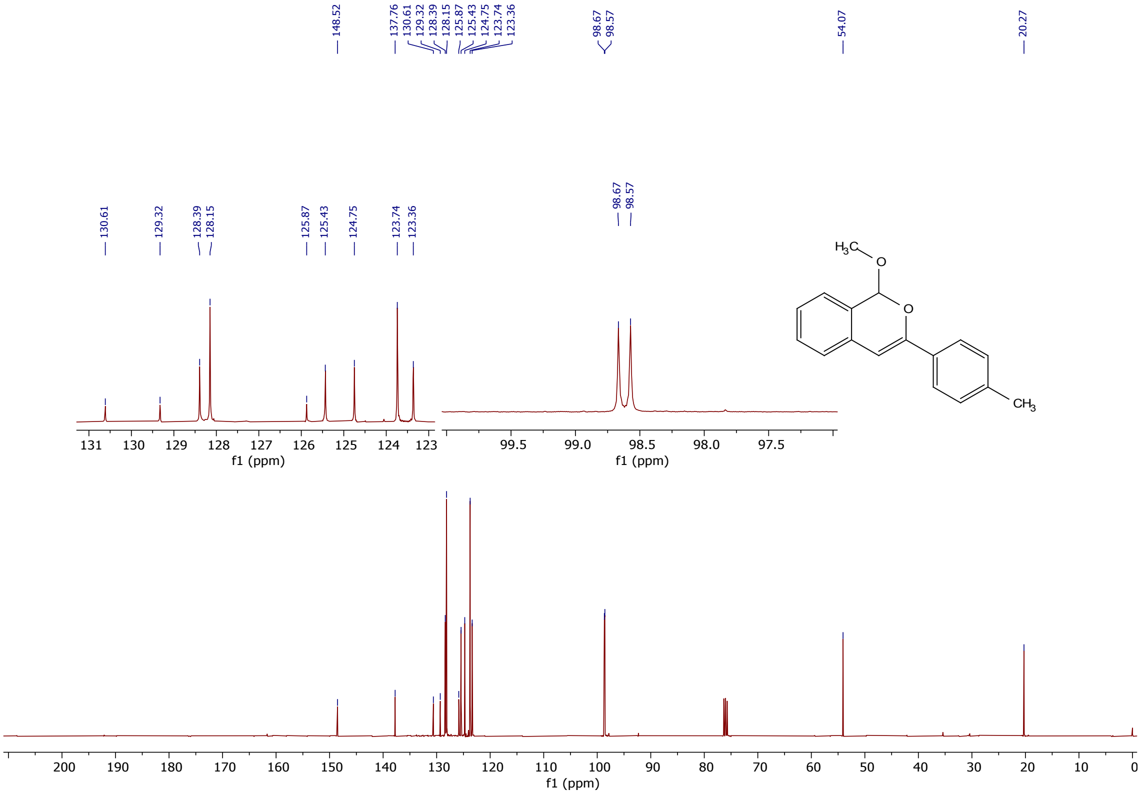

^1H NMR (400 MHz, DMSO-*d6*) of compound **2e**.

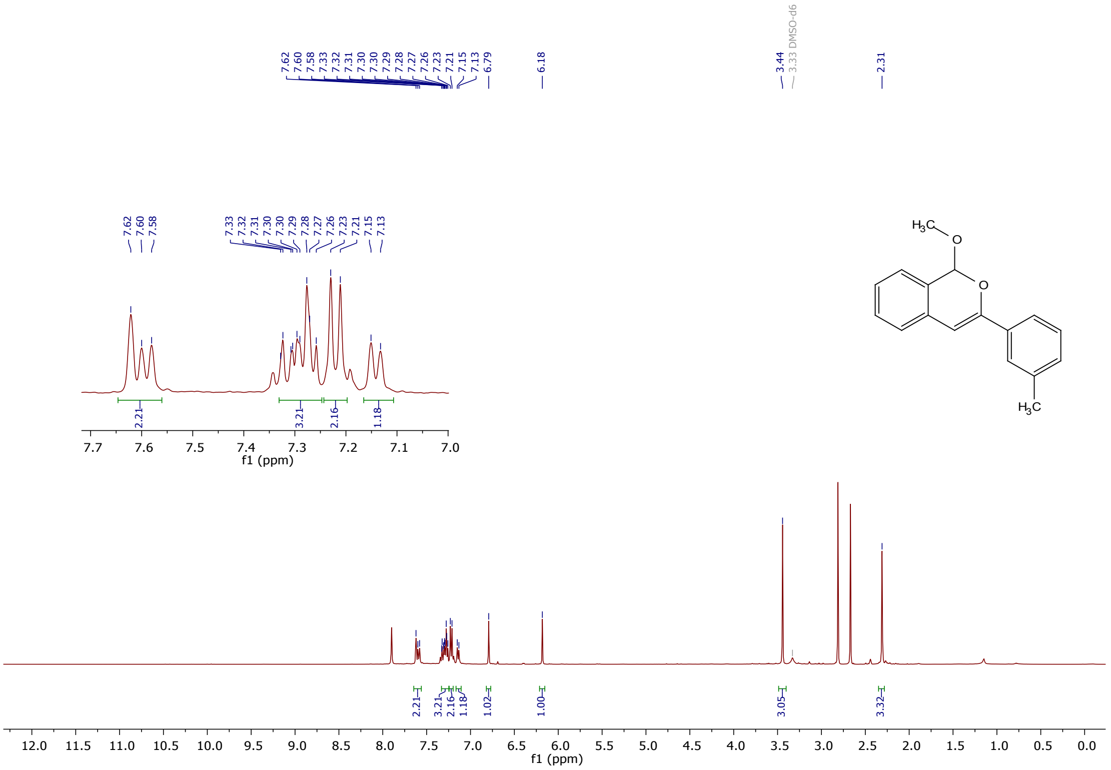

$^{13}C\{^{1}H\}$ NMR (100 MHz, DMSO-*d6*) of compound **2e**.

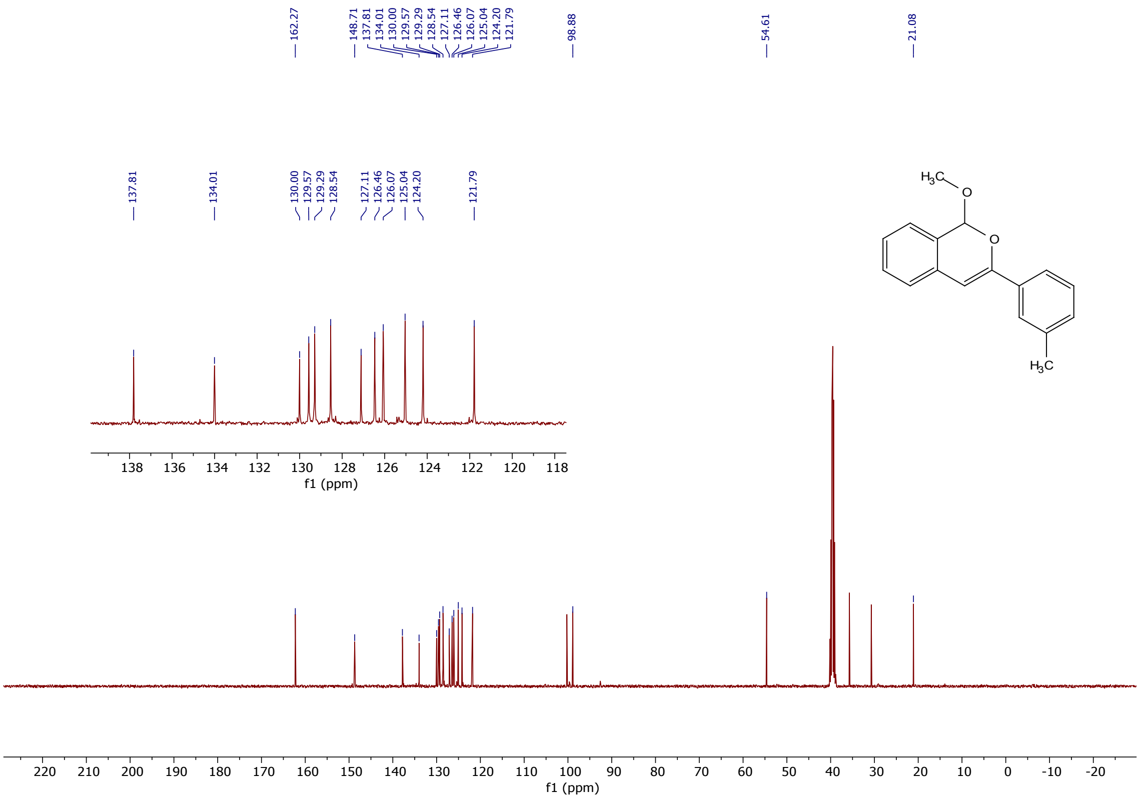

^{1}H NMR (400 MHz, $CDCl_3$) of compound **2f**.

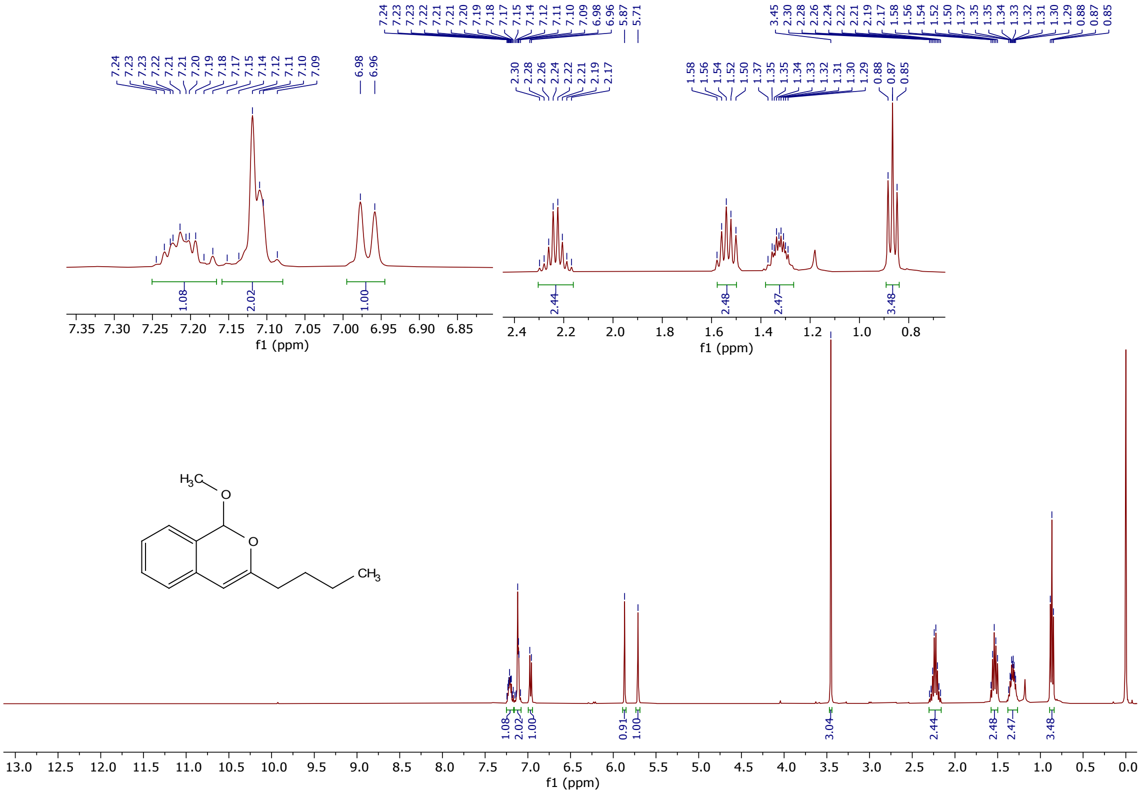

$^{13}C\{^1H\}$ NMR (100 MHz, $CDCl_3$) of compound **2f**.

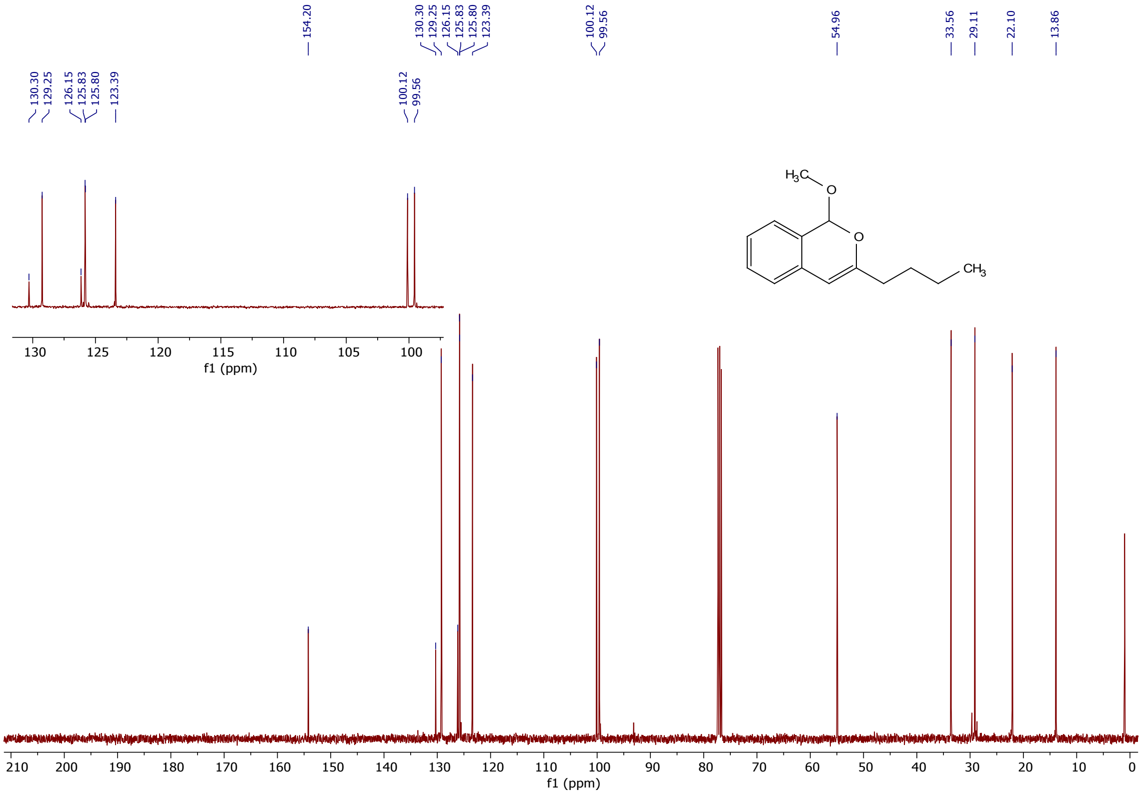

^1H NMR (400 MHz, $CDCl_3$) of compound **2g**.

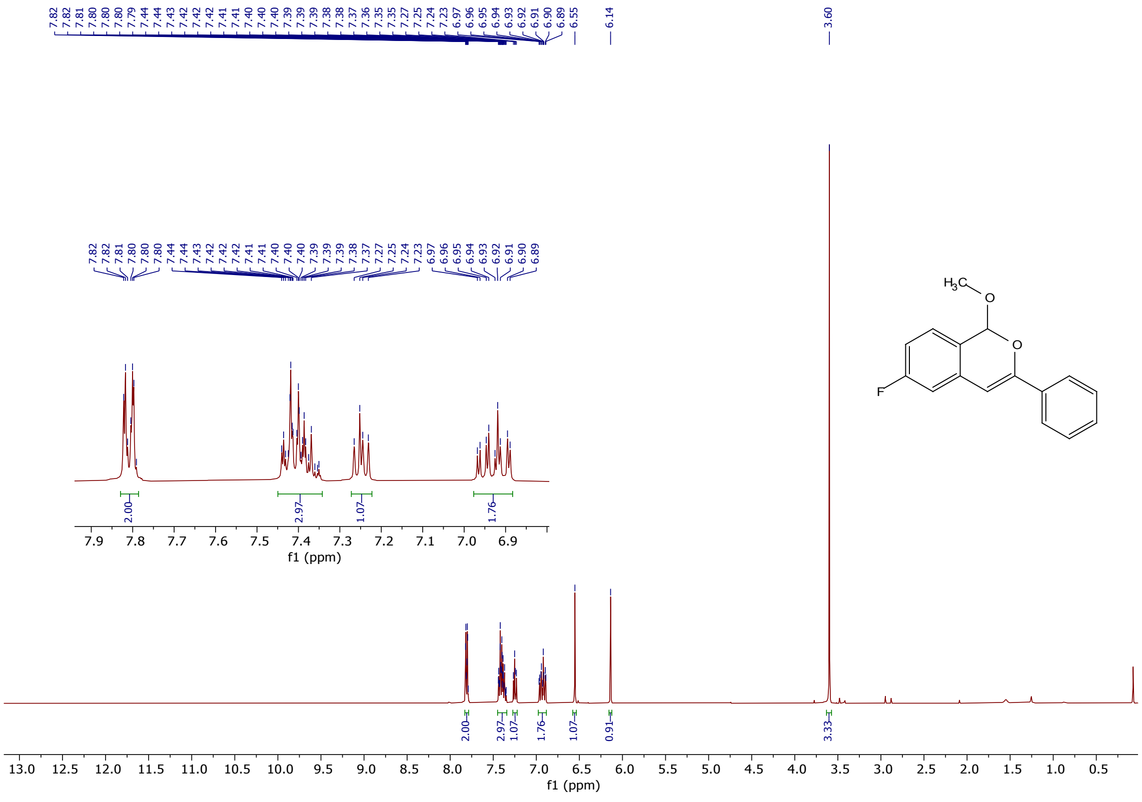

$^{13}C\{^1H\}$ NMR (100 MHz, $CDCl_3$) of compound **2g**.

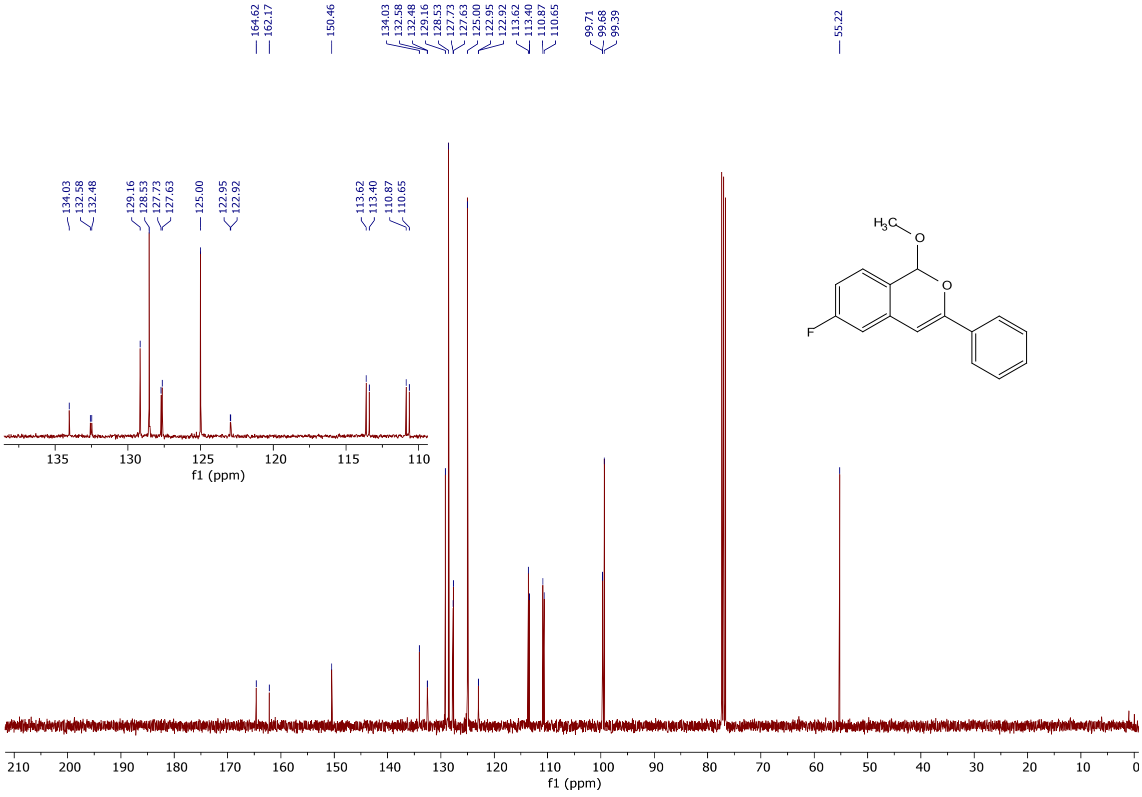

^{19}F NMR (377 MHz, $CDCl_3$) of compound **2g**.

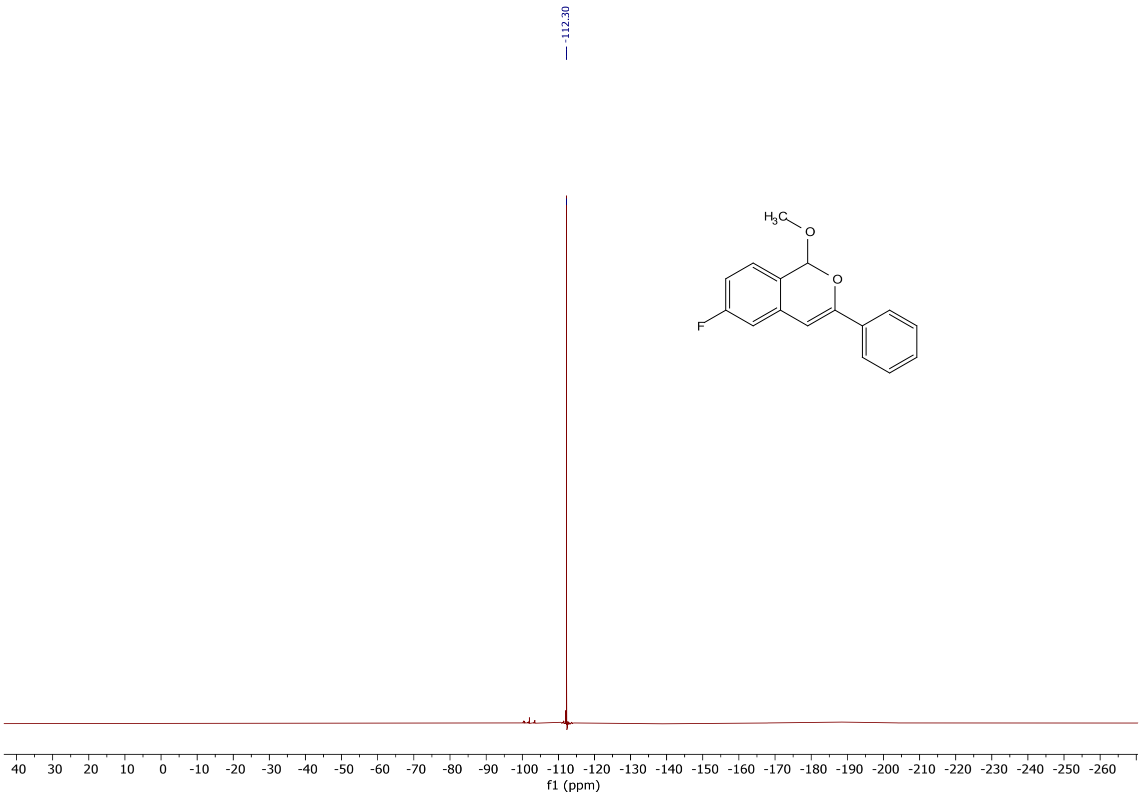

^{1}H NMR (400 MHz, $CDCl_3$) of compound **2h**.

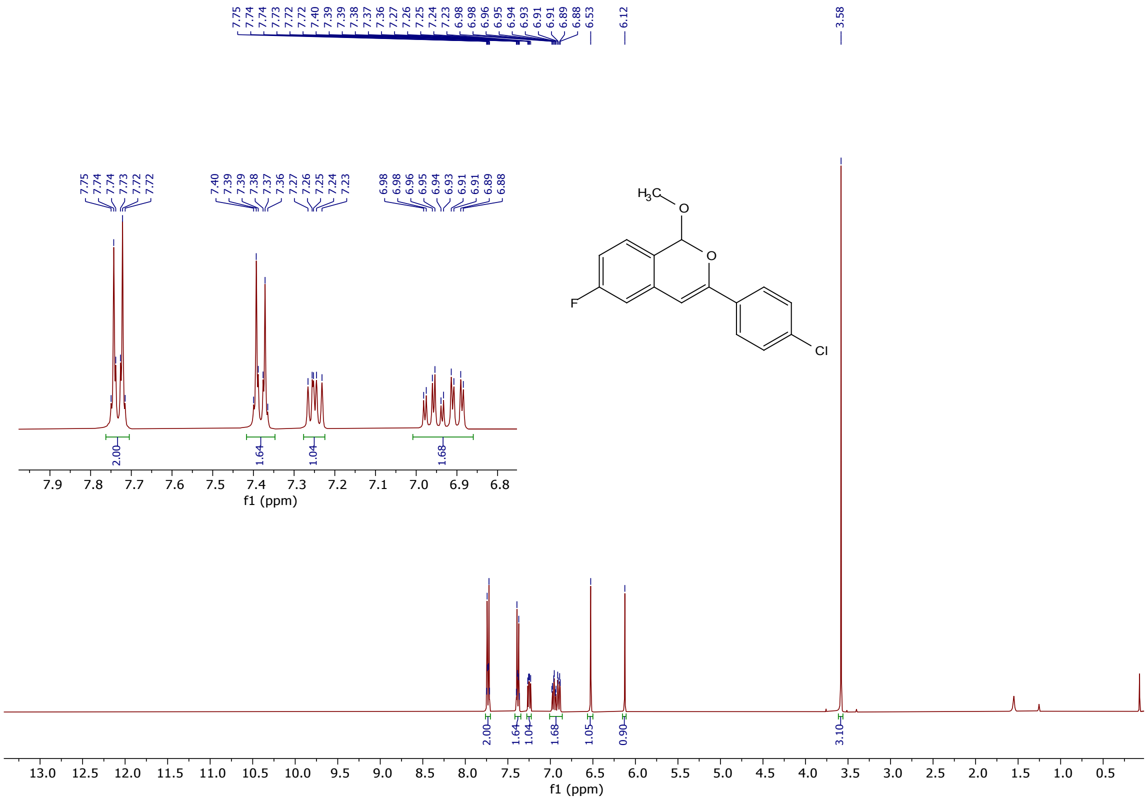

^{13}C{^{1}H} NMR (100 MHz, $CDCl_3$) of compound **2h**.

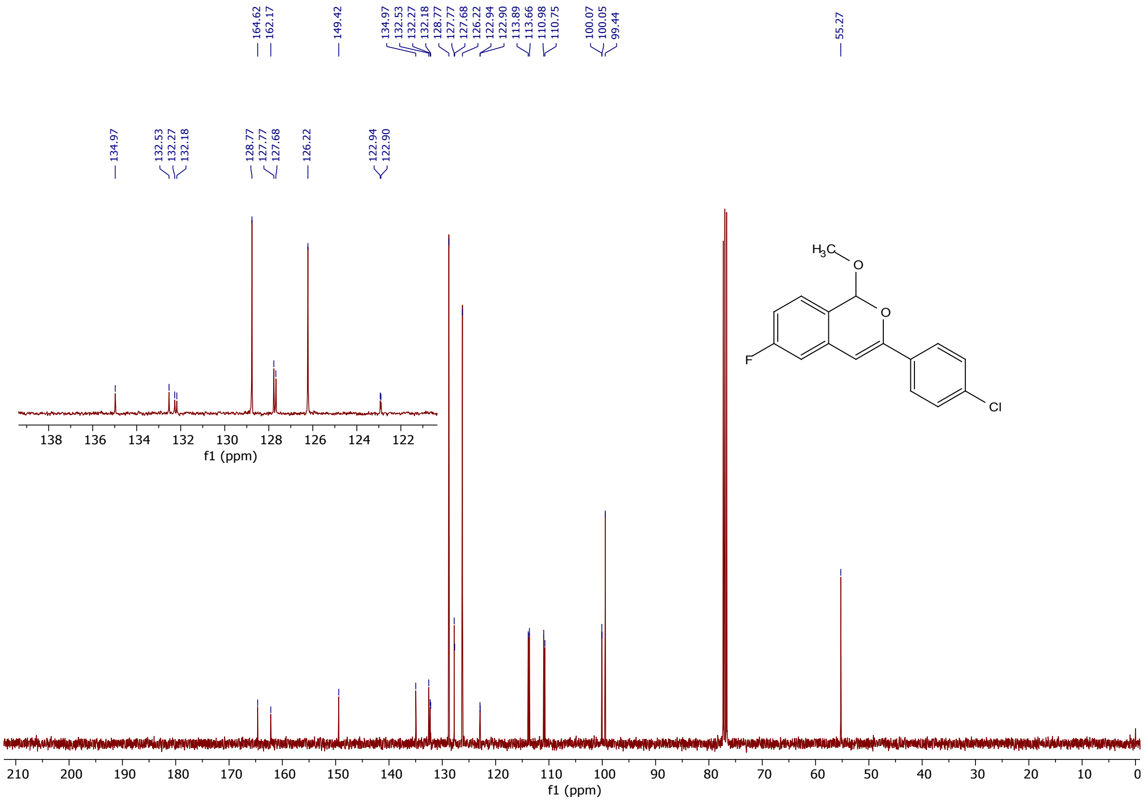

^{19}F NMR (377 MHz, $CDCl_3$) of compound **2h**.

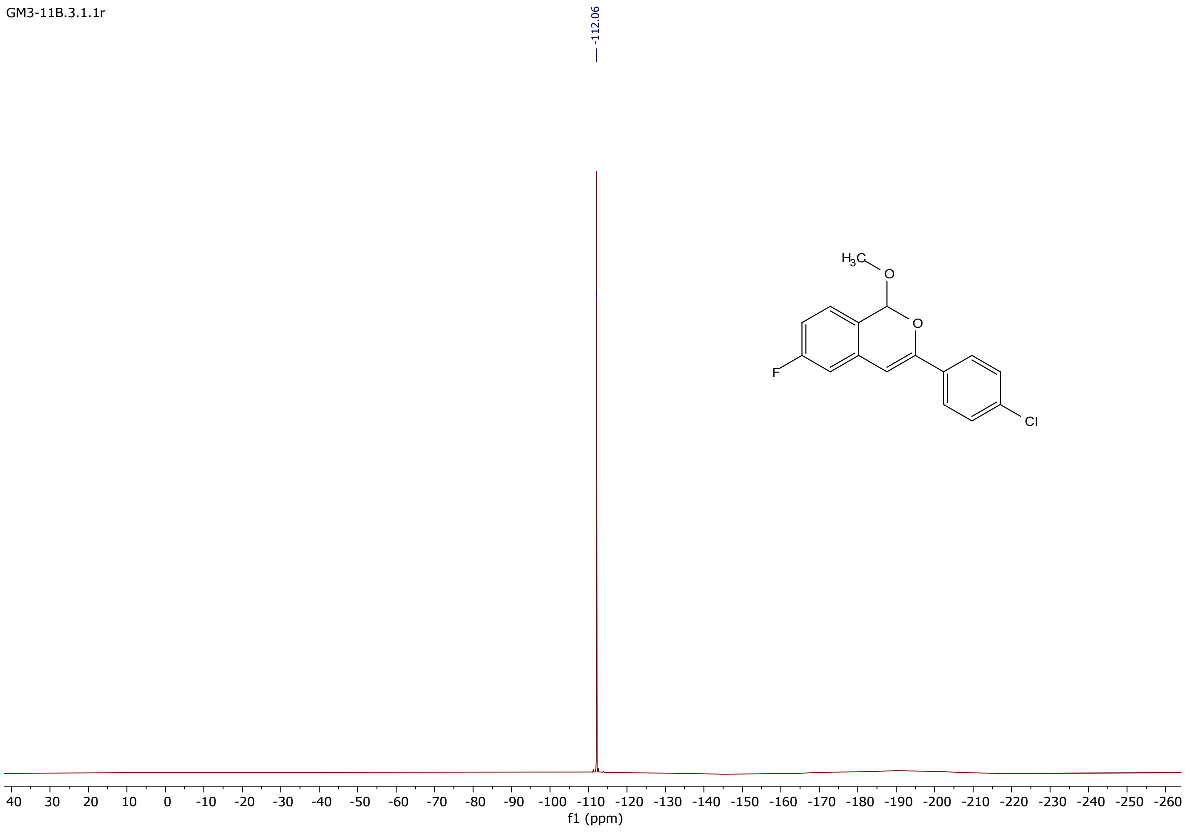

^{1}H NMR (400 MHz, $CDCl_3$) of compound **2i**.

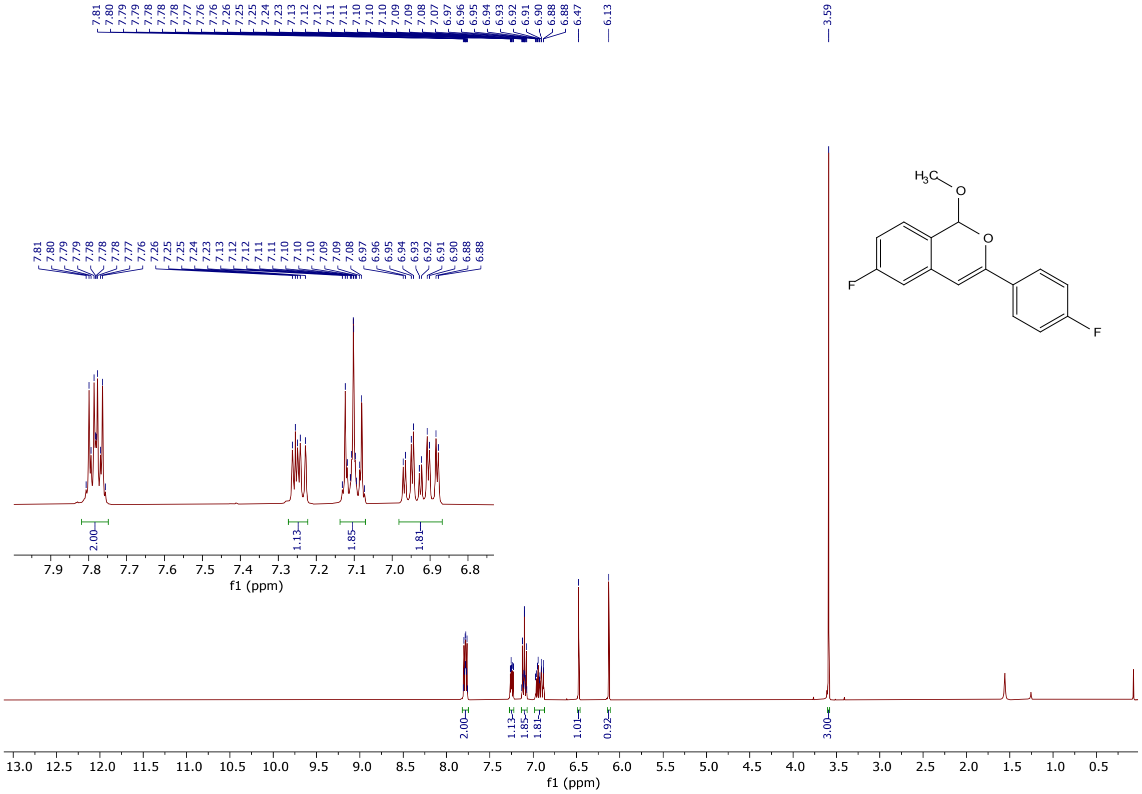

$^{13}C\{^{1}H\}$ NMR (100 MHz, $CDCl_3$) of compound **2i**.

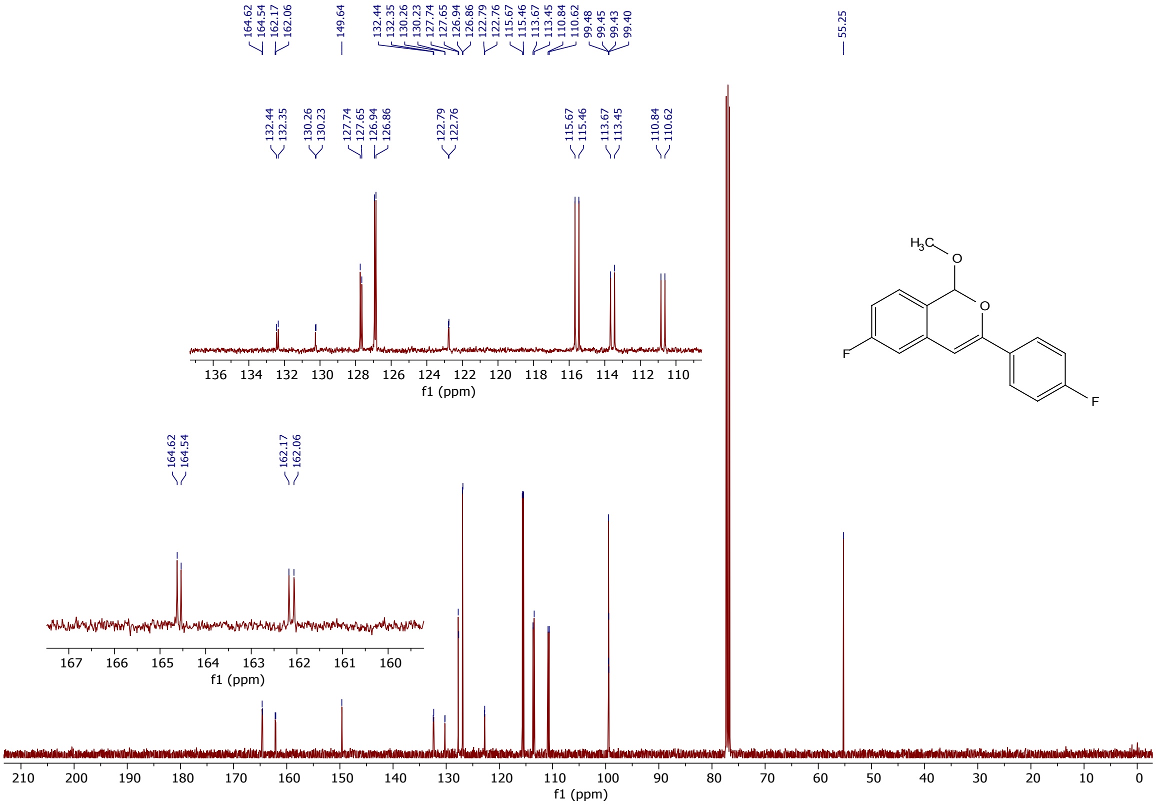

^{19}F NMR (377 MHz, $CDCl_3$) of compound **2i**.

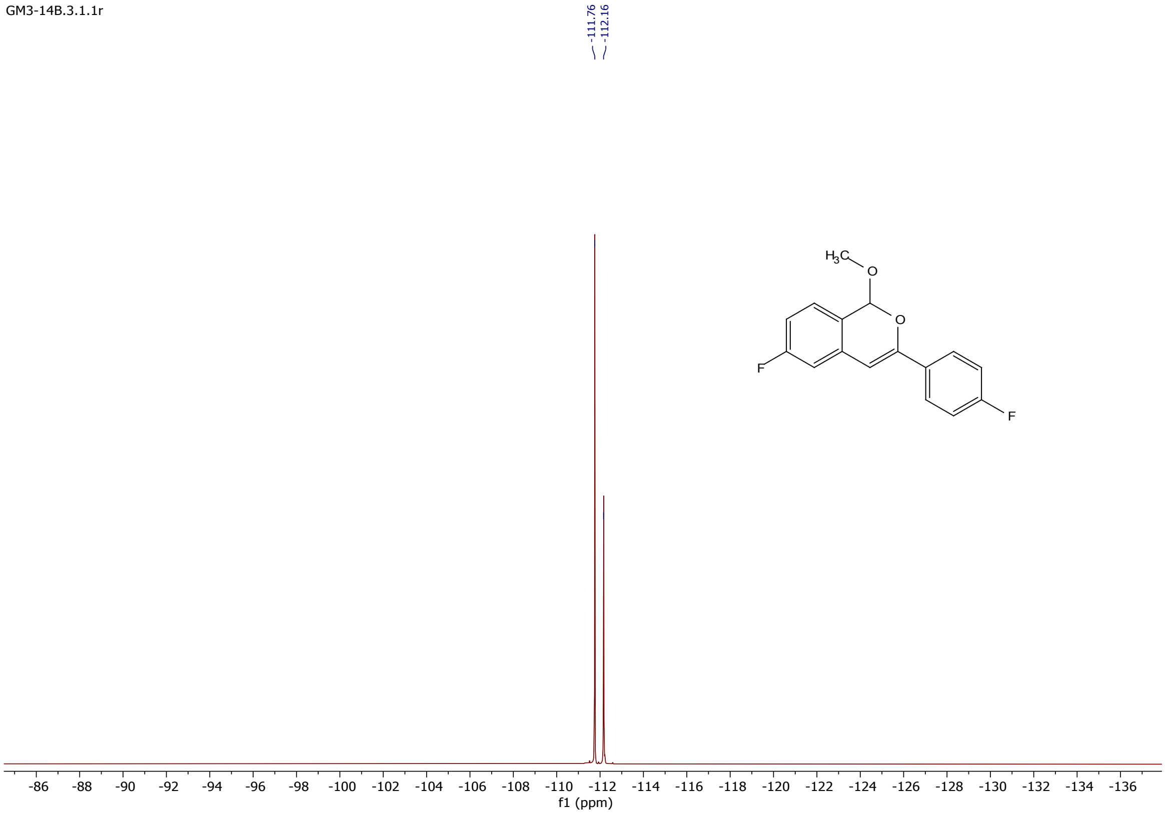

^{1}H NMR (400 MHz, $CDCl_3$) of compound **2j**.

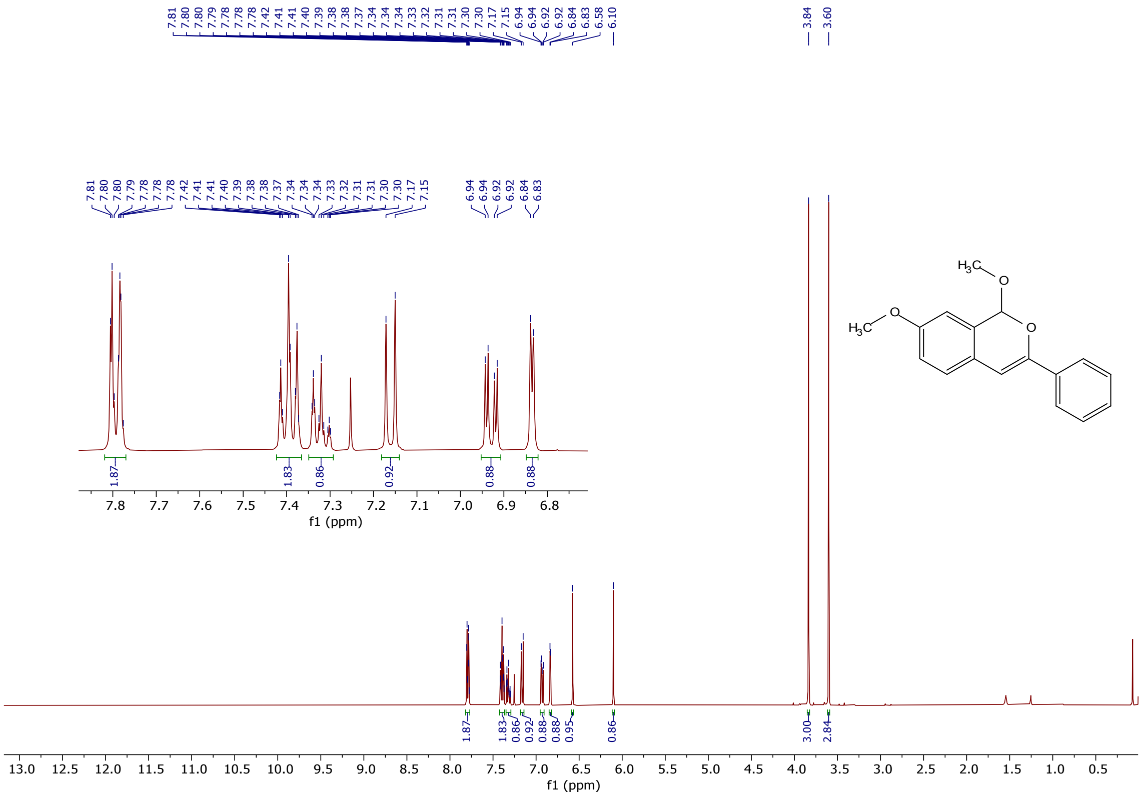

$^{13}C\{^{1}H\}$ NMR (100 MHz, $CDCl_3$) of compound **2j**.

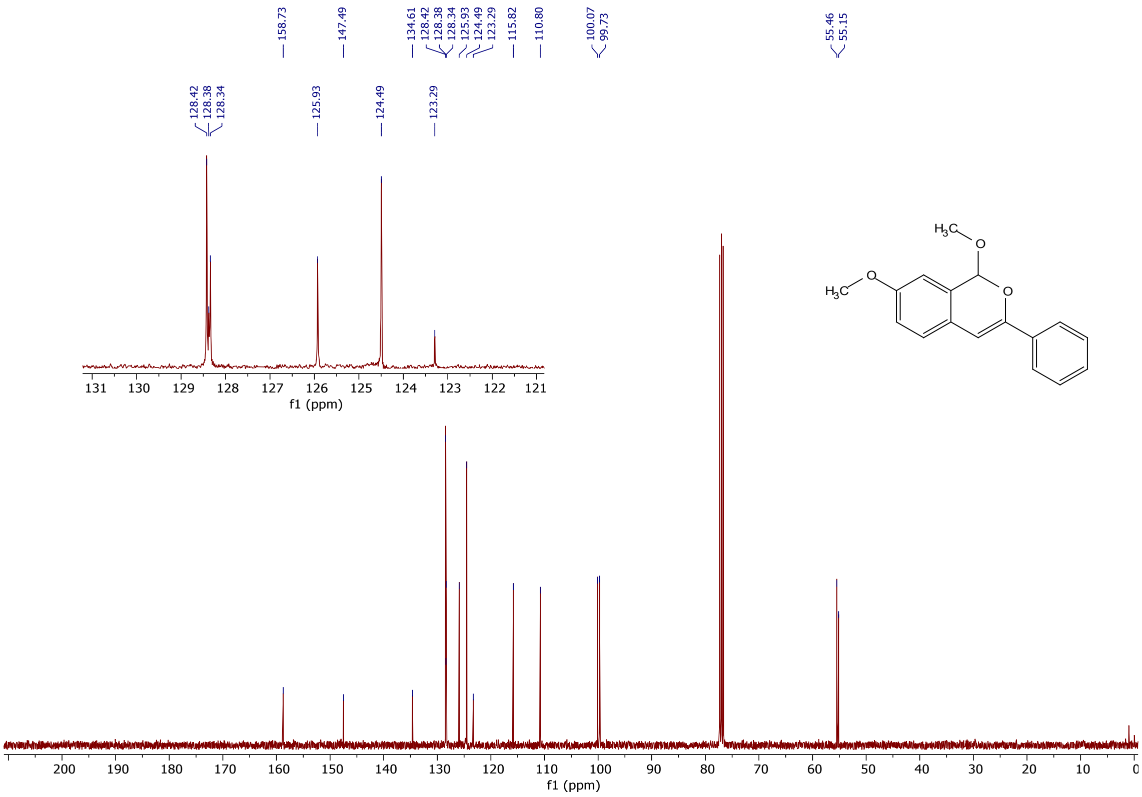

^{1}H NMR (400 MHz, $CDCl_3$) of compound **2k**.

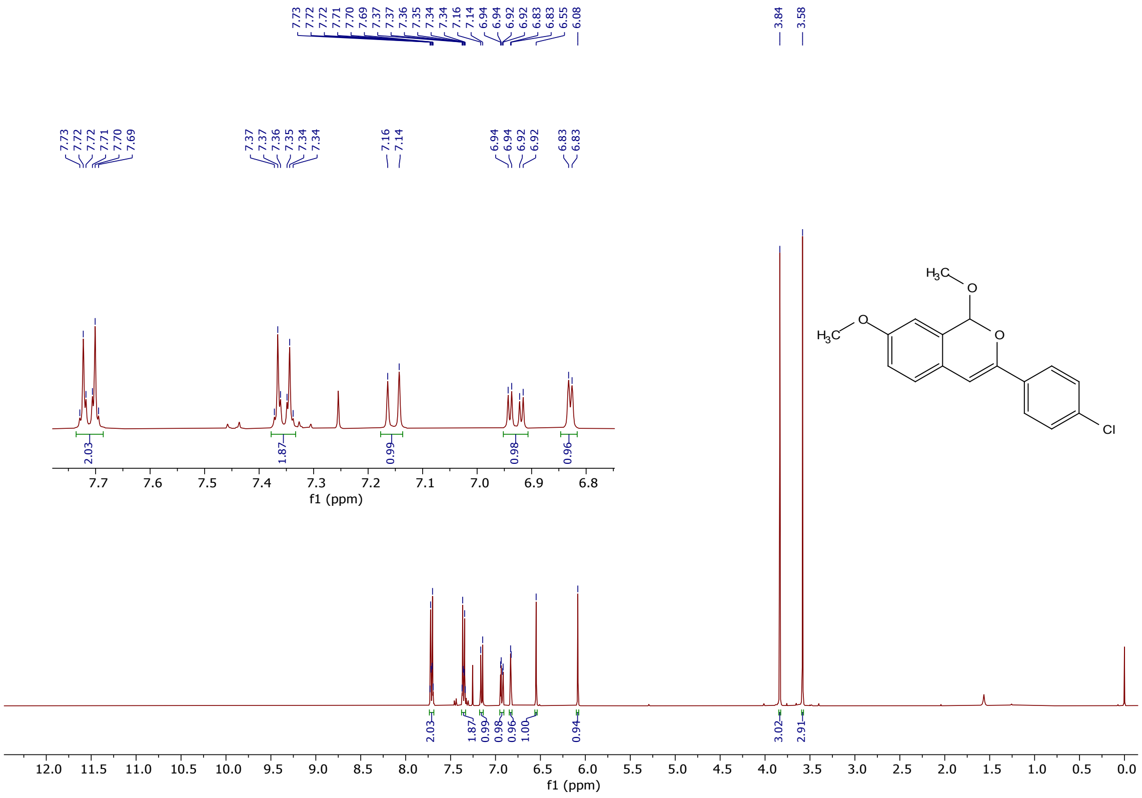

^{13}C{^{1}H} NMR (100 MHz, $CDCl_3$) of compound **2k**.

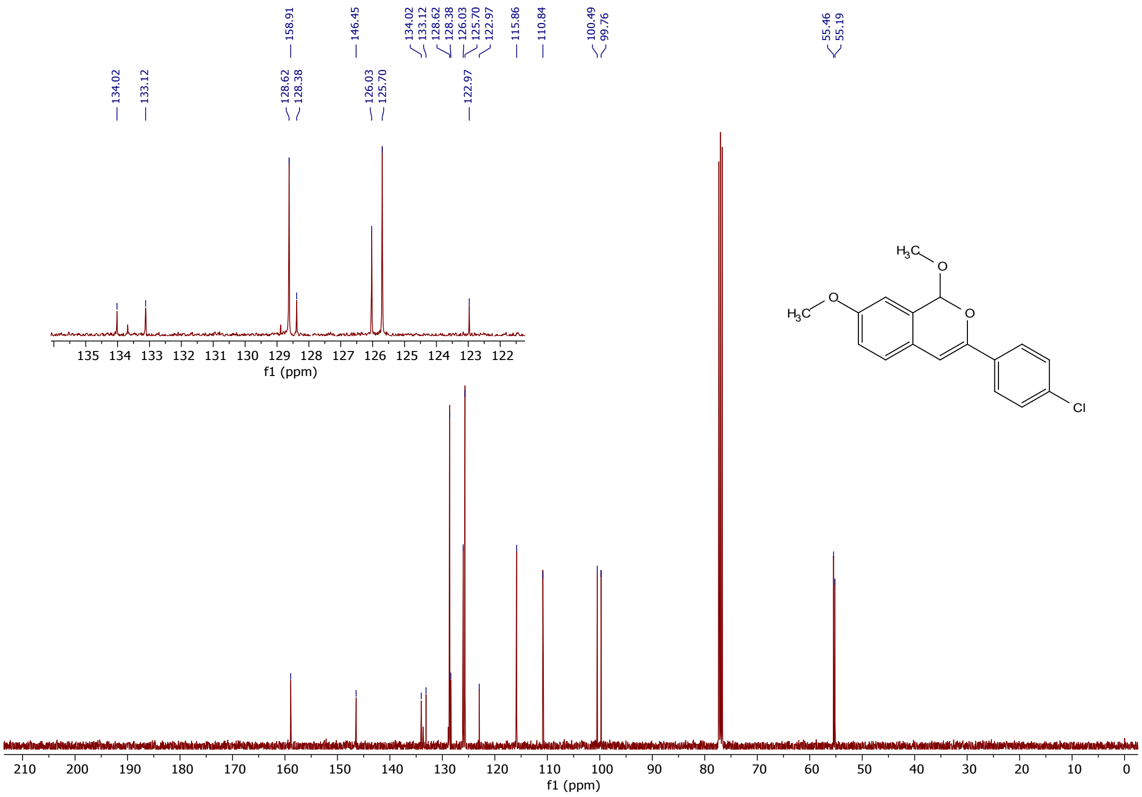

^{1}H NMR (400 MHz, $CDCl_3$) of compound **2l**.

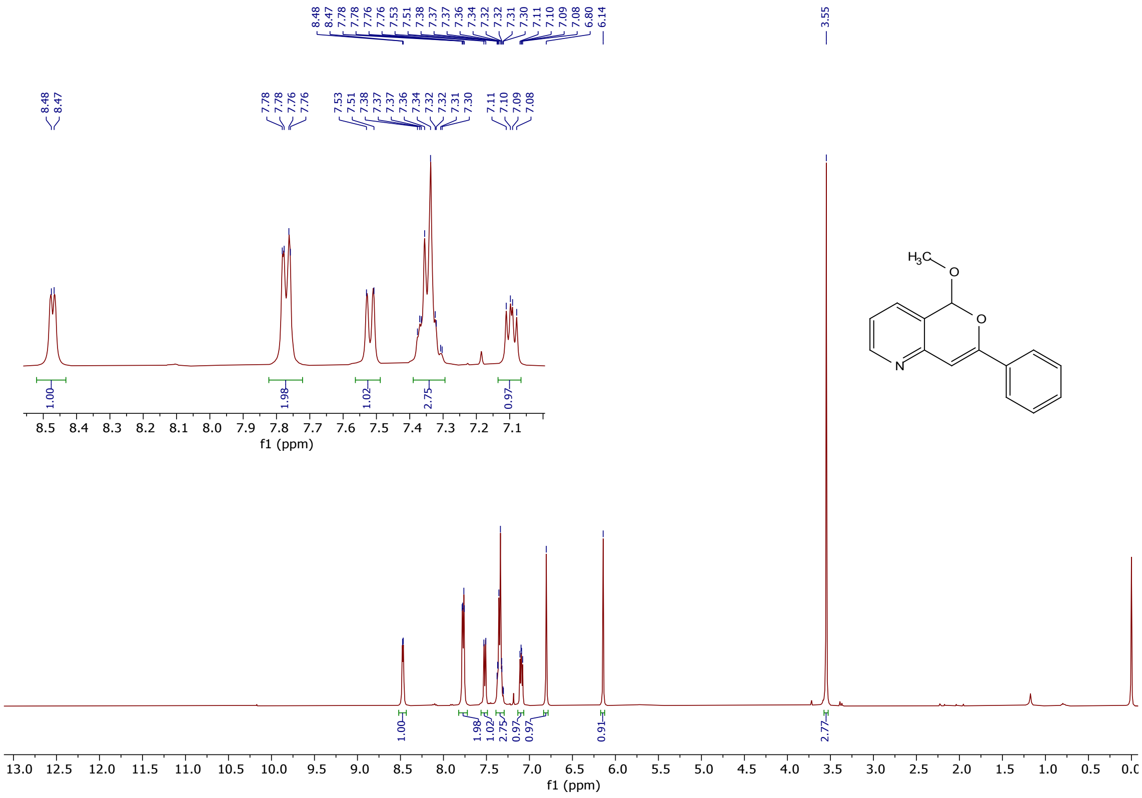

^{13}C{^{1}H} NMR (100 MHz, $CDCl_3$) of compound **2l**.

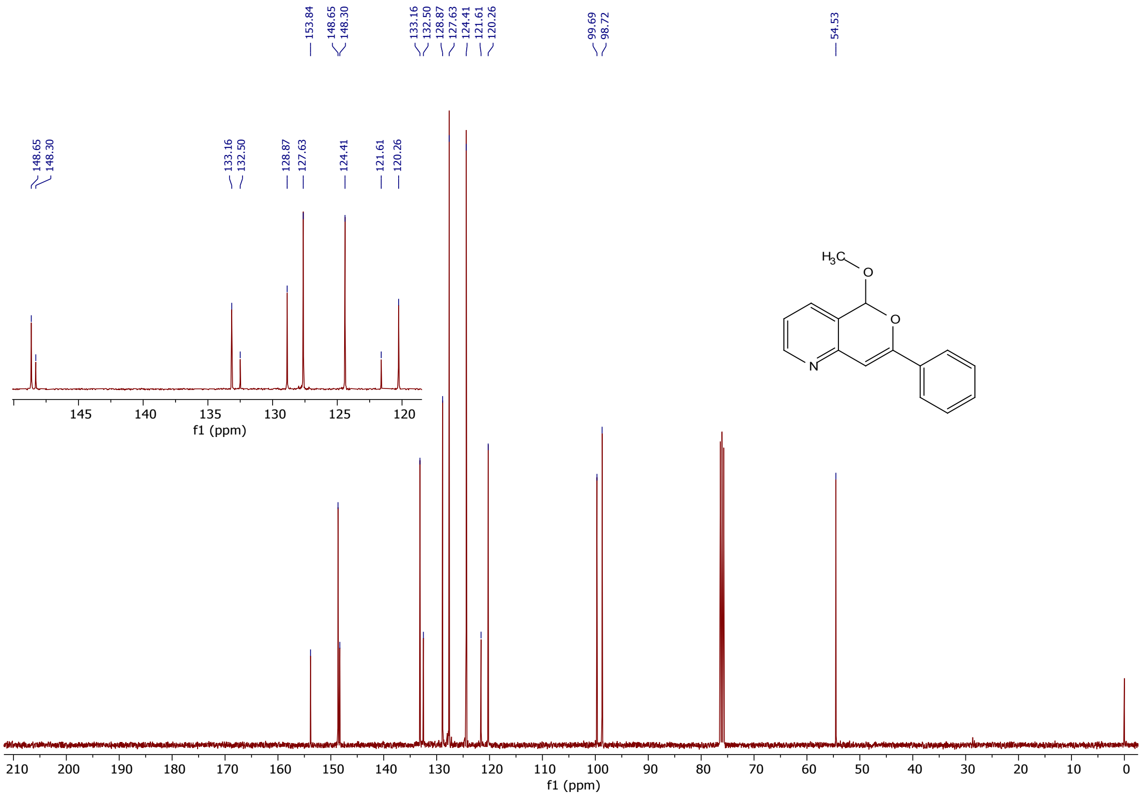

^{1}H NMR (400 MHz, DMSO-*d6*) of compound **2m**.

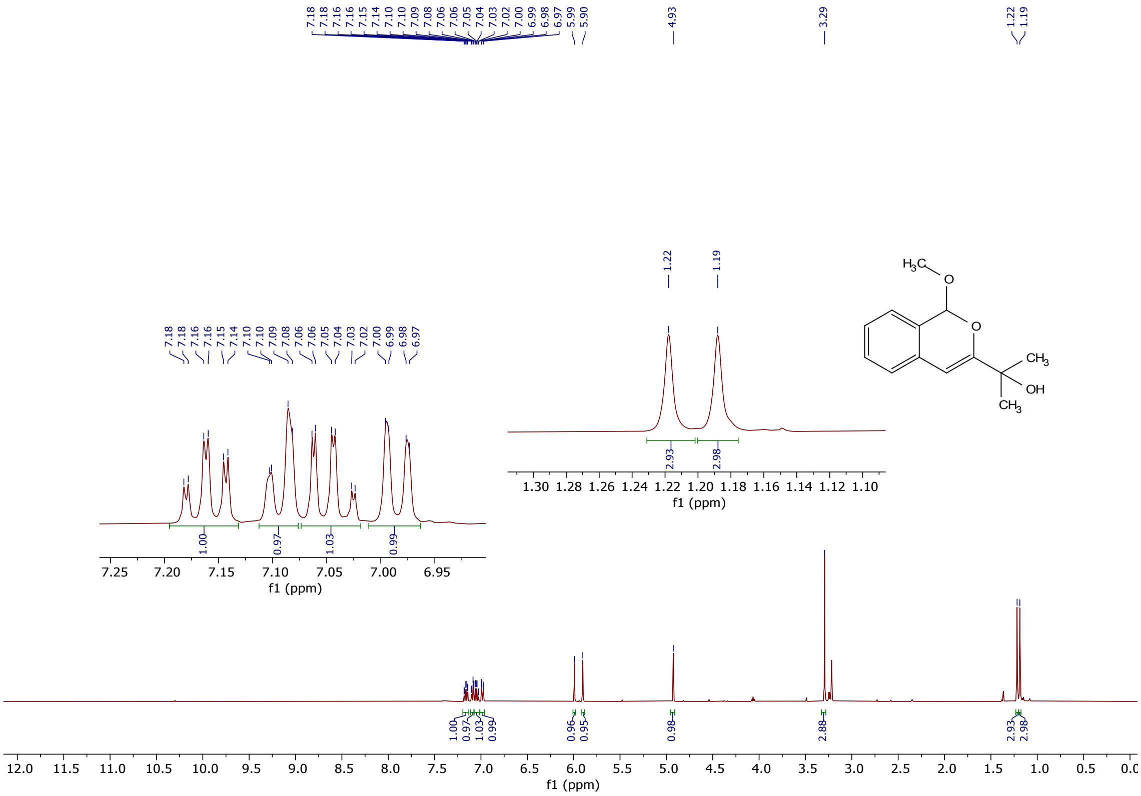

^{13}C{^{1}H} NMR (100 MHz, DMSO-*d6*) of compound **2m**.

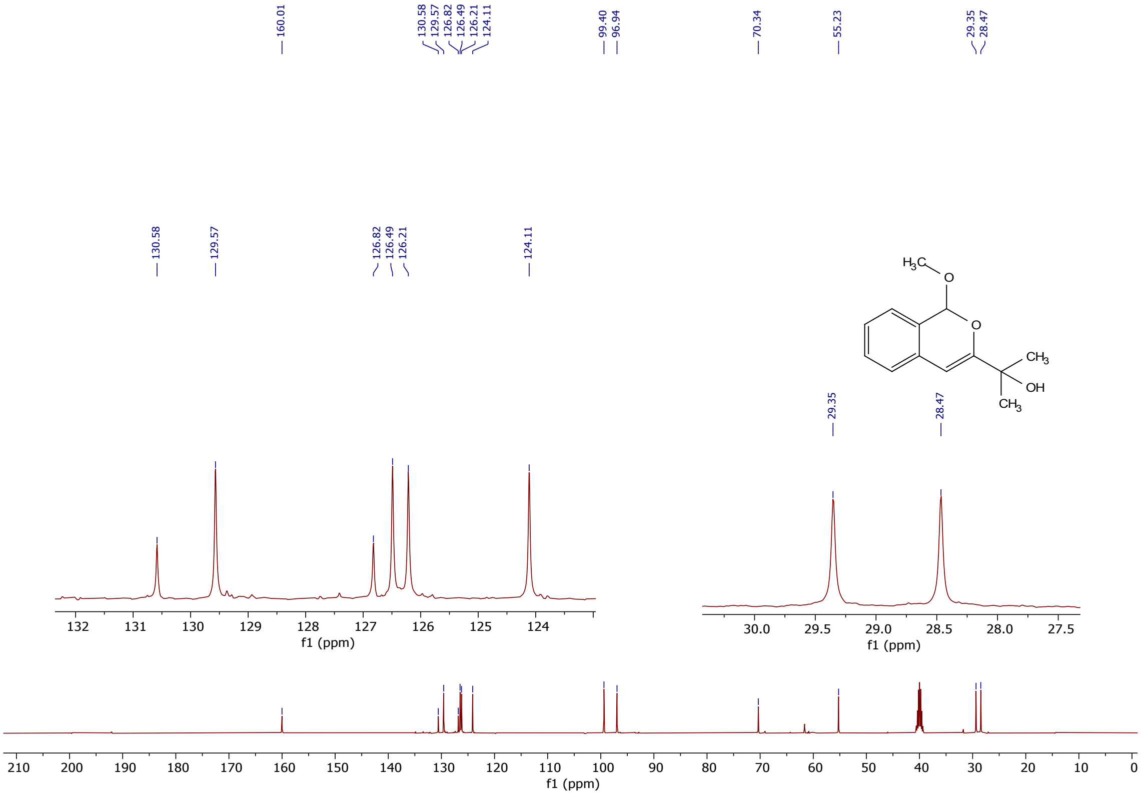

9.2 Compounds 3a-3e

^{1}H NMR (400 MHz, DMSO-*d6*) of compound **3a**

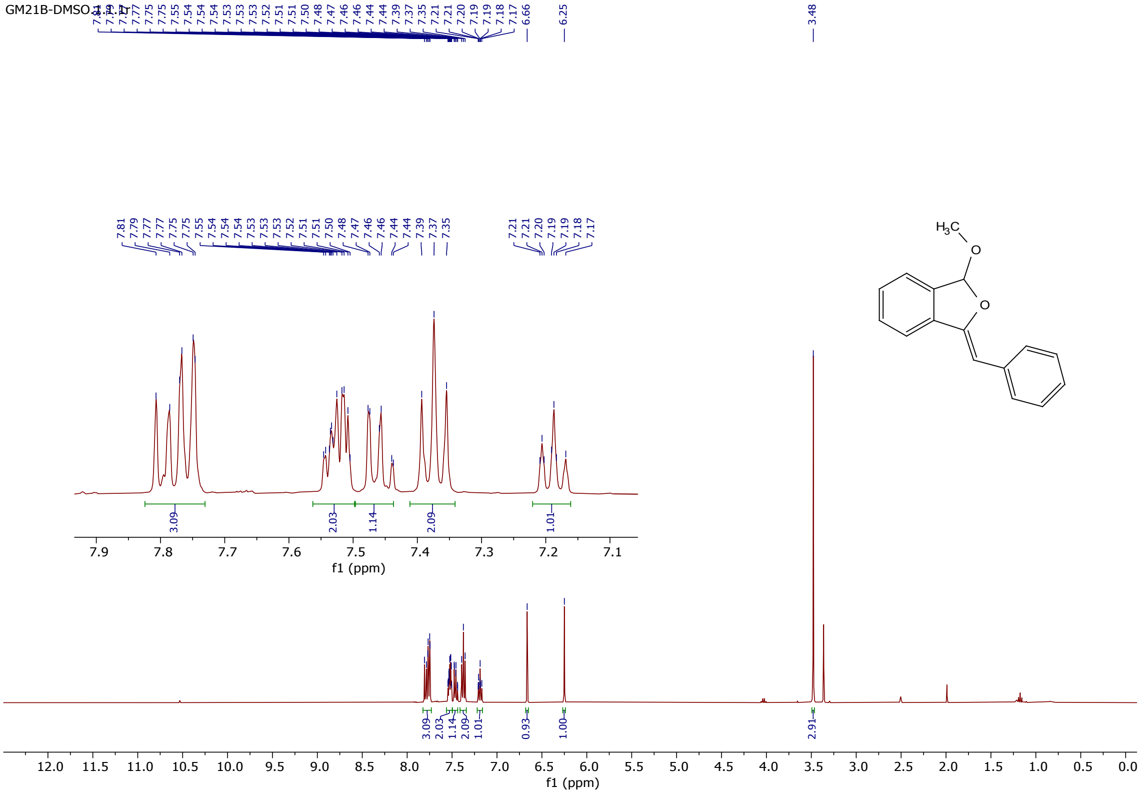

^{13}C{^{1}H} NMR (100 MHz, DMSO-*d6*) of compound **3a**.

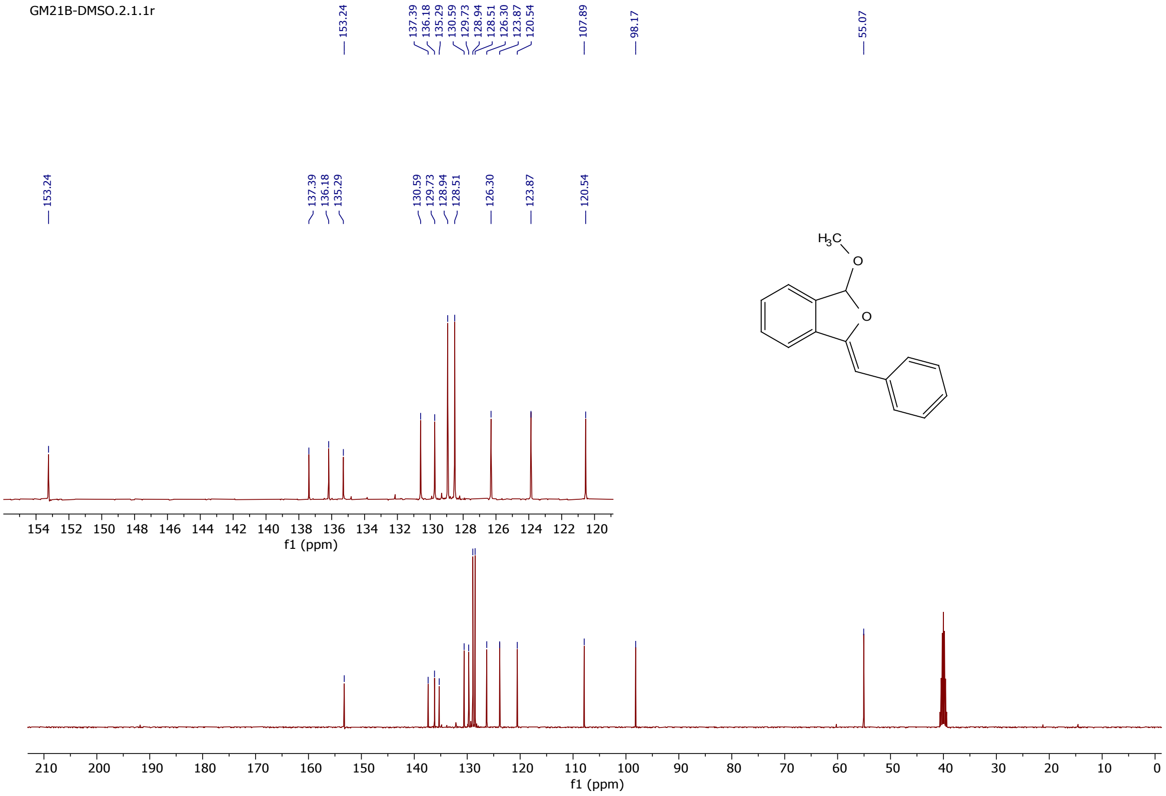

^{1}H NMR (400 MHz, DMSO-*d6*) of compound **3b**

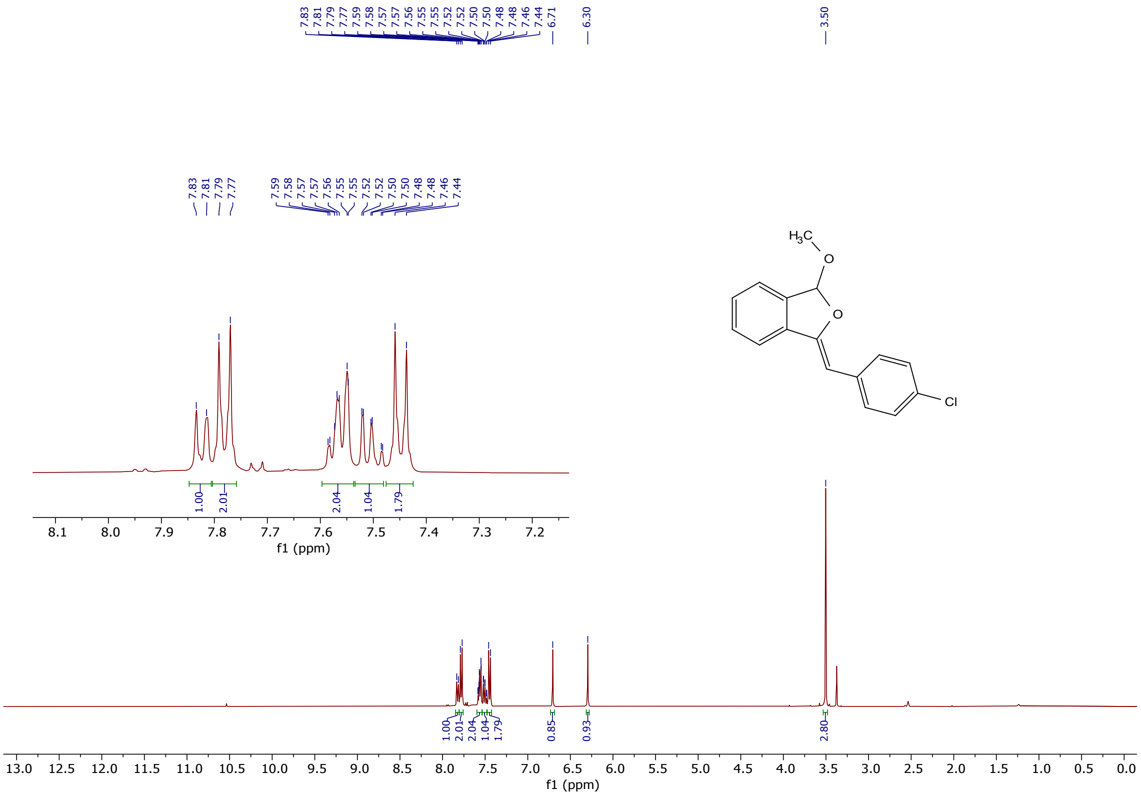

^{13}C{^{1}H} NMR (100 MHz, DMSO-*d6*) of compound **3b**.

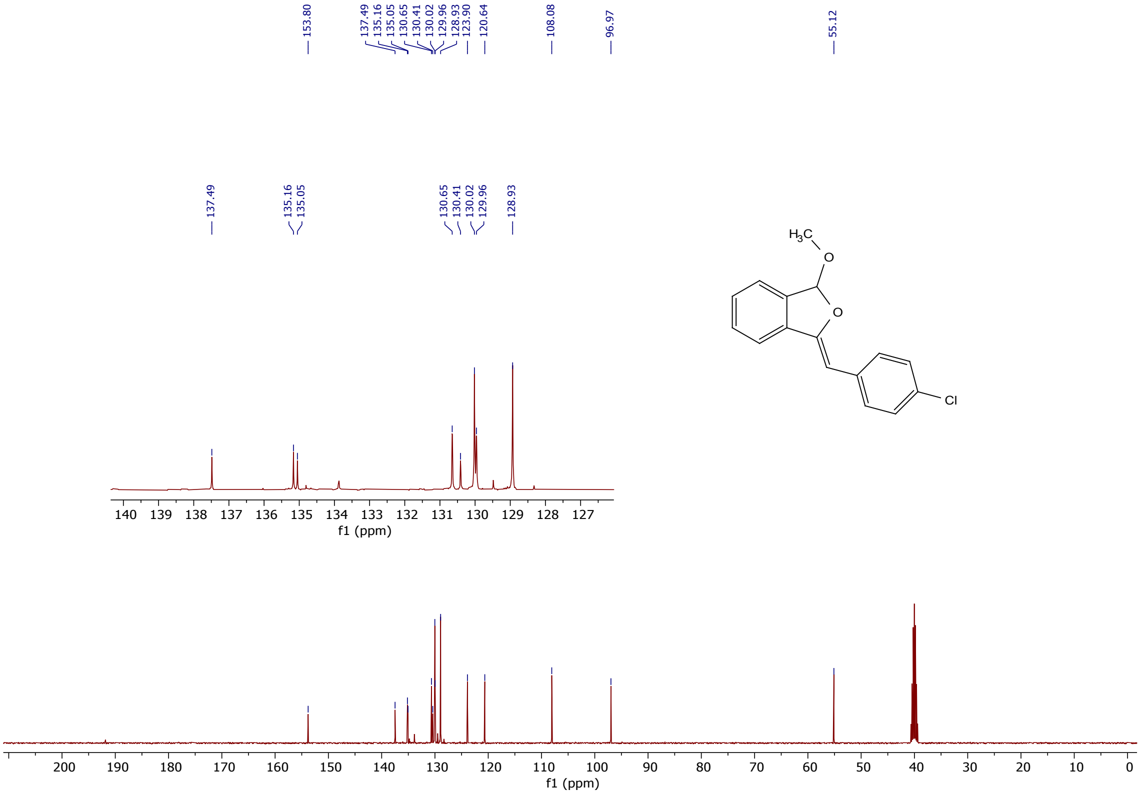

^{1}H NMR (400 MHz, DMSO-*d6*) of compound **3c**.

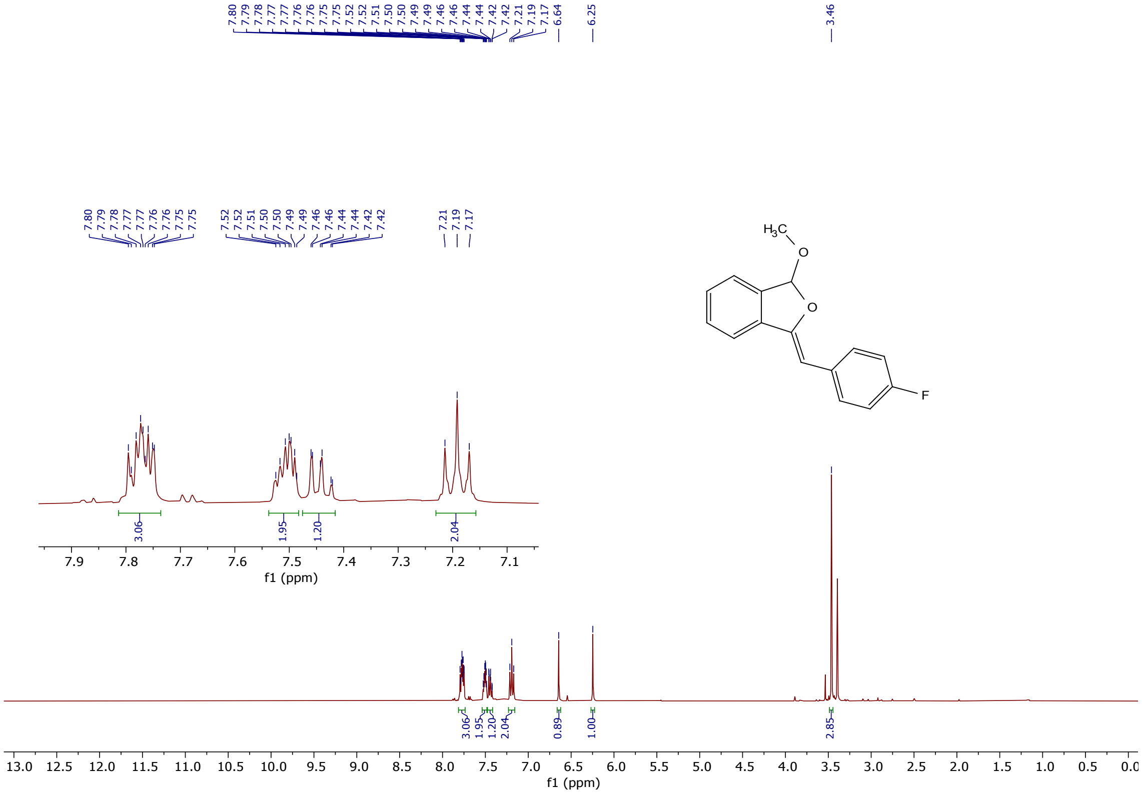

$^{13}C\{^{1}H\}$ NMR (100 MHz, DMSO-*d6*) of compound **3c**.

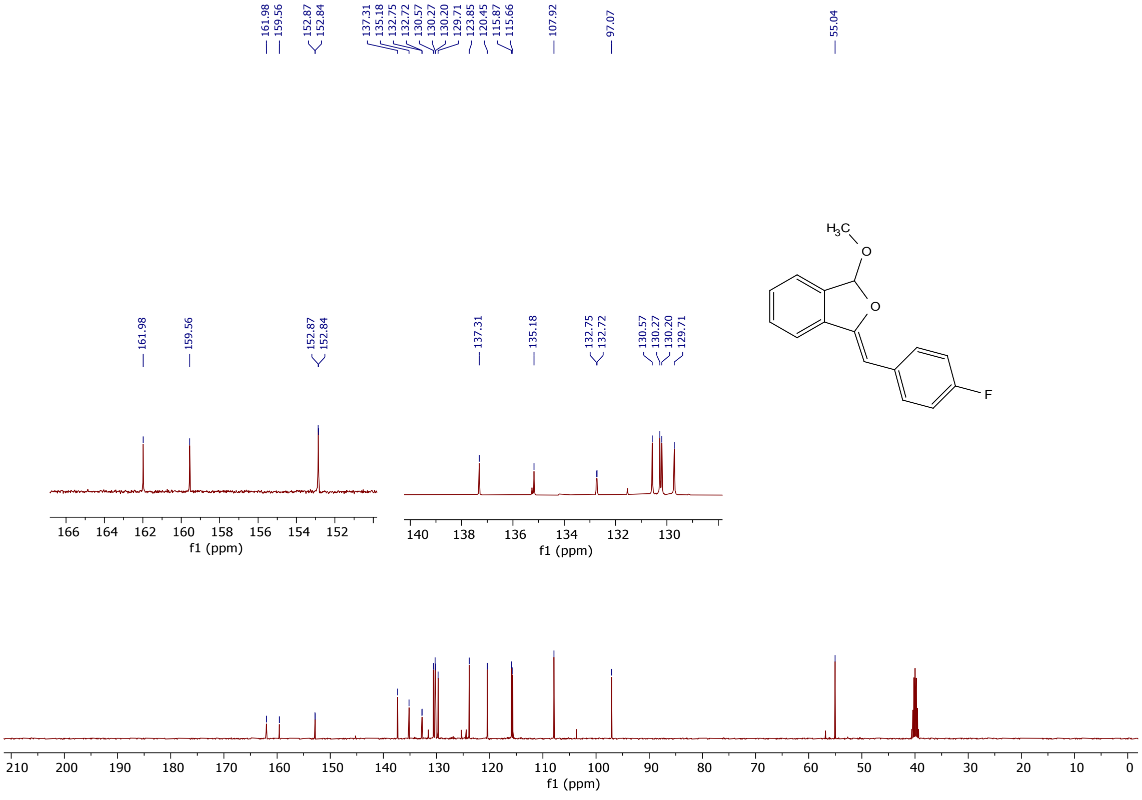

^{19}F NMR (377 MHz, DMSO-*d6*) of compound **3c**.

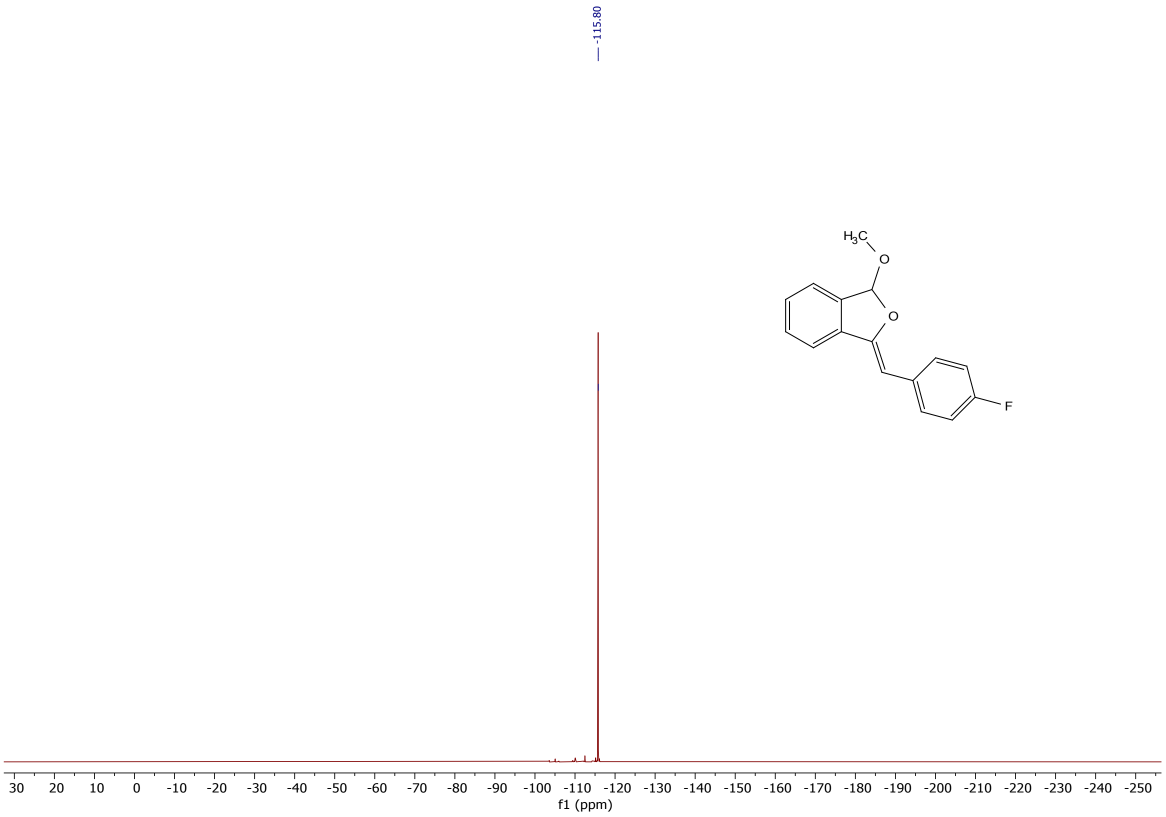

^{1}H NMR (400 MHz, DMSO-*d6*) of compound **3d**.

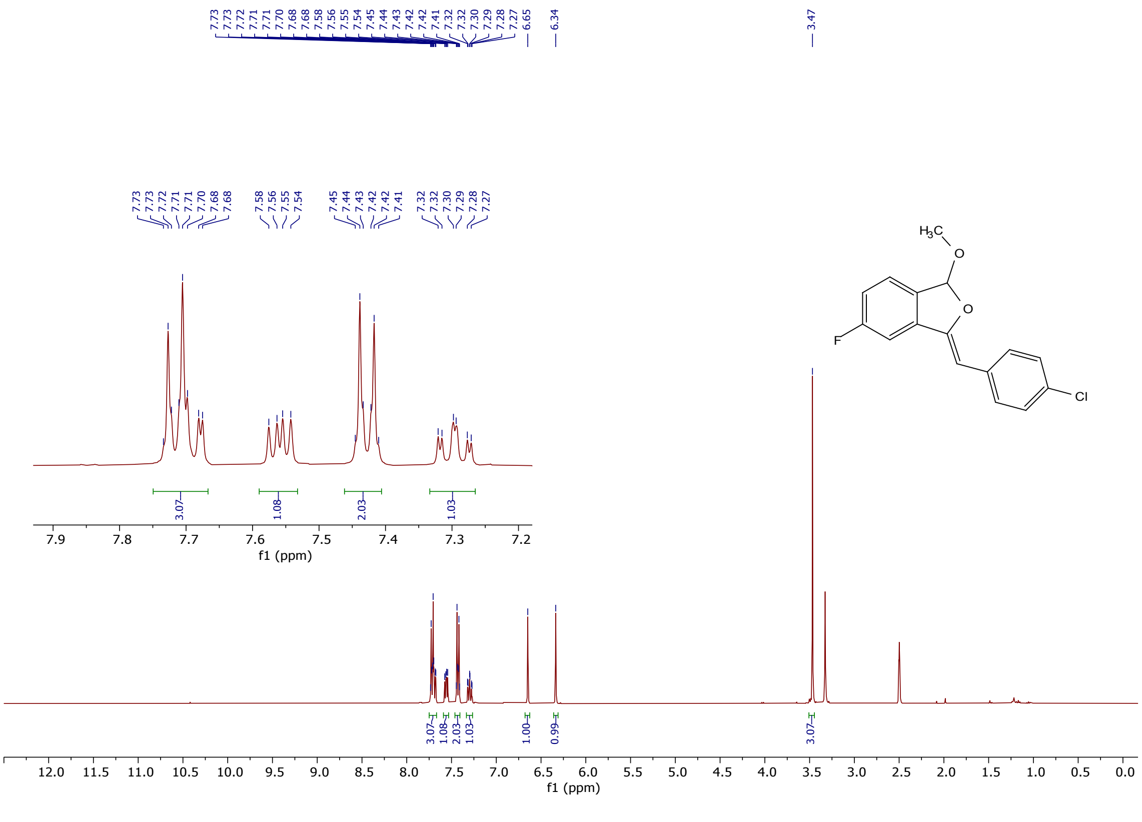

$^{13}C\{^1H\}$ NMR (100 MHz, DMSO-*d6*) of compound **3d**.

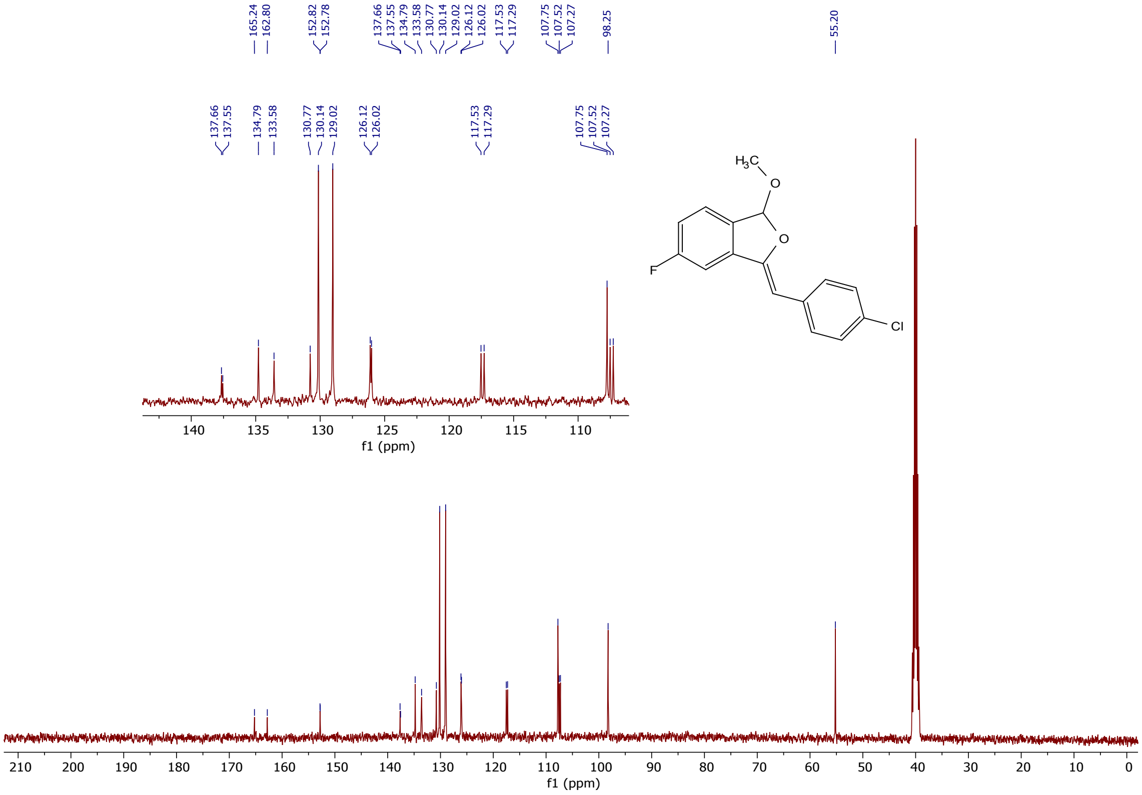

^{19}F NMR (377 MHz, DMSO-*d6*) of compound **3d**.

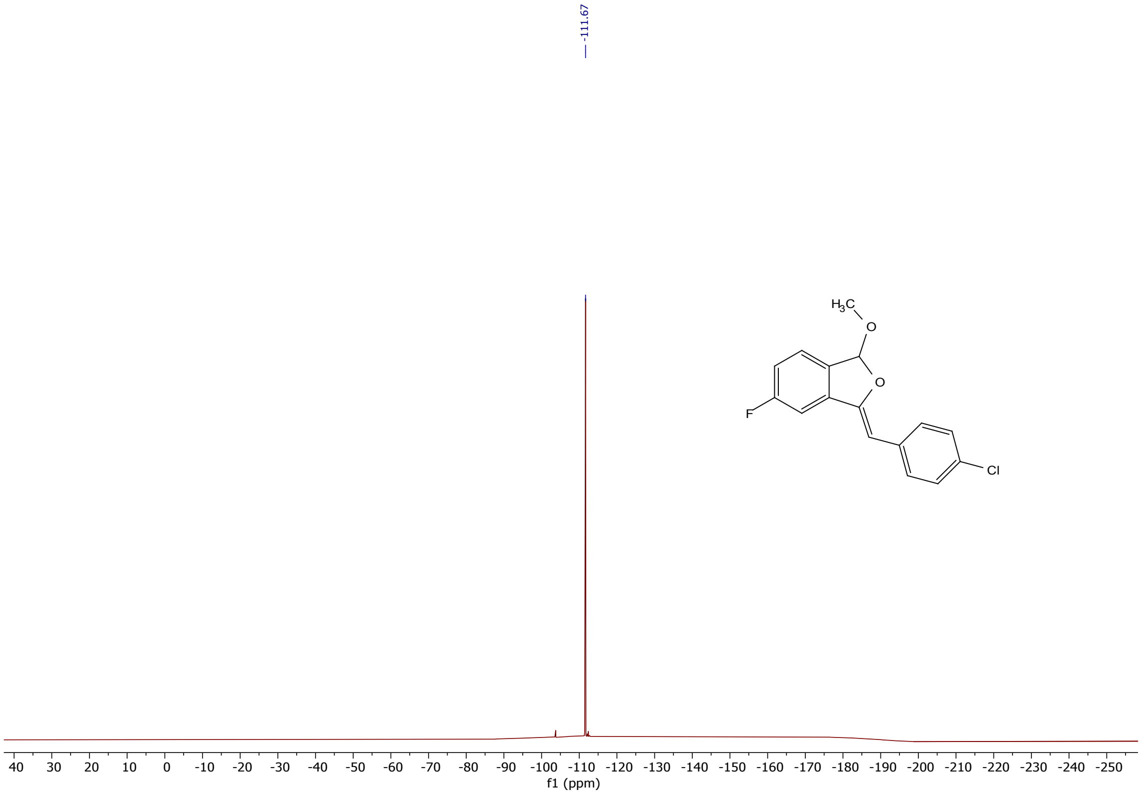

^{1}H NMR (400 MHz, DMSO-*d6*) of compound **3e**.

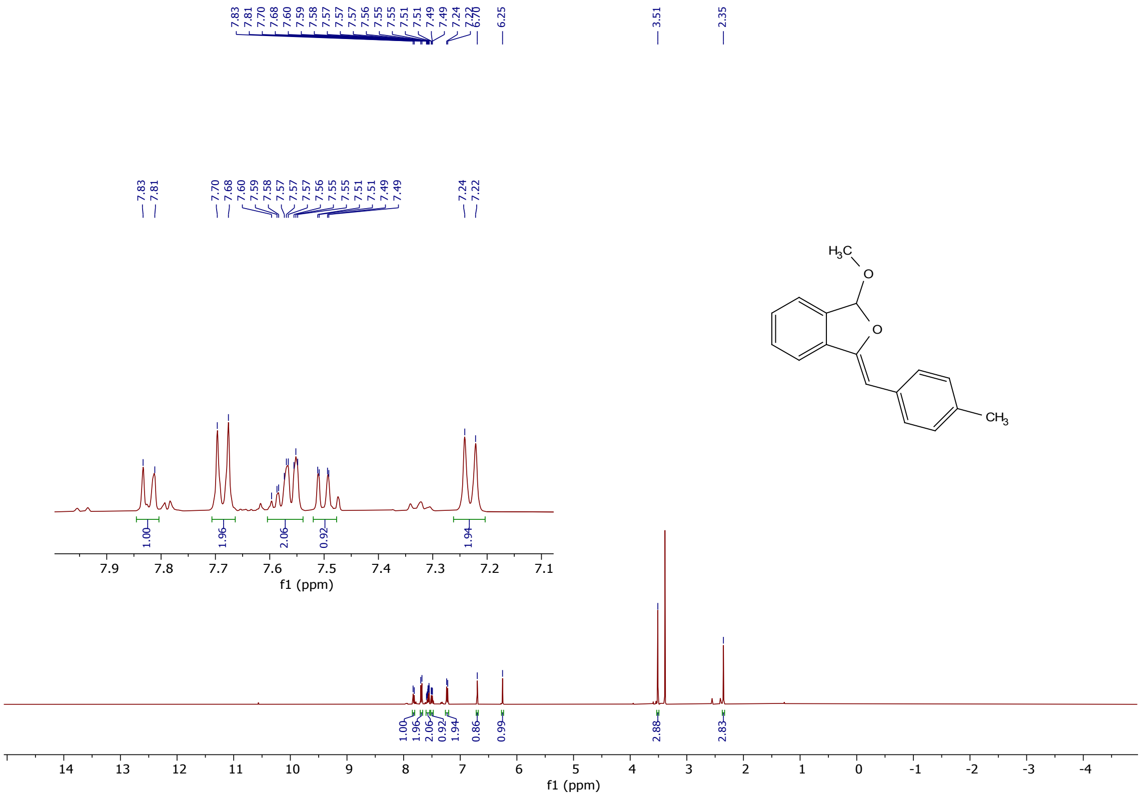

^{13}C{^{1}H} NMR (100 MHz, DMSO-*d6*) of compound **3e**.

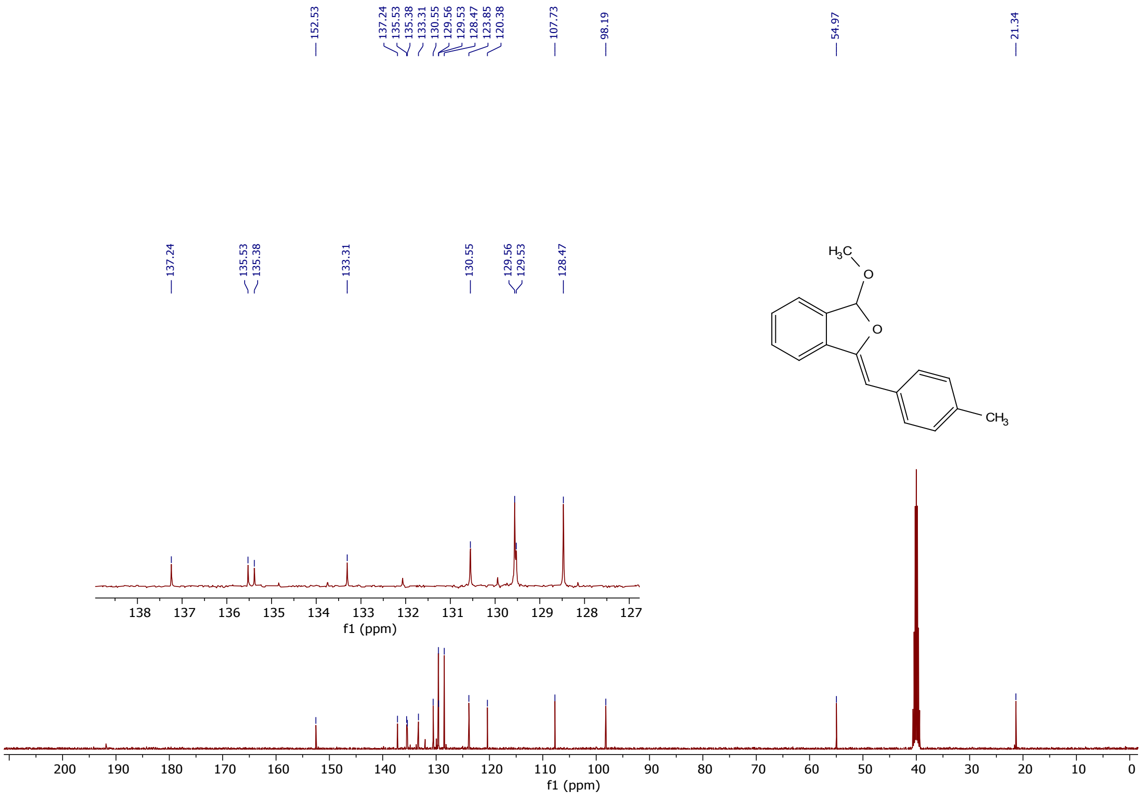

10. THEORETICAL CALCULATIONS - GENERAL REMARKS

The calculations were carried out using the Gaussian 09 package (revision D01).[10] The density functional theory (DFT) was employed in the optimization of the structures of all molecular complexes and transition states (TSs). The M06-2X functional (grid=ultrafine), the LanL2DZ basis set and the solvation model based on density (SMD) for *N,N*-dimethylformamide were used in the calculations.[11] The TSs were optimized using the Berny algorithm, presented a single imaginary frequency and were fully characterized through the analysis of the intrinsic reaction coordinate (IRC).[12] The thermodynamic properties were calculated using a temperature of 333.15 K (60 °C) (proposals 1-4) or 263.15 K (-10 °C) (proposal 5) and a pressure of 1 atm, aiming to precisely reproduce the experimental conditions. The vibrational analysis of each species was carried out to confirm the identity of all stationary points and in the determination of the thermal corrections to enthalpy and Gibbs free energy.

11. GENERAL OVERVIEW OF THE EVALUATED MECHANISMS

9.1 Proposal 1

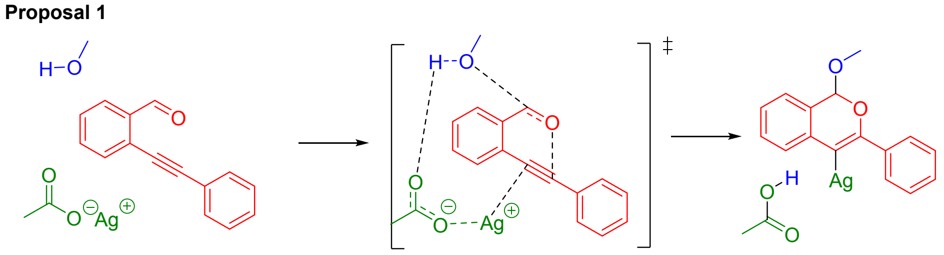

Note: for proposal 1, the 5-membered product formation was not evaluated due to the high barriers found for the 6-membered product pathway.

9.2 Proposal 2

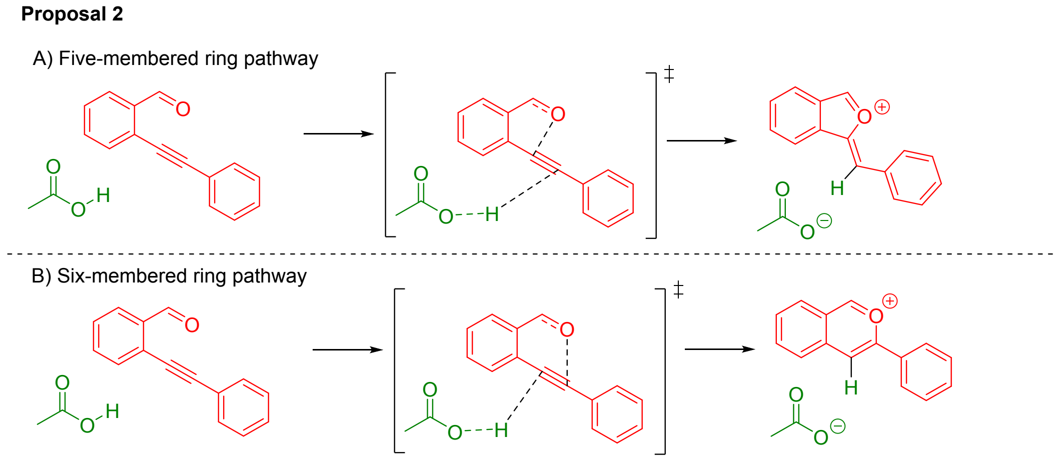

9.3 Proposal 3

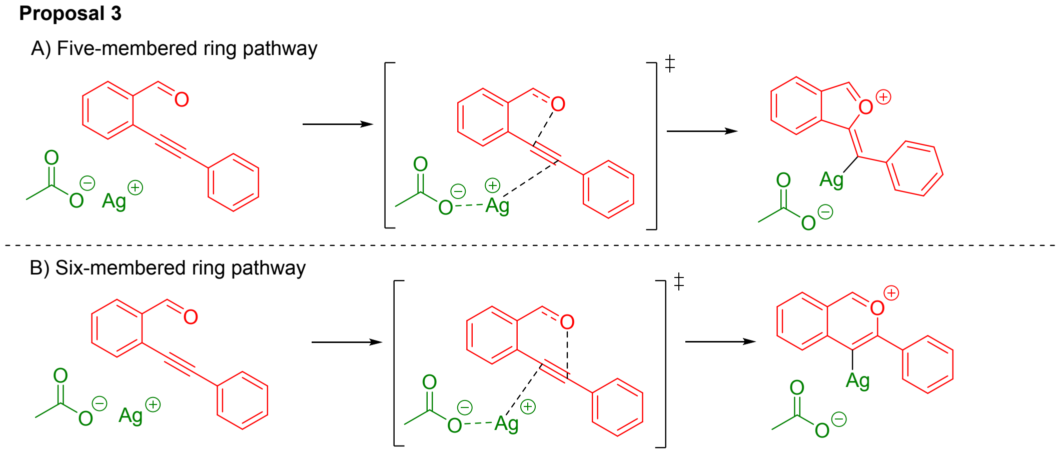

9.4 Proposal 4

Proposal 4

A) Five-membered ring pathway

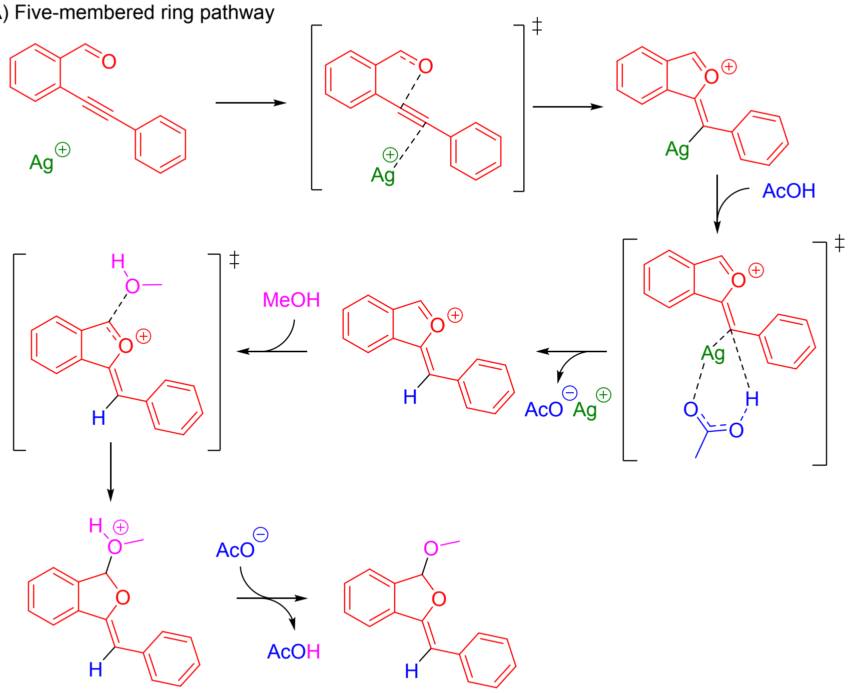

B) Six-membered ring pathway

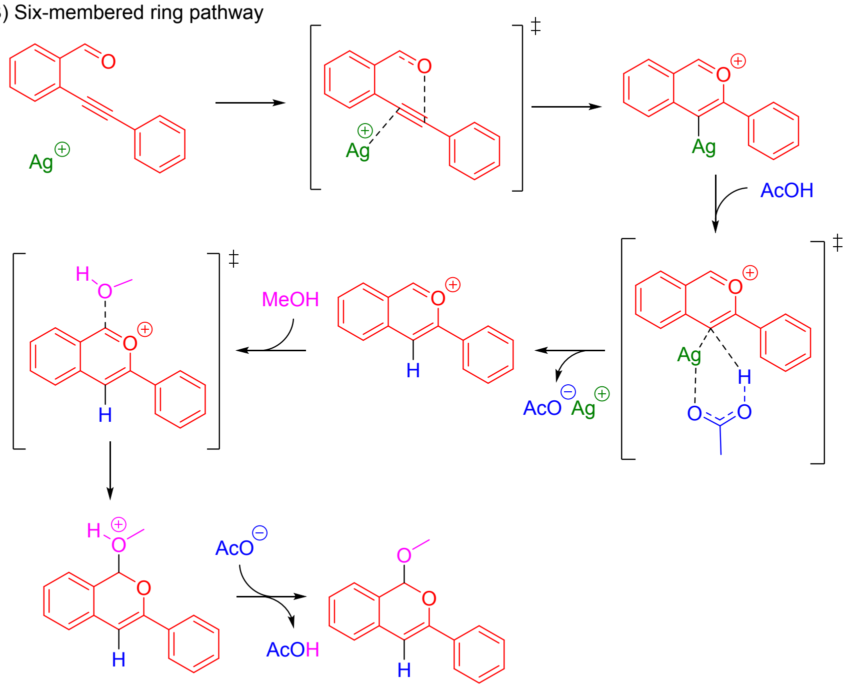

9.5 Proposal 5

Proposal 5

A) Five-membered ring pathway

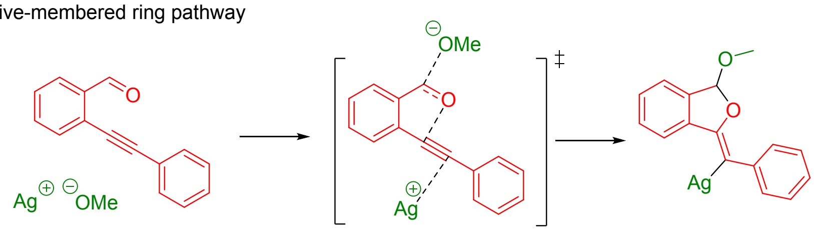

B) Six-membered ring pathway

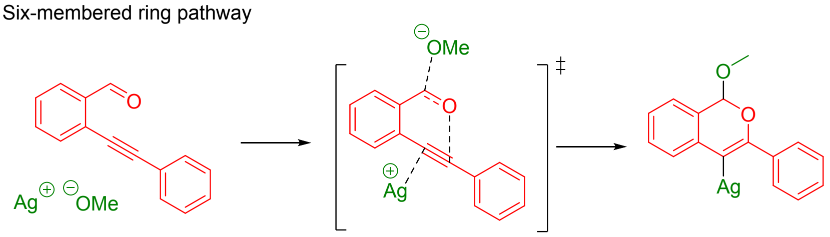

12. ENERGY PROFILE (ΔG) OF THE EVALUATED MECHANISMS

Figure S9. Proposal 1 (6-membered ring pathway)

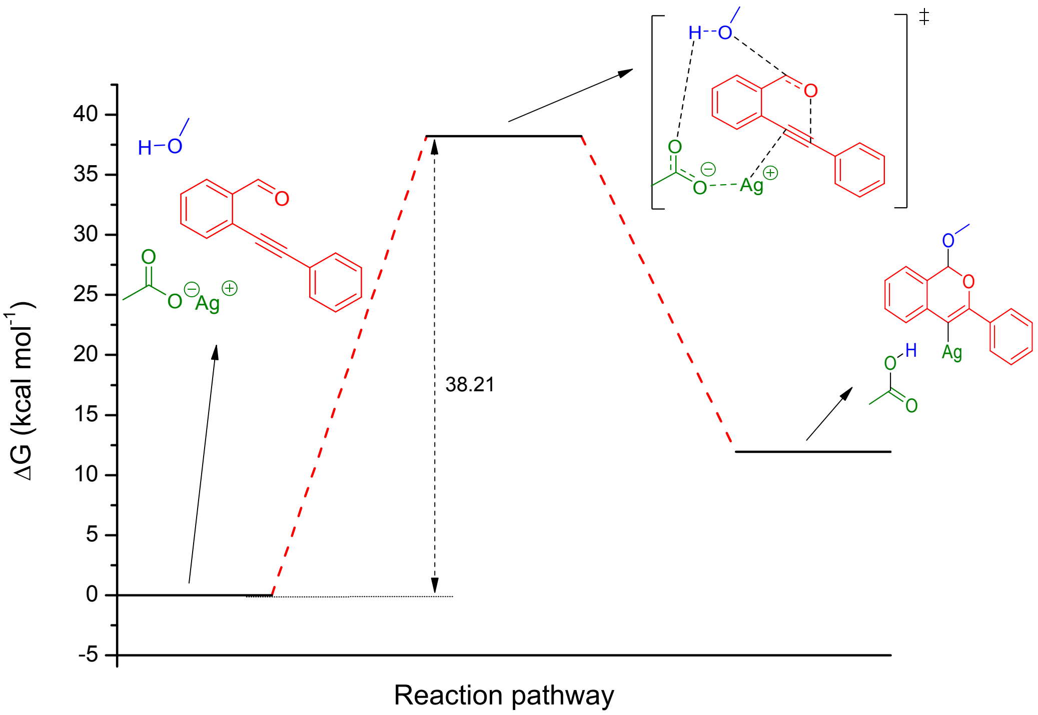

Figure S10. Proposal 2 (5-membered ring pathway)

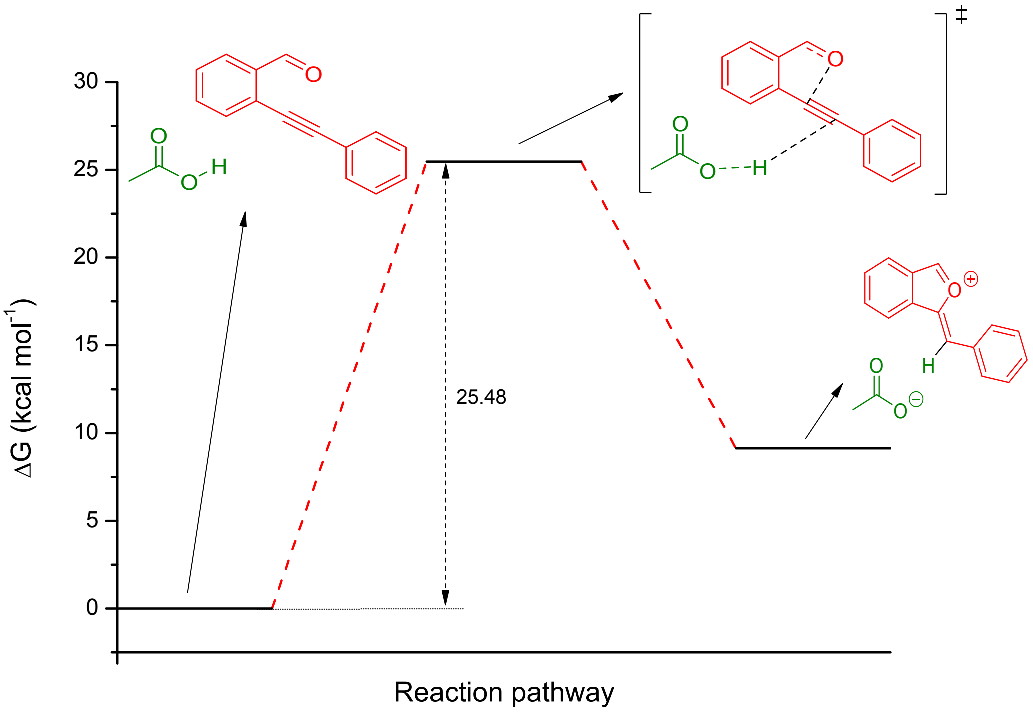

Figure S11. Proposal 2 (6-membered ring pathway)

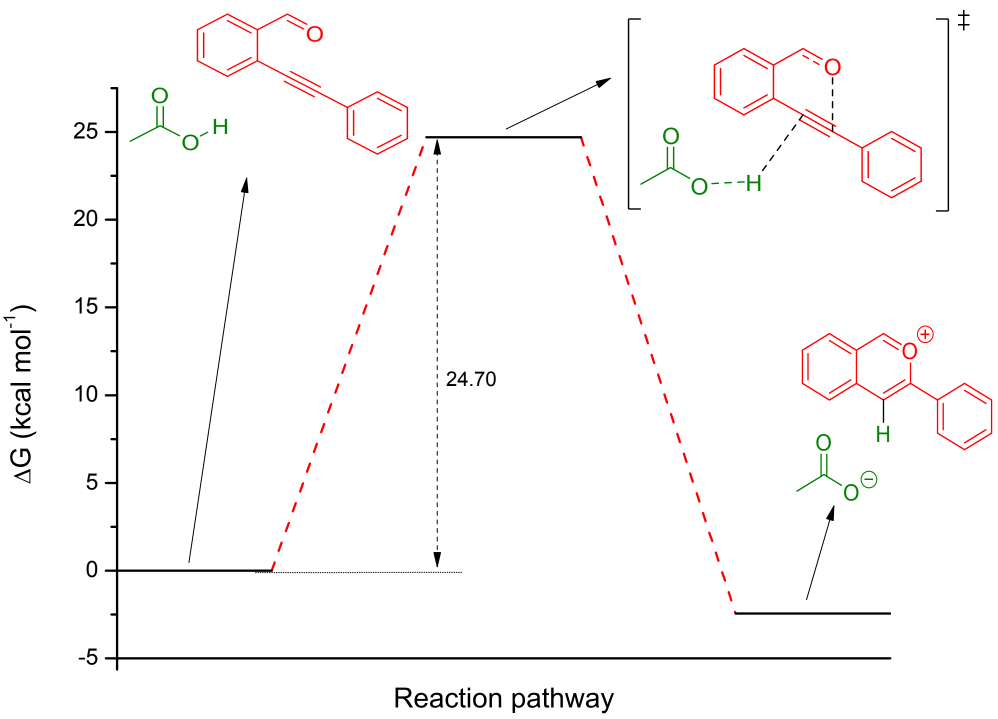

Figure S12. Proposal 3 (5-membered ring pathway)

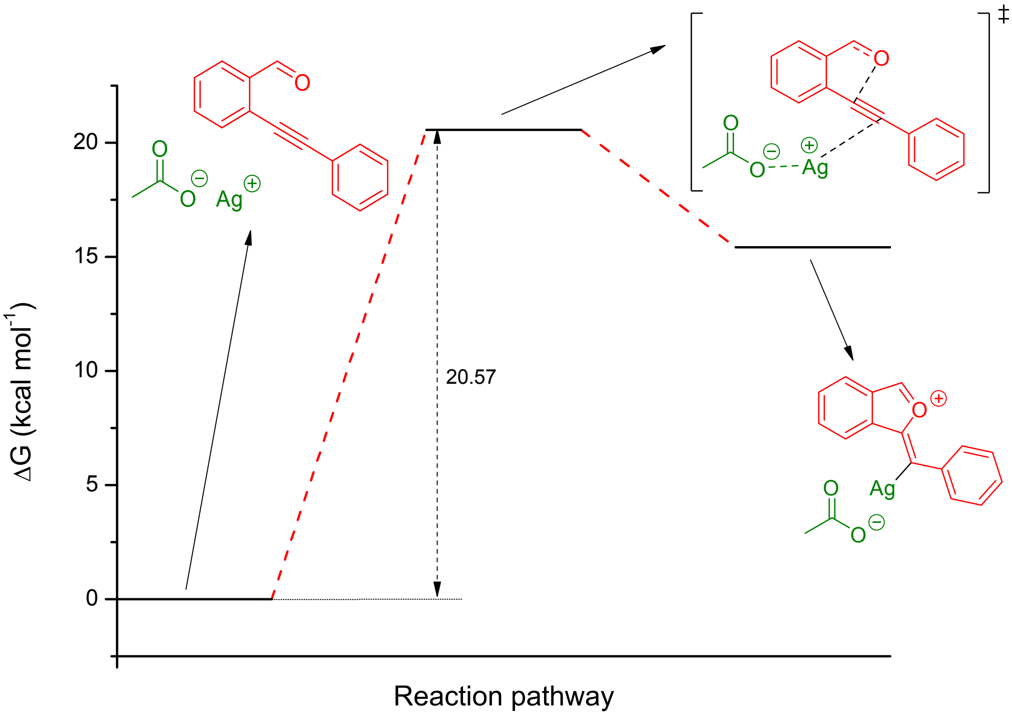

Figure S13. Proposal 3 (6-membered ring pathway)

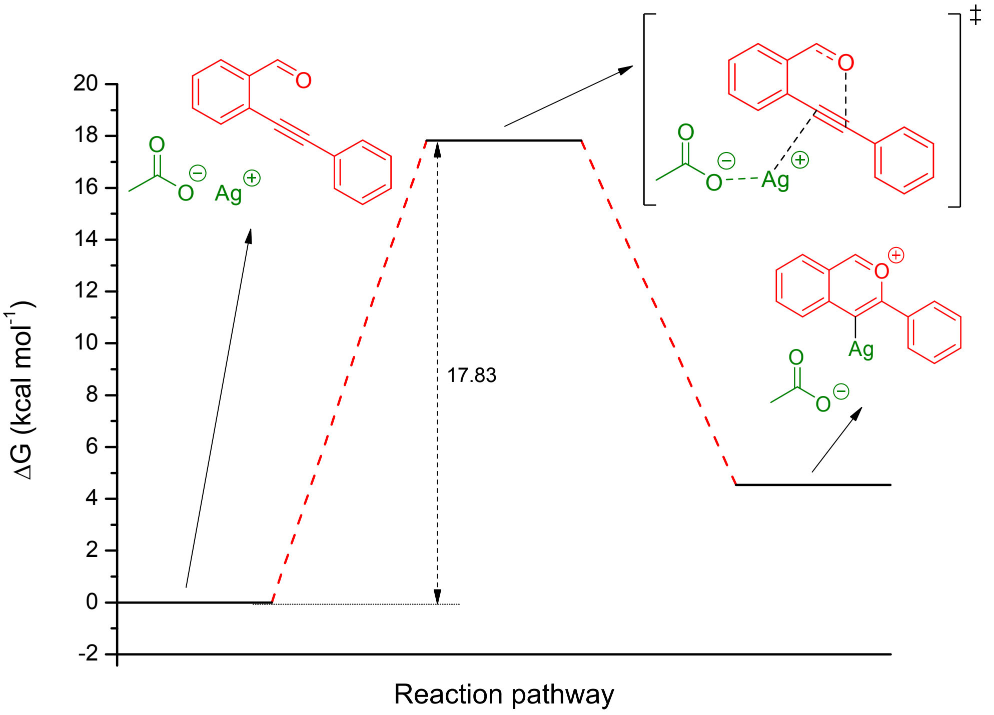

– – +

Figure S14. Proposal 4 (5-membered ring pathway)

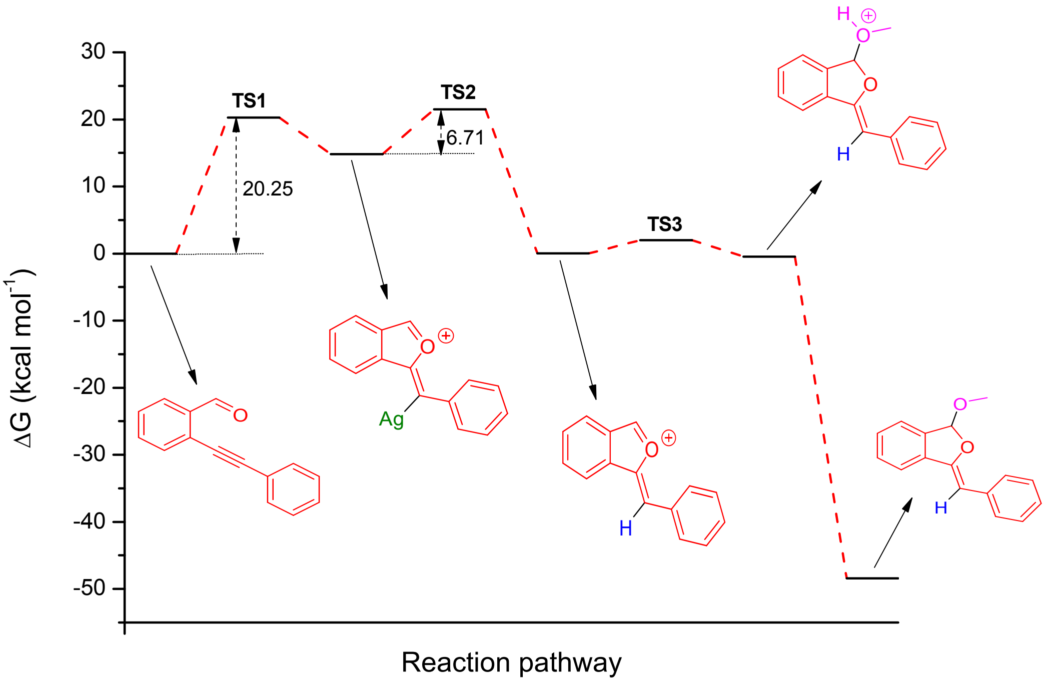

Note: The other species involved in each step were omitted for clarity (for the full mechanism see the "General overview of the evaluated mechanisms" topic)

Figure S14. Proposal 4 (6-membered ring pathway)

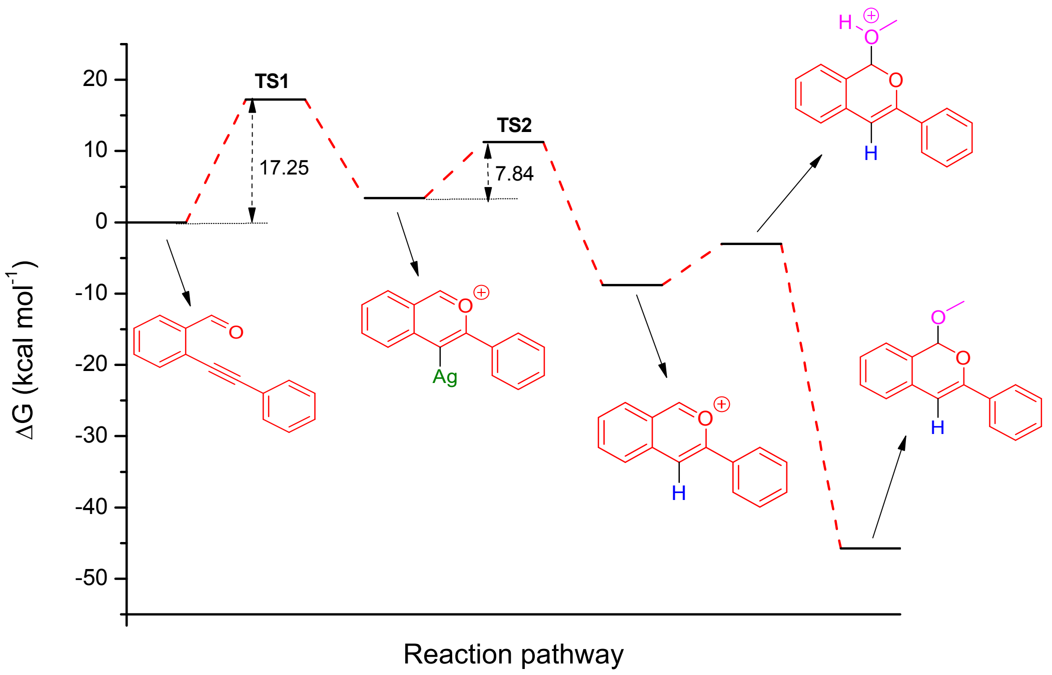

Notes: 1) The other species involved in each step were omitted for clarity (for the full mechanism see the "General overview of the evaluated mechanisms" topic); 2) Step 3 is barrierless in terms of ΔG at this level of theory.

Figure S16. Proposal 5 (5-membered ring pathway)

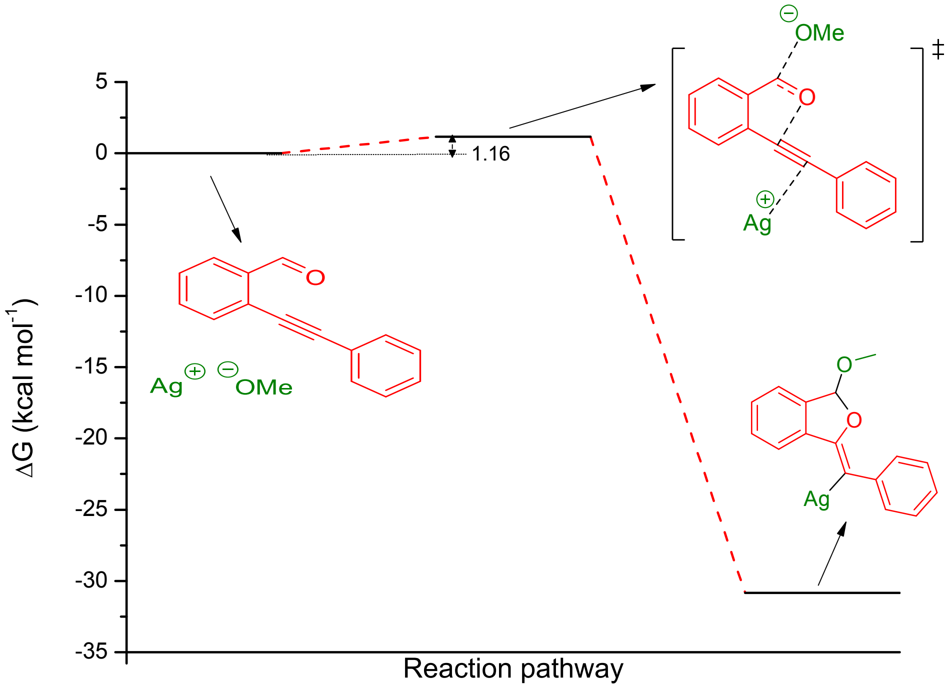

Figure S17. Proposal 5 (6-membered ring pathway)

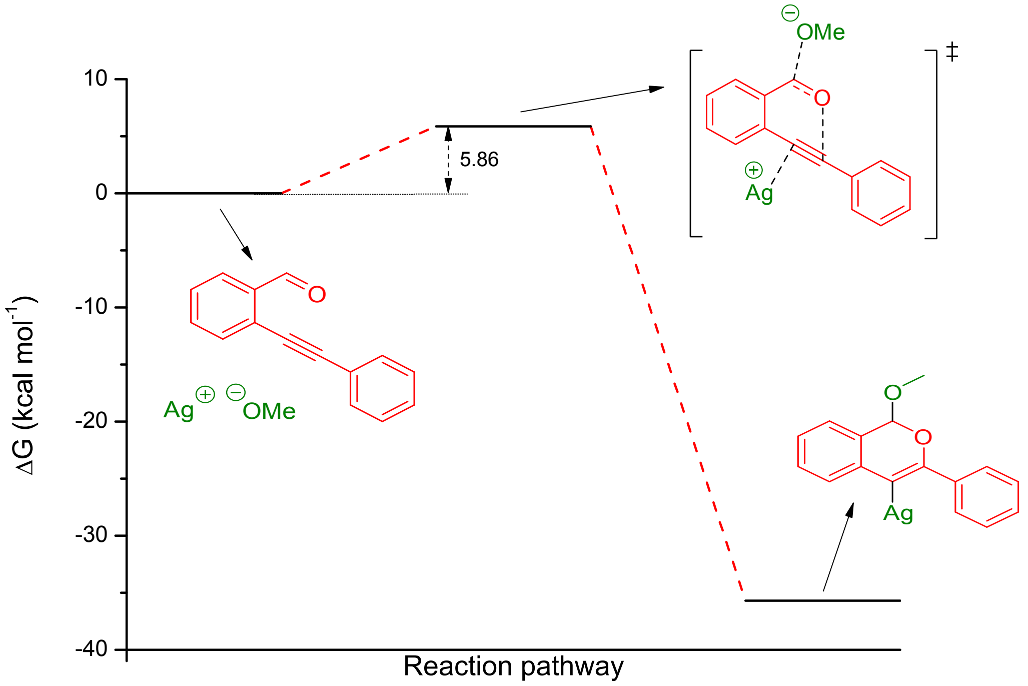

13. IMAGINARY FREQUENCIES FOR ALL TRANSITION STATES, INTRINSIC REACTION COORDINATES AND SCAN DATA

Table S1. Imaginary frequencies of all TS structures.

Transition state	Frequency (cm^{-1})
**Proposal 1 (6-membered ring pathway)**	-430.77
**Proposal 2 (5-membered ring pathway)**	-535.87
**Proposal 2 (6-membered ring pathway)**	-500.05
**Proposal 3 (5-membered ring pathway)**	-477.77
**Proposal 3 (6-membered ring pathway)**	-462.71
**Proposal 4 (5-membered ring pathway) – Step 1**	-477.75
**Proposal 4 (6-membered ring pathway) – Step 1**	-464.36
**Proposal 4 (5-membered ring pathway) – Step 2**	-118.34
**Proposal 4 (6-membered ring pathway) – Step 2**	-102.88
**Proposal 4 (5-membered ring pathway) – Step 3**	-176.45
**Proposal 4 (6-membered ring pathway) – Step 3**	-210.35
**Proposal 5 (5-membered ring pathway)**	-174.86
**Proposal 5 (6-membered ring pathway)**	-284.70

Figure S18. Figure Intrinsic Reaction Coordinate – Proposal 1 (6-membered ring pathway)

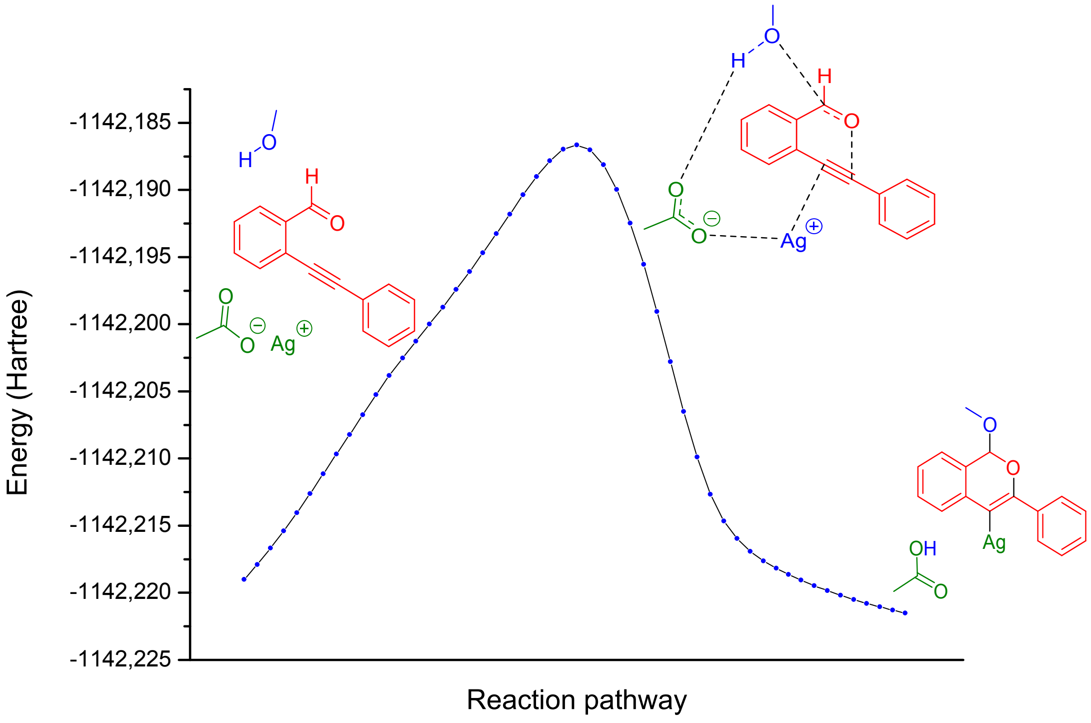

Figure S19. Figure Intrinsic Reaction Coordinate – Proposal 2 (5-membered ring pathway)

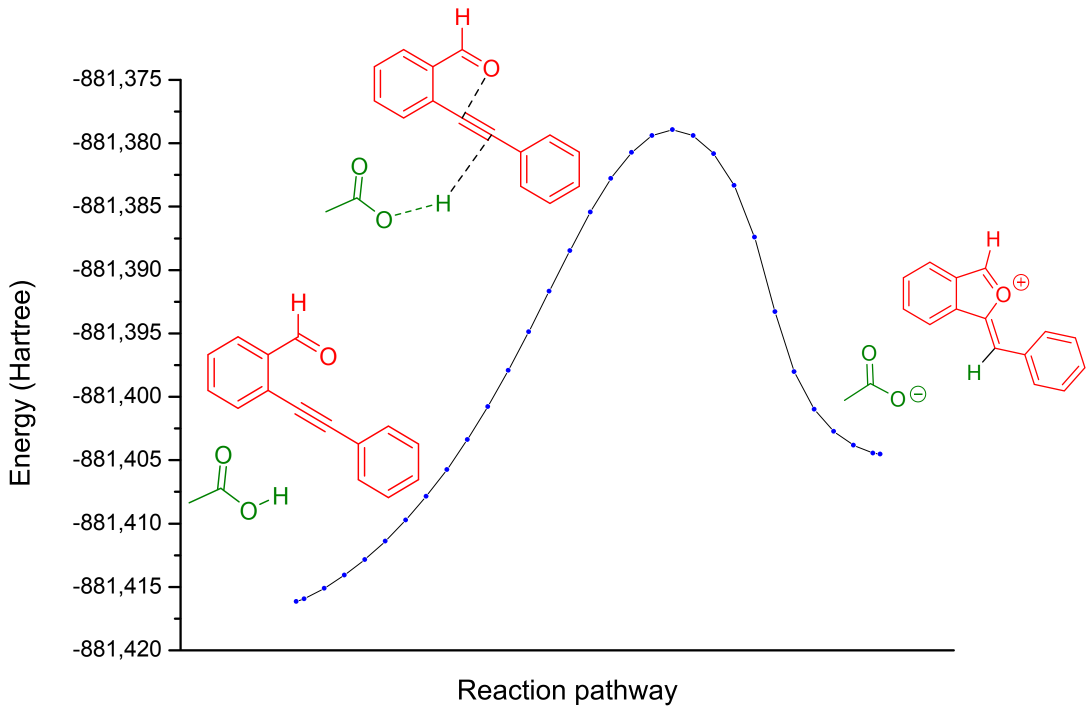

Figure S20. Figure Intrinsic Reaction Coordinate – Proposal 2 (6-membered ring pathway)

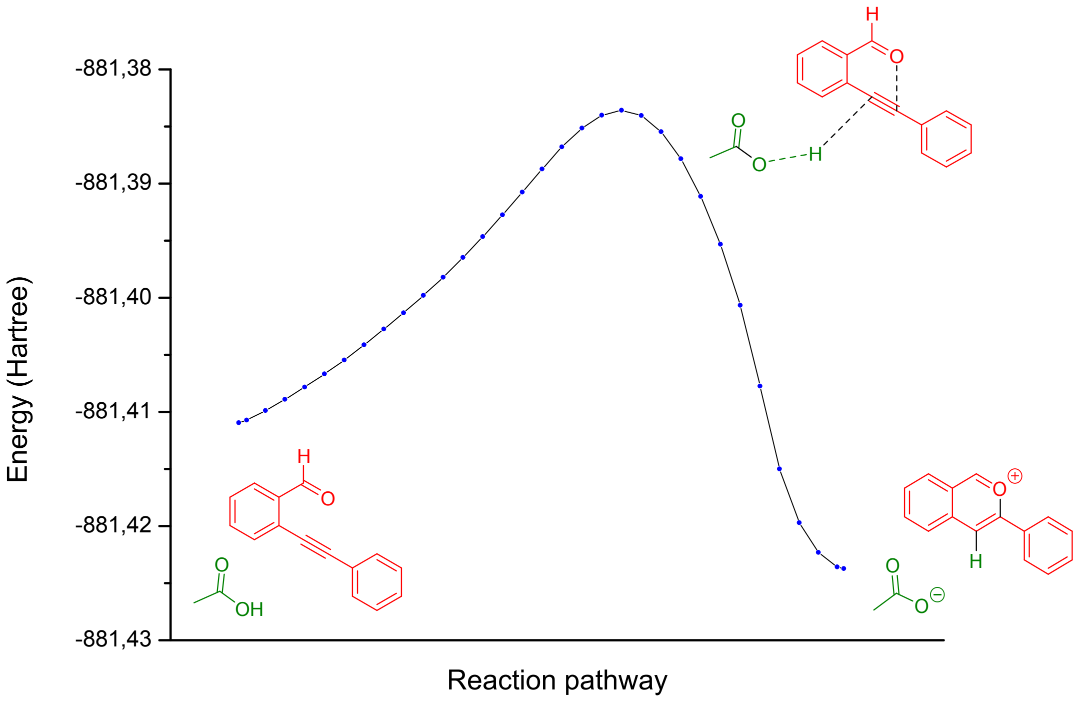

Figure S21. Figure Intrinsic Reaction Coordinate – Proposal 3 (5-membered ring pathway)

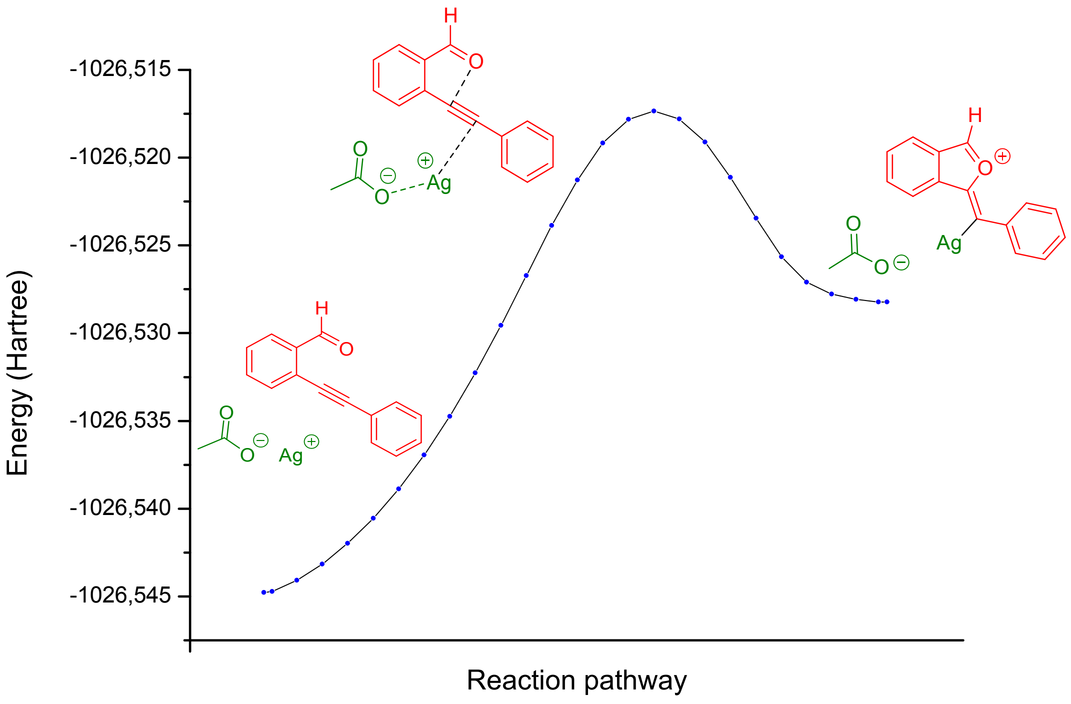

Figure S22. Figure Intrinsic Reaction Coordinate – Proposal 3 (6-membered ring pathway)

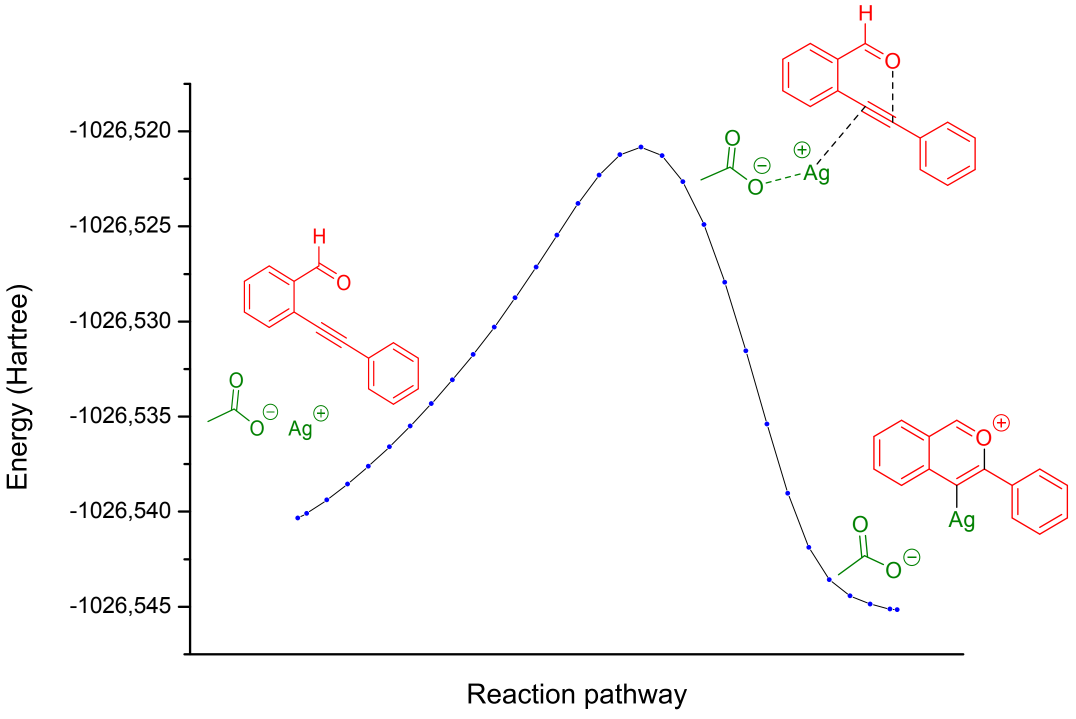

Figure S23. Figure Intrinsic Reaction Coordinate – Proposal 4 (5-membered ring pathway – first step)

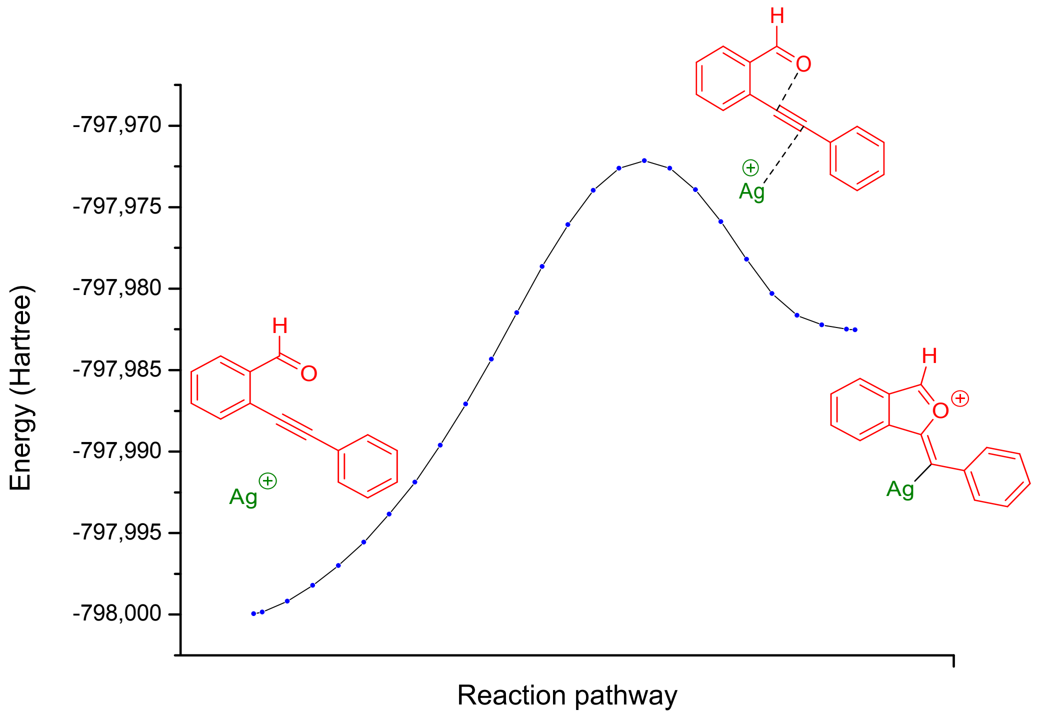

Figure S24. Figure Intrinsic Reaction Coordinate – Proposal 4 (6-membered ring pathway – first step)

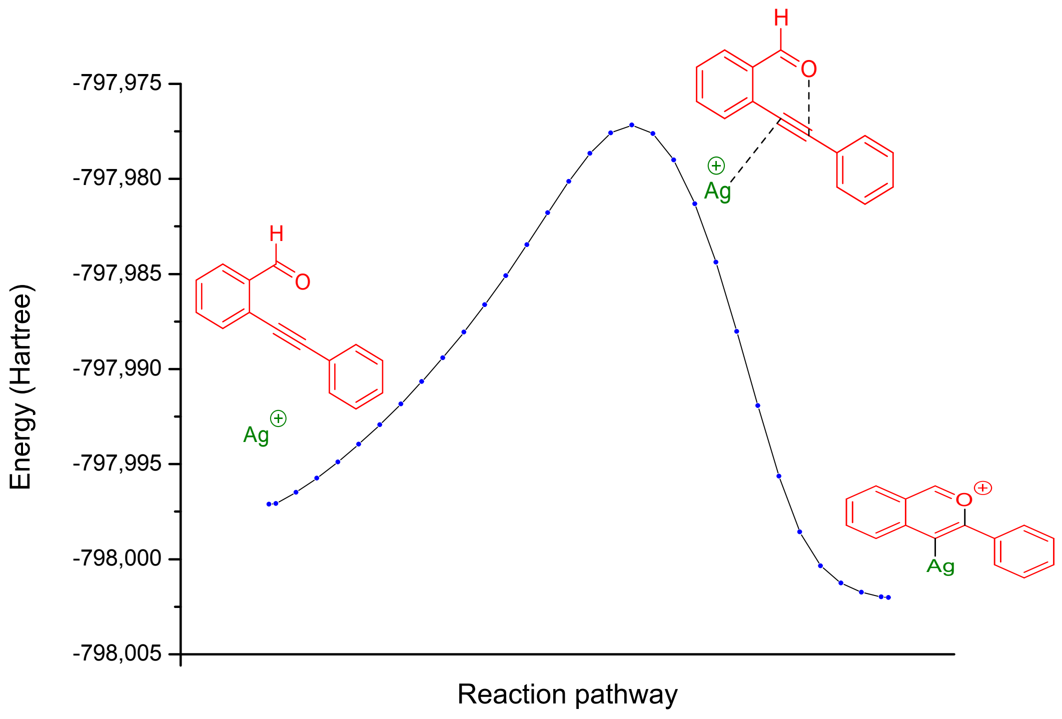

Figure S25. Figure Intrinsic Reaction Coordinate – Proposal 4 (5-membered ring pathway – second step)

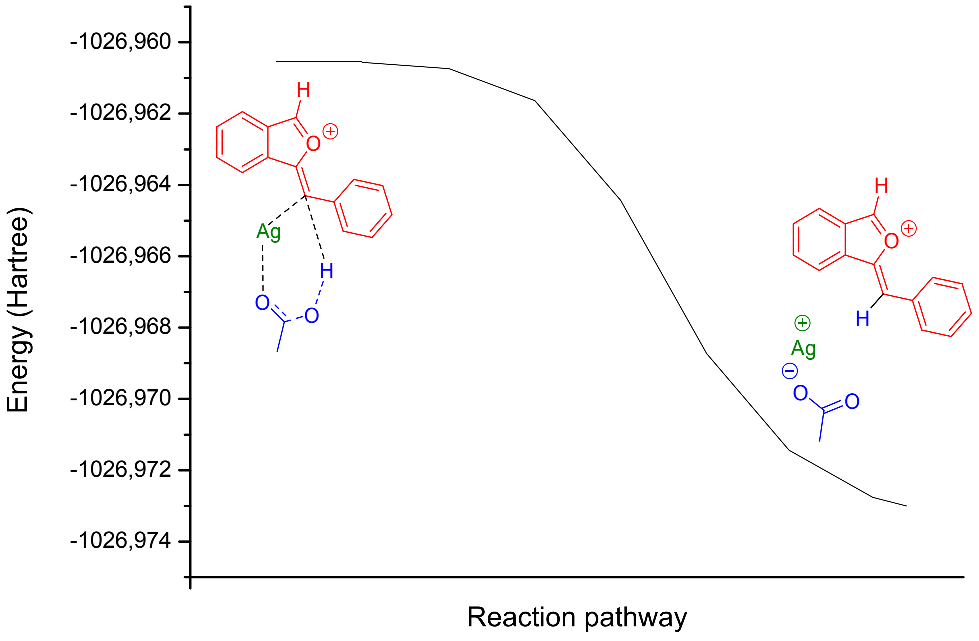

Figure S26. Figure Intrinsic Reaction Coordinate – Proposal 4 (6-membered ring pathway – second step)

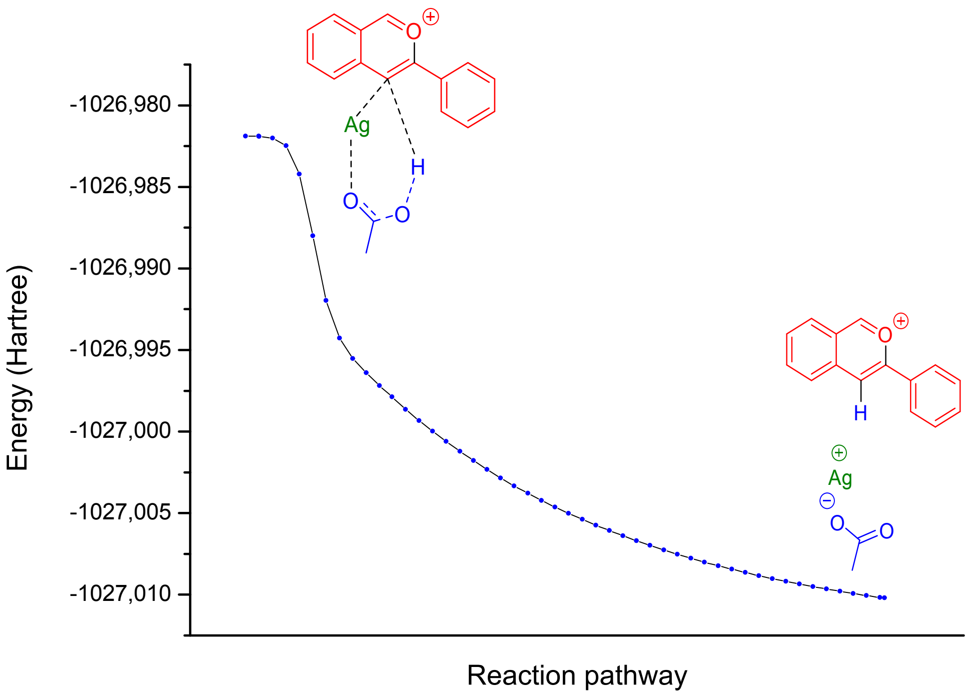

Figure S27. Figure Intrinsic Reaction Coordinate – Proposal 4 (5-membered ring pathway – third step)

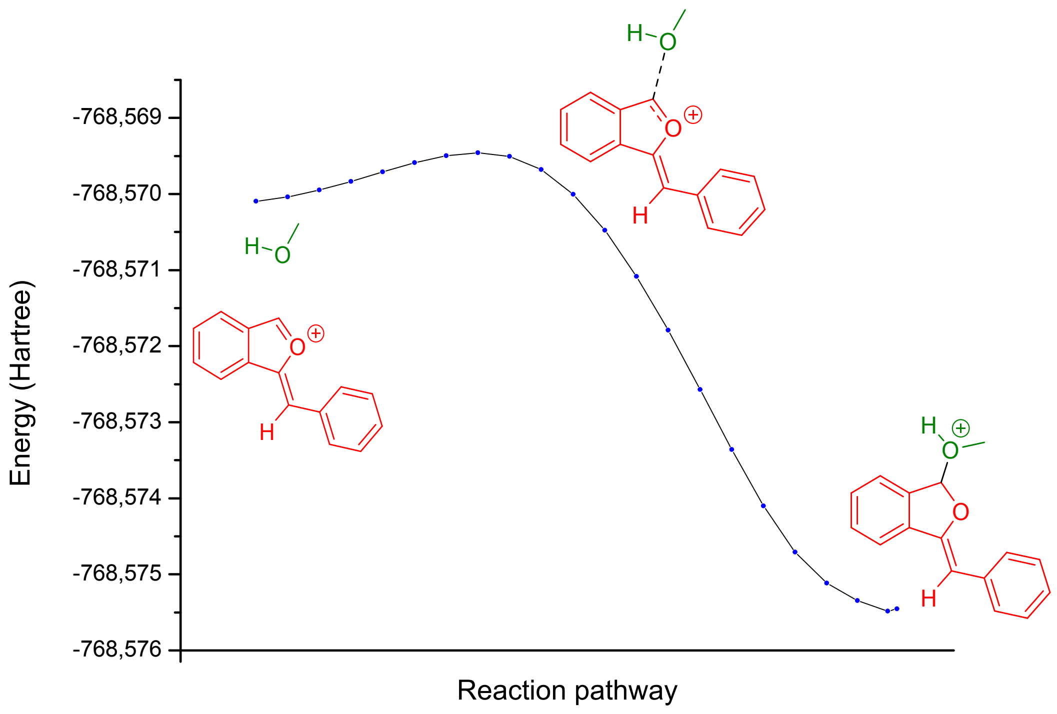

Figure S28. Figure Intrinsic Reaction Coordinate – Proposal 4 (6-membered ring pathway – third step)

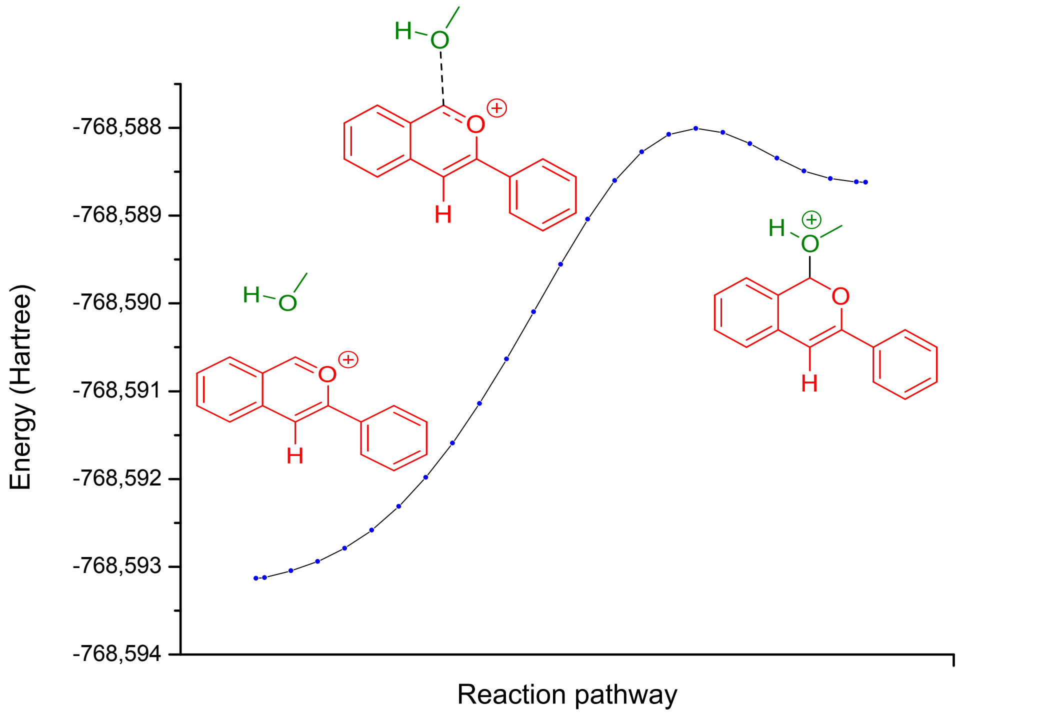

Figure S29. Scan data for the proton transfer – Proposal 4 (5-membered ring pathway – fourth step)

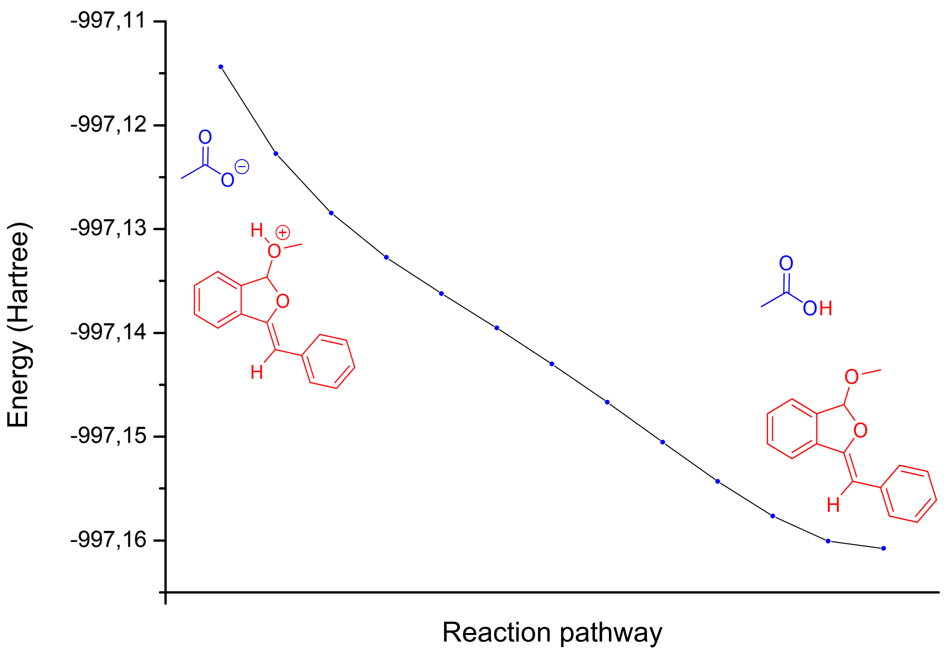

Figure S30. Scan data for the proton transfer– Proposal 4 (6-membered ring pathway – fourth step)

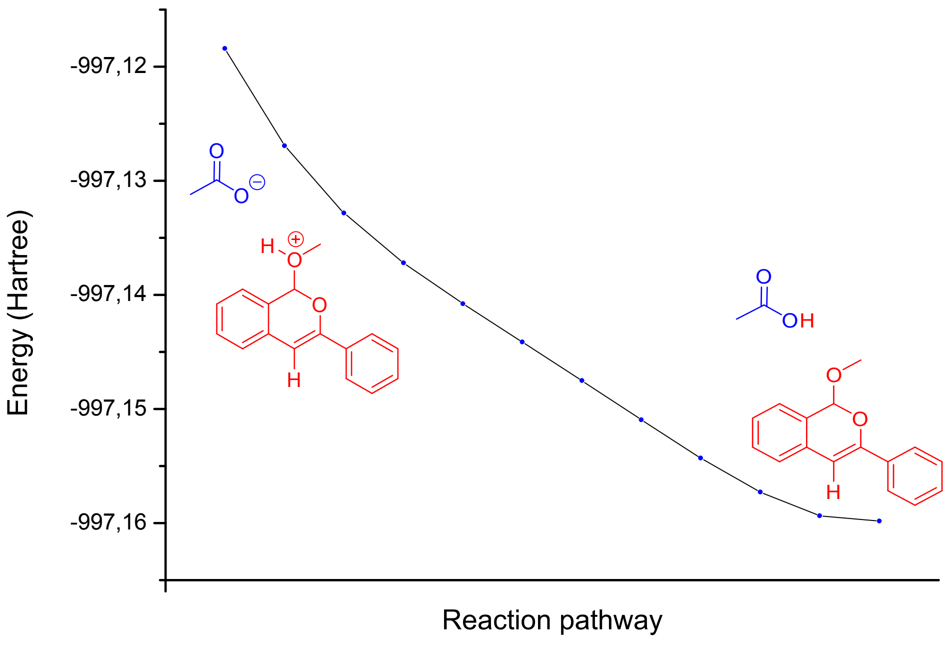

Figure S31. Figure Intrinsic Reaction Coordinate – Proposal 5 (5-membered ring pathway)

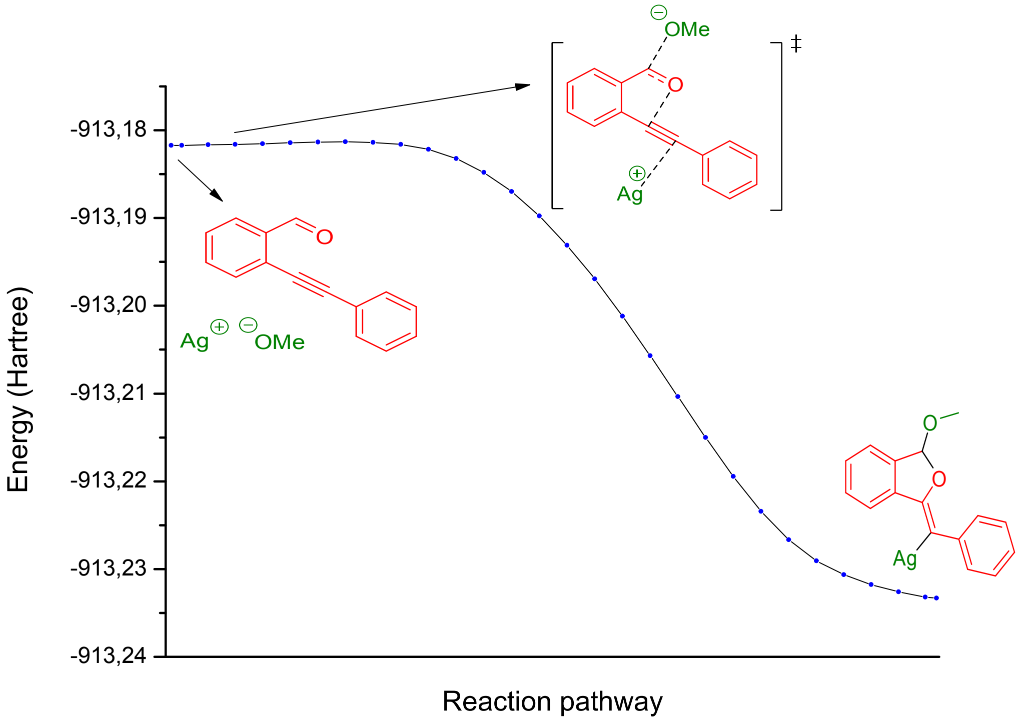

Figure S32. Figure Intrinsic Reaction Coordinate – Proposal 5 (6-membered ring pathway)

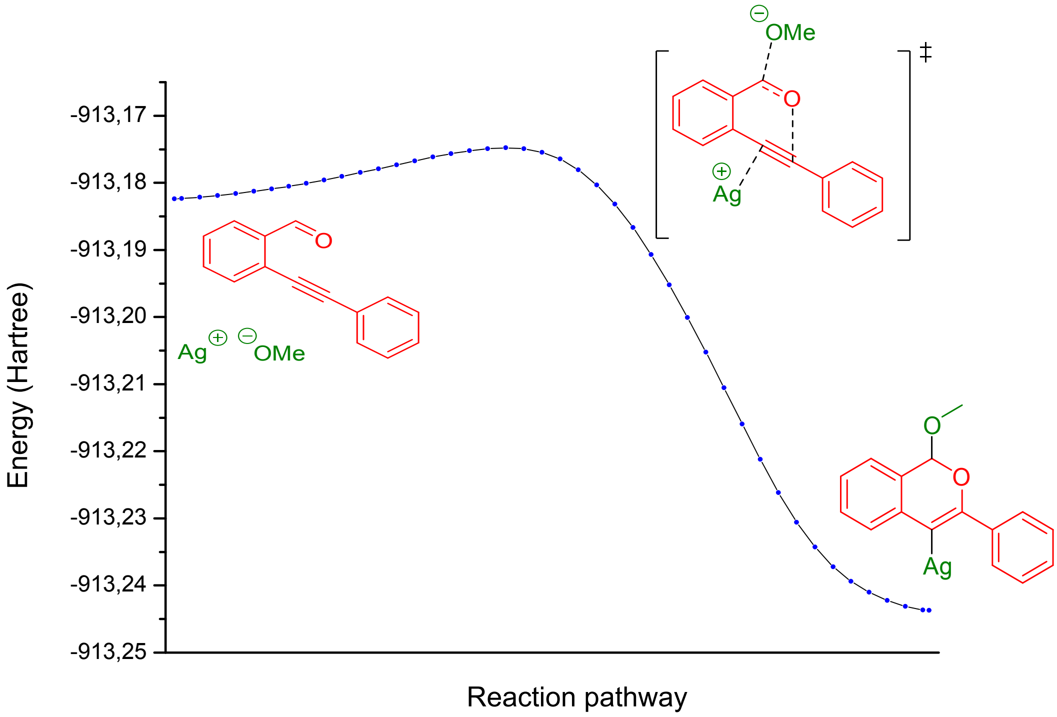

14. ELECTRONIC ENERGIES (E), ENTHALPIES (H) AND GIBBS FREE ENERGIES (G) OF ALL OPTIMIZED STRUCTURES

Table S2. Electronic energies (ΔE), enthalpies (ΔH) and Gibbs free energies (ΔG) variation for proposals 1-3 and 5. Values in Hartree.

Proposal		E	H	G
Proposal 1 - 6-membered product	Molecular complex (reagents)	-1142.240061	-1141.899069	-1142.00074
	Transition state	-1142.186657	-1141.847777	-1141.939845
	Molecular complex (products)	-1142.234547	-1141.89203	-1141.981725
Proposal 2 - 5-membered product	Molecular complex (reagents)	-881.4198943	-881.1276393	-881.2114493
	Transition state	-881.3789349	-881.0908639	-881.1708469
	Molecular complex (products)	-881.41128	-881.118567	-881.196902
Proposal 2 - 6-membered product	Molecular complex (reagents)	-881.4217037	-881.1296937	-881.2144297
	Transition state	-881.383582	-881.094914	-881.175068
	Molecular complex (products)	-881.4342954	-881.1407674	-881.2183314
Proposal 3 - 5-membered product	Molecular complex (reagents)	-1026.547618	-1026.265096	-1026.355384
	Transition state	-1026.517339	-1026.236577	-1026.322608
	Molecular complex (products)	-1026.528454	-1026.245591	-1026.330821
Proposal 3 - 6-membered product	Molecular complex (reagents)	-1026.547542	-1026.264889	-1026.354419
	Transition state	-1026.52083	-1026.239968	-1026.326004
	Molecular complex (products)	-1026.546217	-1026.262764	-1026.347183
Proposal 5 - 5-membered product	Molecular complex (reagents)	-913.182839	-912.919681	-912.976144
	Transition state	-913.1813094	-912.9191424	-912.9743024
	Molecular complex (products)	-913.2367165	-912.9714935	-913.0252725
Proposal 5 - 6-membered product	Molecular complex (reagents)	-913.1828377	-912.9196787	-912.9760807
	Transition state	-913.1747994	-912.9126574	-912.9667404
	Molecular complex (products)	-913.2460622	-912.9803212	-913.0329802

Table S3. Electronic energies (ΔE), enthalpies (ΔH) and Gibbs free energies (ΔG) variation for each step of the formation of the 5-membered ring product through proposal 4. Values in Hartree.

Proposal		E	H	G
Step 1	Molecular complex (reagents)	-798.0024119	-797.7776069	-797.8493059
	Transition state	-797.9721527	-797.7492607	-797.8170297
	Molecular complex (products)	-797.9827763	-797.7579613	-797.8257113
Step 2	Molecular complex (reagents)	-1026.972084	-1026.676413	-1026.761534
	Transition state	-1026.960534	-1026.668964	-1026.75084
	Molecular complex (products)	-1026.994575	-1026.698553	-1026.785059
Step 3	Molecular complex (reagents)	-768.5701737	-768.2760347	-768.3499777
	Transition state	-768.5694585	-768.2761655	-768.3468725
	Molecular complex (products)	-768.5762014	-768.2811424	-768.3507624
Step 4	Protonated product	-768.5762014	-768.2811424	-768.3507624
	Acetate	-228.4882104	-228.4329444	-228.4698114
	Total (reagents)	-997.0644118	-996.7140868	-996.8205738
	Product	-768.1759101	-767.8933901	-767.9622101
	Acetic acid	-228.9667052	-228.8994832	-228.9347892
	Total (products)	-997.1426153	-996.7928733	-996.8969993

Table S4. Electronic energies (ΔE), enthalpies (ΔH) and Gibbs free energies (ΔG) variation for each step of the formation of the 6-membered ring product through proposal 4. Values in Hartree.

Proposal		E	H	G
Step 1	Molecular complex (reagents)	-798.002413	-797.77761	-797.849336
	Transition state	-797.9771758	-797.7539528	-797.8218488
	Molecular complex (products)	-798.0028293	-797.7772173	-797.8438833
Step 2	Molecular complex (reagents)	-1026.992125	-1026.695852	-1026.782057
	Transition state	-1026.981888	-1026.689087	-1026.769563
	Molecular complex (products)	-1027.014372	-1026.717322	-1026.801505
Step 3	Molecular complex (reagents)	-768.5947764	-768.3001224	-768.3722904
	Transition state	-768.5880074	-768.2942694	-768.3635184
	Molecular complex (products)	-768.5888923	-768.2935793	-768.3631233
Step 4	Protonated product	-768.5888923	-768.2935793	-768.3631233
	Acetate	-228.4882104	-228.4329444	-228.4698114
	Total (reagents)	-997.0771027	-996.7265237	-996.8329347
	Product	-768.1816726	-767.8988526	-767.9661926
	Acetic acid	-228.9667052	-228.8994832	-228.9347892
	Total (products)	-997.1483778	-996.7983358	-996.9009818

15. ELECTRONIC ENERGIES (ΔE), ENTHALPIES (ΔH) AND GIBBS FREE ENERGIES (ΔG) VARIATION ALONG THE REACTION PATHWAY

Table S5. Electronic energies (ΔE), enthalpies (ΔH) and Gibbs free energies (ΔG) variation for proposals 1-3 and 5. Values in kcal mol^{-1}.

Proposal		ΔE	ΔH	ΔG
Proposal 1 - 6-membered product	Molecular complex (reagents)	0.00	0.00	0.00
	Transition state	33.51	32.19	38.21
	Molecular complex (products)	3.46	4.42	11.93
Proposal 2 - 5-membered product	Molecular complex (reagents)	0.00	0.00	0.00
	Transition state	25.70	23.08	25.48
	Molecular complex (products)	5.41	5.69	9.13
Proposal 2 - 6-membered product	Molecular complex (reagents)	0.00	0.00	0.00
	Transition state	23.92	21.82	24.70
	Molecular complex (products)	-7.90	-6.95	-2.45
Proposal 3 - 5-membered product	Molecular complex (reagents)	0.00	0.00	0.00
	Transition state	19.00	17.90	20.57
	Molecular complex (products)	12.03	12.24	15.41
Proposal 3 - 6-membered product	Molecular complex (reagents)	0.00	0.00	0.00
	Transition state	16.76	15.64	17.83
	Molecular complex (products)	0.83	1.33	4.54
Proposal 5 - 5-membered product	Molecular complex (reagents)	0.00	0.00	0.00
	Transition state	0.96	0.34	1.16
	Molecular complex (products)	-33.81	-32.51	-30.83
Proposal 5 - 6-membered product	Molecular complex (reagents)	0.00	0.00	0.00
	Transition state	5.04	4.41	5.86
	Molecular complex (products)	-39.67	-38.05	-35.70

Table S6. Electronic energies (ΔE), enthalpies (ΔH) and Gibbs free energies (ΔG) variation for each step of the formation of the 5-membered ring product through proposal 4. Values in kcal mol^{-1}.

Proposal		ΔE	ΔH	ΔG
Step 1	Molecular complex (reagents)	0.00	0.00	0.00
	Transition state	18.99	17.79	20.25
	Molecular complex (products)	12.32	12.33	14.81
Step 2	Molecular complex (reagents)	0.00	0.00	0.00
	Transition state	7.25	4.67	6.71
	Molecular complex (products)	-14.11	-13.89	-14.76
Step 3	Molecular complex (reagents)	0.00	0.00	0.00
	Transition state	0.45	-0.08	1.95
	Molecular complex (products)	-3.78	-3.21	-0.49
Step 4	Reagents	0.00	0.00	0.00
	Products	-49.07	-49.44	-47.96

Table S7. Electronic energies (ΔE), enthalpies (ΔH) and Gibbs free energies (ΔG) variation for each step of the formation of the 6-membered ring product through proposal 4. Values in kcal mol^{-1}.

Proposal		ΔE	ΔH	ΔG
Step 1	Molecular complex (reagents)	0.00	0.00	0.00
	Transition state	15.84	14.85	17.25
	Molecular complex (products)	-0.26	0.25	3.42
Step 2	Molecular complex (reagents)	0.00	0.00	0.00
	Transition state	6.42	4.25	7.84
	Molecular complex (products)	-13.96	-13.47	-12.20
Step 3	Molecular complex (reagents)	0.00	0.00	0.00
	Transition state	4.25	3.67	5.50
	Molecular complex (products)	3.69	4.11	5.75
Step 4	Reagents	0.00	0.00	0.00
	Products	-44.73	-45.06	-42.70

16. COORDINATES OF OPTIMIZED STATIONARY POINTS

- Molecular complex (reagents) - Proposal 1 (6-membered ring pathway)

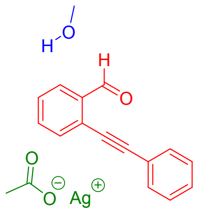

Symbolic Z-matrix:
Charge = 0 Multiplicity = 1

C	3.11015	-3.58276	-0.86631
C	1.75594	-3.22963	-0.79689
C	1.32548	-2.17897	0.04551
C	2.28362	-1.50775	0.85257
C	3.63952	-1.86756	0.76172
C	4.0593	-2.90111	-0.08753
H	3.42192	-4.3851	-1.52786
H	1.02318	-3.75531	-1.40223
H	4.36571	-1.33385	1.37046
H	5.107	-3.17653	-0.13735
C	1.95196	-0.4041	1.79027
H	2.82808	0.10344	2.22232
O	0.79539	-0.08704	2.12874
O	2.20654	1.25807	-0.08182
H	1.93596	2.22364	0.17248
C	3.27078	1.20111	-1.06763
H	3.23273	0.22662	-1.56624
H	3.15097	1.98711	-1.82513
H	4.25069	1.31146	-0.58449
C	-0.08856	-1.88631	0.08774
C	-1.31033	-1.74628	0.06724
C	-2.731	-1.51306	0.07895
C	-3.21836	-0.20095	0.29018
C	-3.63549	-2.58089	-0.12579
C	-4.59942	0.0385	0.30345
H	-2.50452	0.61211	0.427
C	-5.01387	-2.3342	-0.11256
H	-3.25415	-3.58624	-0.2899
C	-5.50012	-1.0271	0.10257
H	-4.96934	1.04903	0.4643
H	-5.7099	-3.15418	-0.2701
H	-6.57018	-0.84227	0.10849
C	0.12074	3.82329	-0.03442
O	1.31406	3.55007	0.38915
O	-0.69771	2.94431	-0.51742
C	-0.34864	5.26456	0.06499
H	-0.39117	5.5608	1.11872
H	0.37445	5.92049	-0.43237
H	-1.33628	5.38503	-0.38803
Ag	-0.09216	0.73042	-0.44539

- Transition state - Proposal 1 (6-membered ring pathway)

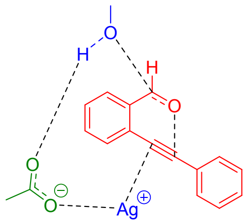

Symbolic Z-matrix:
Charge = 0 Multiplicity = 1

C	4.74833	-1.51756	-0.30687
C	3.44151	-1.8686	-0.67629
C	2.33646	-1.25801	-0.05394
C	2.54645	-0.26785	0.93804
C	3.85706	0.07424	1.29923
C	4.95912	-0.5446	0.68499
H	5.59505	-1.99761	-0.78672
H	3.26682	-2.62177	-1.43881
H	4.01477	0.83121	2.06501
H	5.96838	-0.26868	0.97291
C	1.37853	0.4491	1.61917
H	1.75776	1.04565	2.46765
O	0.26769	-0.2588	1.89862
O	0.95318	1.59867	0.61614
H	-0.39345	2.03922	0.82994
C	1.9331	2.66157	0.41471
H	2.81374	2.27287	-0.10465
H	1.46301	3.43439	-0.19937
H	2.23199	3.08805	1.37891
C	0.9592	-1.62758	-0.38424
C	-0.14876	-1.45709	0.2162
C	-1.57429	-1.69391	0.38256
C	-2.36288	-0.92402	1.26199
C	-2.18035	-2.71304	-0.38948
C	-3.73948	-1.17115	1.36302
H	-1.87056	-0.15803	1.84939
C	-3.55668	-2.94996	-0.28389
H	-1.57121	-3.30704	-1.06413
C	-4.34224	-2.17967	0.59211
H	-4.34205	-0.57532	2.04169
H	-4.01483	-3.7326	-0.88003
H	-5.40855	-2.36525	0.67238
C	-2.24723	2.15446	-0.00599
O	-1.38739	2.36319	0.98698
O	-1.90493	1.65834	-1.11607
C	-3.64924	2.61912	0.25673
H	-4.33112	2.19995	-0.48323
H	-3.9583	2.34987	1.26793
H	-3.67477	3.71344	0.17711
Ag	0.10369	0.52017	-1.47701

- Molecular complex (products) - Proposal 1 (6-membered ring pathway)

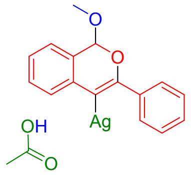

Symbolic Z-matrix:

Charge = 0 Multiplicity = 1

C	4.73703	-1.47588	-0.35155
C	3.43204	-1.80826	-0.72999
C	2.30005	-1.23183	-0.08957
C	2.55276	-0.296	0.93119
C	3.85292	0.04355	1.32596
C	4.95839	-0.549	0.68469
H	5.58635	-1.93012	-0.85941
H	3.2537	-2.51971	-1.52547
H	4.00672	0.76345	2.12745
H	5.96612	-0.28241	0.97854
C	1.35363	0.39683	1.51899
H	1.52394	0.70797	2.61014
O	0.18371	-0.51087	1.59802
O	0.94663	1.50507	0.74389
H	-0.47189	1.98936	0.96201
C	1.90438	2.60586	0.51389
H	2.75743	2.2498	-0.06498
H	1.35518	3.35454	-0.06101
H	2.23146	3.02686	1.46832
C	0.91718	-1.67809	-0.57427
C	-0.01676	-1.21224	0.56418
C	-1.51281	-1.68895	0.42027
C	-2.40321	-0.94792	1.22335
C	-2.05668	-2.77013	-0.33924
C	-3.77152	-1.24692	1.30712
H	-2.01407	-0.12155	1.80442
C	-3.44136	-3.06606	-0.26003
H	-1.41376	-3.37884	-0.9678
C	-4.29645	-2.31731	0.57085
H	-4.40692	-0.64871	1.95281
H	-3.84449	-3.89878	-0.83112
H	-5.35642	-2.56319	0.63938
C	-2.21015	2.30188	0.00472
O	-1.39575	2.35976	1.06838
O	-1.81899	1.85879	-1.10464
C	-3.59261	2.80622	0.2637
H	-4.06672	2.17114	1.01441
H	-3.54287	3.82231	0.66613
H	-4.17566	2.79199	-0.6566
Ag	0.06145	0.53999	-1.4827

- Molecular complex (reagents) - Proposal 2 (5-membered ring pathway)

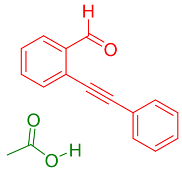

Symbolic Z-matrix:

Charge = 0 Multiplicity = 1

C	3.983	0.71822	0.42987
C	2.57401	0.67544	0.43702
C	1.95227	-0.5279	0.15654
C	2.67311	-1.66808	-0.13056
C	4.04999	-1.64385	-0.14713
C	4.72702	-0.41698	0.14159
H	4.48372	1.65482	0.65263
H	2.01119	1.56117	0.67659
H	4.62771	-2.54436	-0.37082
H	5.80817	-0.3799	0.14776
C	1.82828	-2.9337	-0.43641
H	2.52576	-3.83904	-0.68413
O	0.65699	-2.99276	-0.43232
C	0.467	-0.26341	0.23266
C	-0.7557	-0.38364	0.21404
C	-2.14649	-0.5935	0.17934
C	-3.11965	0.38224	0.2
C	-2.47834	-1.9732	0.07986
C	-4.46632	0.01635	0.12964
H	-2.84842	1.43622	0.26763
C	-3.82422	-2.32678	0.02083
H	-1.66731	-2.70548	0.05001
C	-4.82199	-1.3345	0.04176
H	-5.23517	0.78177	0.14823
H	-4.10491	-3.38049	-0.05325
H	-5.87191	-1.61522	-0.00711
C	-0.28253	3.28446	-0.37452
O	-0.30261	2.81702	-1.50524
O	-0.36568	2.46449	0.75611
C	-0.15909	4.6934	0.0558
H	-1.02388	4.97673	0.65858
H	0.73591	4.82178	0.67021
H	-0.09757	5.33432	-0.8214
H	-0.375	1.82026	0.55459

- Transition state - Proposal 2 (5-membered ring pathway)

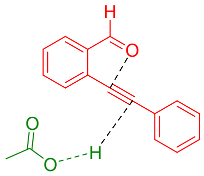

Symbolic Z-matrix:

Charge = 0 Multiplicity = 1

C	3.94641	0.85932	-0.41974
C	2.55031	0.76049	-0.42703
C	1.96705	-0.48047	-0.10979
C	2.80319	-1.58279	0.19701
C	4.20731	-1.48275	0.20104
C	4.77564	-0.24758	-0.109
H	4.40979	1.80996	-0.66299
H	1.9318	1.61555	-0.68044
H	4.81949	-2.34565	0.44056
H	5.85276	-0.12707	-0.11615
C	1.98276	-2.72855	0.47396
H	2.34828	-3.71442	0.75438
O	0.72207	-2.50825	0.37788
C	0.55293	-0.78719	-0.06657
C	-0.62303	-0.25724	-0.21308
C	-2.00513	-0.72558	-0.15634
C	-3.03348	0.22376	0.04647
C	-2.35747	-2.08905	-0.31857
C	-4.37678	-0.17382	0.0963
H	-2.77168	1.27079	0.17655
C	-3.70146	-2.48079	-0.28645
H	-1.57712	-2.82717	-0.47061
C	-4.71463	-1.52664	-0.07724
H	-5.15531	0.56469	0.25783
H	-3.96288	-3.52595	-0.4182
H	-5.75494	-1.8349	-0.05221
C	-0.58009	3.22696	0.26551
O	-0.86584	2.84791	1.42023
O	-0.41863	2.36951	-0.76618
C	-0.40292	4.66795	-0.13447
H	0.5127	4.78144	-0.72074
H	-1.24575	4.96868	-0.76609
H	-0.36746	5.31081	0.74583
H	-0.48408	1.32374	-0.5151

- Molecular complex (products) - Proposal 2 (5-membered ring pathway)

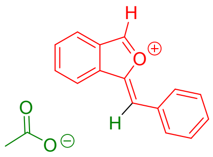

Symbolic Z-matrix:

Charge = 0 Multiplicity = 1

C	3.914	0.70442	0.42987
C	2.51881	0.66164	0.43702
C	1.88327	-0.5693	0.14274
C	2.70071	-1.70948	-0.14436
C	4.11899	-1.65765	-0.14713
C	4.71322	-0.44458	0.14159
H	4.41472	1.64102	0.65263
H	1.92839	1.54737	0.66279
H	4.69671	-2.54436	-0.37082
H	5.79437	-0.3523	0.14776
C	1.82828	-2.7681	-0.39501
H	2.01516	-3.81144	-0.65653
O	0.54659	-2.37176	-0.28052
C	0.4808	-1.00861	0.05326
C	-0.6453	-0.16284	0.25544
C	-2.04989	-0.6487	0.16554
C	-3.06445	0.35464	0.2
C	-2.46454	-2.0008	0.07986
C	-4.42492	0.01635	0.12964
H	-2.76562	1.39482	0.28143
C	-3.82422	-2.34058	0.02083
H	-1.72251	-2.78828	0.06381
C	-4.80819	-1.3345	0.04176
H	-5.17997	0.79557	0.14823
H	-4.11871	-3.38049	-0.03945
H	-5.85811	-1.60142	-0.00711
C	-0.28253	3.21547	-0.31932
O	-0.30261	2.83082	-1.51904
O	-0.36568	2.38169	0.71471
C	-0.15909	4.6796	0.0558
H	-1.02388	4.97673	0.65858
H	0.73591	4.82178	0.67021
H	-0.09757	5.30673	-0.8352
H	-0.4854	1.17166	0.48559

- Molecular complex (reagents) - Proposal 2 (6-membered ring pathway)

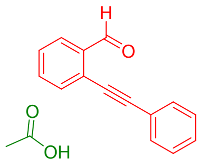

Symbolic Z-matrix:

Charge = 0 Multiplicity = 1

C	3.95149	1.35835	-1.20946
C	2.60881	0.94489	-1.1913
C	2.20484	-0.17275	-0.42812
C	3.18763	-0.88576	0.31013
C	4.52859	-0.45455	0.28699
C	4.91759	0.66004	-0.46718
H	4.23819	2.22212	-1.79806
H	1.86919	1.48748	-1.77044
H	5.2697	-1.00198	0.86274
H	5.95508	0.9789	-0.47629
C	2.88904	-2.07733	1.13452
H	3.75838	-2.48084	1.67862
O	1.77502	-2.61992	1.24713
C	0.81158	-0.53289	-0.42594
C	-0.39201	-0.75076	-0.46075
C	-1.79784	-1.06367	-0.47841
C	-2.28417	-2.15837	0.269
C	-2.70526	-0.27663	-1.22555
C	-3.65461	-2.45859	0.27023
H	-1.59203	-2.76393	0.84483
C	-4.0714	-0.58454	-1.22103

H	-2.33561	0.55709	-1.81348
C	-4.55171	-1.67368	-0.47221
H	-4.01909	-3.30034	0.84921
H	-4.75923	0.02273	-1.79959
H	-5.61127	-1.90694	-0.4665
C	-1.59555	2.64574	0.90246
O	-1.69221	1.82845	1.82698
O	-0.8395	2.3641	-0.21262
C	-2.23236	4.00116	0.85201
H	-2.76905	4.20025	1.77946
H	-1.46707	4.76642	0.69505
H	-2.92764	4.04747	0.00848
H	-0.44411	1.46663	-0.1584

- Transition state - Proposal 2 (6-membered ring pathway)

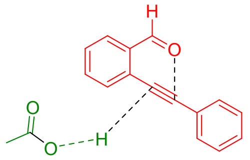

Symbolic Z-matrix:
Charge = 0 Multiplicity = 1

C	-4.28816	1.11974	0.20543
C	-2.89406	1.22629	0.26591
C	-2.08951	0.07717	0.1407
C	-2.72231	-1.18251	-0.0474
C	-4.13013	-1.28128	-0.10814
C	-4.91237	-0.1333	0.01787
H	-4.8989	2.01118	0.30345
H	-2.4205	2.19243	0.40896
H	-4.59024	-2.25536	-0.25208
H	-5.99348	-0.19899	-0.02745
C	-1.94909	-2.40369	-0.17617
H	-2.50129	-3.33901	-0.31664
O	-0.68235	-2.47435	-0.13189
C	-0.63664	0.14244	0.19262
C	0.22159	-0.80848	0.10407
C	1.62101	-1.20077	0.13196
C	2.09863	-2.38898	-0.45594
C	2.53124	-0.31232	0.75103
C	3.46814	-2.68398	-0.42238
H	1.40407	-3.07043	-0.93041
C	3.89882	-0.6122	0.77331
H	2.16406	0.59904	1.21383
C	4.37265	-1.79933	0.18872
H	3.82892	-3.60121	-0.87595
H	4.59059	0.07488	1.24917
H	5.43225	-2.03191	0.2101
C	1.28936	3.12799	-0.40071
O	1.55773	2.5401	-1.46691
O	0.47174	2.60286	0.54013
C	1.82882	4.47917	-0.01632
H	2.4531	4.88078	-0.81489
H	0.99967	5.16348	0.18575
H	2.41747	4.39029	0.90195
H	0.08946	1.6403	0.34631

- Molecular complex (products) - Proposal 2 (6-membered ring pathway)

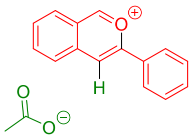

Symbolic Z-matrix:
Charge = 0 Multiplicity = 1

C	4.27411	1.15234	-0.21129
C	2.88421	1.24347	-0.27032
C	2.0632	0.08384	-0.14175
C	2.73536	-1.16561	0.04967
C	4.15895	-1.25395	0.10832
C	4.9177	-0.10571	-0.02199
H	4.87813	2.04805	-0.31206
H	2.39341	2.2007	-0.41532
H	4.62635	-2.22434	0.25868
H	5.99934	-0.15349	0.0215
C	1.94119	-2.31176	0.17497
H	2.33512	-3.327	0.32293
O	0.61005	-2.25212	0.10891
C	0.58999	0.23324	-0.21412
C	-0.0458	-1.05436	-0.07956
C	-1.56218	-1.2421	-0.13014
C	-2.07846	-2.41018	0.46305
C	-2.46639	-0.33855	-0.74743
C	-3.45091	-2.69945	0.42243
H	-1.4057	-3.10435	0.95323
C	-3.84688	-0.6326	-0.77675
H	-2.09373	0.5722	-1.21005
C	-4.34455	-1.81424	-0.20115
H	-3.8119	-3.61143	0.88525
H	-4.53181	0.05701	-1.26372
H	-5.40257	-2.04377	-0.23384
C	-1.32746	3.08161	0.39272
O	-1.59756	2.50703	1.47957
O	-0.50274	2.57178	-0.52182
C	-1.9198	4.42459	0.02111
H	-2.56206	4.79836	0.8216
H	-1.11827	5.14211	-0.17532
H	-2.50448	4.32133	-0.90086
H	-0.01847	1.50261	-0.35692

- Molecular complex (reagents) - Proposal 3 (5-membered ring pathway)

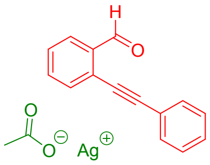

Symbolic Z-matrix:

Charge = 0 Multiplicity = 1

C	-2.84187	2.8171	-0.0321
C	-1.85727	1.81114	-0.03452
C	-0.51258	2.18128	-0.04524
C	-0.14782	3.52822	-0.05435
C	-1.11457	4.5268	-0.05209
C	-2.48142	4.15738	-0.0396
H	-3.88604	2.52839	-0.02433
H	-2.15521	0.76403	-0.0291
H	-0.81945	5.57462	-0.06011
H	-3.25172	4.92639	-0.03658
C	1.34495	3.82629	-0.07993
H	1.58585	4.93752	-0.10892
O	2.19613	2.96833	-0.07677
C	0.35191	0.97537	-0.0508
C	1.27485	0.13067	-0.049
C	2.52596	-0.55002	-0.01619
C	2.73349	-1.80438	-0.5805
C	3.58565	0.17403	0.57425
C	4.01775	-2.36666	-0.55611
H	1.91515	-2.34194	-1.04974
C	4.86352	-0.40087	0.60347
H	3.39852	1.15972	0.98351
C	5.0835	-1.67525	0.03963
H	4.18654	-3.34753	-0.99347
H	5.68714	0.13394	1.06647
H	6.07036	-2.11173	0.06613
C	-3.28865	-2.29919	0.09122
O	-3.39795	-1.03425	-0.01425
O	-2.12624	-2.91091	0.11228
C	-4.51141	-3.18813	0.20533
H	-4.50331	-3.92866	-0.60078
H	-4.47909	-3.73374	1.15398
H	-5.42895	-2.59985	0.15396
Ag	-0.55124	-1.37927	-0.00645

- Transition state - Proposal 3 (5-membered ring pathway)

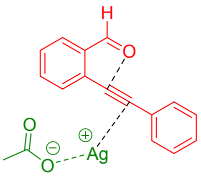

Symbolic Z-matrix:

Charge = 0 Multiplicity = 1

C	-2.79635	-2.79006	0.02377
C	-1.81244	-1.79051	0.02646
C	-0.46044	-2.18092	0.04413
C	-0.14442	-3.56309	0.05865
C	-1.13175	-4.56237	0.05616
C	-2.47044	-4.16556	0.03748
H	-3.84068	-2.49501	0.01075
H	-2.11111	-0.74367	0.01518
H	-0.85014	-5.60996	0.06861
H	-3.2615	-4.90675	0.03413
C	1.28449	-3.74646	0.08966
H	1.7815	-4.71526	0.12446
O	1.96457	-2.66799	0.08442
C	0.69442	-1.30065	0.05143
C	1.16208	-0.09349	0.05466
C	2.50511	0.49568	0.02063
C	2.72614	1.78053	0.56613
C	3.59363	-0.18726	-0.57445
C	4.00302	2.35757	0.54688
H	1.89337	2.31956	1.01187
C	4.86411	0.40228	-0.61272
H	3.43542	-1.16743	-1.00876
C	5.0763	1.67168	-0.04625
H	4.15704	3.34013	0.98063
H	5.68802	-0.12669	-1.0809
H	6.06292	2.12277	-0.07428
C	-3.29309	2.29459	-0.09579
O	-3.4107	1.02764	-0.02154
O	-2.13057	2.8995	-0.09251
C	-4.51499	3.18632	-0.19723
H	-4.52235	3.89489	0.63721
H	-4.46727	3.76801	-1.1234
H	-5.43239	2.59563	-0.18385
Ag	-0.54535	1.37874	0.01123

- Molecular complex (products) - Proposal 3 (5-membered ring pathway)

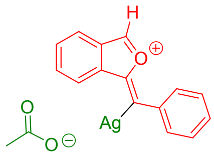

Symbolic Z-matrix:

Charge = 0 Multiplicity = 1

C	-2.77407	2.7719	-0.0321
C	-1.78947	1.77724	-0.03452
C	-0.43348	2.18128	-0.04524
C	-0.14782	3.58472	-0.05435
C	-1.14847	4.5833	-0.05209
C	-2.47012	4.16868	-0.0396
H	-3.81824	2.47189	-0.02433
H	-2.08741	0.73013	-0.0291
H	-0.87595	5.63112	-0.06011
H	-3.27432	4.89249	-0.03658
C	1.24325	3.70199	-0.07993
H	1.89095	4.58722	-0.10892
O	1.82323	2.49373	-0.07677
C	0.89431	1.49517	-0.0508
C	1.09405	0.07417	-0.0603
C	2.49206	-0.45962	-0.02749
C	2.72219	-1.75918	-0.5692
C	3.59695	0.19663	0.57425
C	3.99515	-2.34406	-0.55611
H	1.88125	-2.29674	-1.00454

C	4.86352	-0.40087	0.61477
H	3.45502	1.17102	1.02871
C	5.0722	-1.66395	0.03963
H	4.14133	-3.32493	-0.99347
H	5.68714	0.12264	1.08907
H	6.05905	-2.12303	0.06613
C	-3.28865	-2.29919	0.09122
O	-3.40925	-1.03425	-0.01425
O	-2.12624	-2.89961	0.11228
C	-4.51141	-3.18813	0.20533
H	-4.50331	-3.92866	-0.60078
H	-4.47909	-3.73374	1.15398
H	-5.42895	-2.59985	0.15396
Ag	-0.53994	-1.37927	-0.00645

- Molecular complex (reagents) - Proposal 3 (6-membered ring pathway)

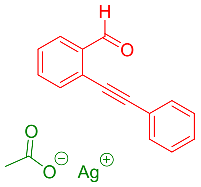

Symbolic Z-matrix:
Charge = 0 Multiplicity = 1

C	-3.69396	-2.7853	-0.03219
C	-2.44122	-2.14964	-0.0374
C	-2.37785	-0.75628	-0.00396
C	-3.56106	0.0139	0.03718
C	-4.79904	-0.6291	0.04477
C	-4.87042	-2.0337	0.00933
H	-3.74306	-3.86872	-0.05906
H	-1.52457	-2.73274	-0.06654
H	-5.70571	-0.03076	0.07659
H	-5.83591	-2.53152	0.01431
C	-3.62649	1.52781	0.07587
H	-4.69896	1.86319	0.112
O	-2.71927	2.3372	0.06166
C	-1.15371	-0.00201	0.00717
C	-0.47362	1.05861	0.00646
C	0.19935	2.3163	-0.06602
C	-0.07182	3.49477	0.66953
C	1.34491	2.22086	-0.88143
C	0.85316	4.54654	0.60642
H	-0.96954	3.56594	1.26424
C	2.26385	3.26284	-0.92095
H	1.51961	1.30482	-1.43735
C	2.0232	4.42591	-0.1712
H	0.6746	5.45561	1.17417
H	3.15594	3.173	-1.52257
H	2.741	5.23993	-0.19293
C	3.63569	-1.90266	0.27527
O	3.49716	-0.78346	0.8712
O	2.63815	-2.5072	-0.32853
C	4.97771	-2.60698	0.22541
H	5.30835	-2.68771	-0.8155
H	5.72656	-2.06158	0.80213
H	4.87689	-3.62308	0.61925
Ag	0.88686	-1.1799	-0.0822

- Transition state - Proposal 3 (6-membered ring pathway)

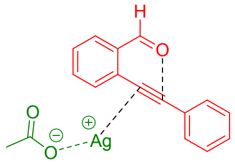

Symbolic Z-matrix:
Charge = 0 Multiplicity = 1

C	4.74833	-1.51756	-0.30687
C	3.44151	-1.8686	-0.67629
C	2.33646	-1.25801	-0.05394
C	2.54645	-0.26785	0.93804
C	3.85706	0.07424	1.29923
C	4.95912	-0.5446	0.68499
H	5.59505	-1.99761	-0.78672
H	3.26682	-2.62177	-1.43881
H	4.01477	0.83121	2.06501
H	5.96838	-0.26868	0.97291
C	1.37853	0.4491	1.61917
H	1.75776	1.04565	2.46765
O	0.26769	-0.2588	1.89862
C	0.9592	-1.62758	-0.38424
C	-0.14876	-1.45709	0.2162
C	-1.57429	-1.69391	0.38256
C	-2.36288	-0.92402	1.26199
C	-2.18035	-2.71304	-0.38948
C	-3.73948	-1.17115	1.36302
H	-1.87056	-0.15803	1.84939
C	-3.55668	-2.94996	-0.28389
H	-1.57121	-3.30704	-1.06413
C	-4.34224	-2.17967	0.59211
H	-4.34205	-0.57532	2.04169
H	-4.01483	-3.7326	-0.88003
H	-5.40855	-2.36525	0.67238
C	-2.24723	2.15446	-0.00599
O	-1.38739	2.36319	0.98698
O	-1.90493	1.65834	-1.11607
C	-3.64924	2.61912	0.25673
H	-4.33112	2.19995	-0.48323
H	-3.9583	2.34987	1.26793
H	-3.67477	3.71344	0.17711
Ag	0.10369	0.52017	-1.47701

- Molecular complex (products) - Proposal 3 (6-membered ring pathway)

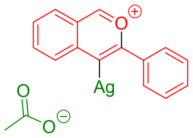

Symbolic Z-matrix:

Charge = 0 Multiplicity = 1

C	-3.67476	-2.7565	-0.03219
C	-2.43162	-2.12084	-0.0374
C	-2.32985	-0.70828	-0.00396
C	-3.55146	0.0331	0.03718
C	-4.81824	-0.6195	0.04477
C	-4.88002	-2.0049	0.00933
H	-3.72386	-3.83992	-0.05906
H	-1.51497	-2.70394	-0.06654
H	-5.72491	-0.02116	0.07659
H	-5.83591	-2.51232	0.01431
C	-3.48249	1.44141	0.06627
H	-4.37256	2.08399	0.112
O	-2.33527	2.0876	0.04246
C	-1.00011	-0.05961	-0.02163
C	-1.04962	1.34661	-0.01274
C	0.08415	2.3451	-0.06602
C	-0.08142	3.53317	0.66953
C	1.25851	2.18246	-0.84303
C	0.87236	4.55614	0.61602
H	-0.96954	3.66194	1.28344
C	2.21585	3.21484	-0.89215
H	1.40441	1.27602	-1.42775
C	2.0232	4.40671	-0.1712
H	0.713	5.46521	1.18377
H	3.10794	3.0866	-1.50337
H	2.7602	5.20153	-0.22173
C	3.63569	-1.90266	0.27527
O	3.50676	-0.78346	0.8712
O	2.63815	-2.4976	-0.32853
C	4.97771	-2.60698	0.22541
H	5.30835	-2.68771	-0.8155
H	5.72656	-2.06158	0.80213
H	4.87689	-3.62308	0.61925
Ag	0.87726	-1.1799	-0.0822

- Molecular complex (reagents) - Step 1 - Proposal 4 (5-membered ring pathway)

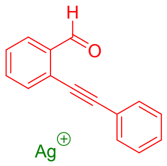

Symbolic Z-matrix:

Charge = 1 Multiplicity = 1

C	-4.14448	-0.82143	0.04766
C	-2.7431	-0.82954	0.04286
C	-2.05002	0.3802	-0.02816
C	-2.76026	1.58309	-0.09327
C	-4.1472	1.60586	-0.09044
C	-4.85222	0.37937	-0.01758
H	-4.67775	-1.76778	0.10298
H	-2.19816	-1.77339	0.0944
H	-4.6853	2.54869	-0.14337
H	-5.93542	0.37186	-0.01143
C	-1.90239	2.82537	-0.1785
H	-2.47241	3.79637	-0.25603
O	-0.6943	2.76775	-0.17266
C	-0.58923	0.22102	-0.02628
C	0.64742	0.17032	-0.02447
C	2.01575	0.5873	-0.01125
C	3.05718	-0.18743	-0.51834
C	2.26302	1.88627	0.4989
C	4.36309	0.3168	-0.52162
H	2.85036	-1.17737	-0.92798
C	3.57297	2.37878	0.50289
H	1.43527	2.47805	0.8704
C	4.62609	1.59535	-0.00769
H	5.17263	-0.29023	-0.92172
H	3.77615	3.3689	0.90351
H	5.63739	1.97929	-0.002
Ag	0.47515	-2.2147	0.08605

- Molecular complex (reagents) - Step 1 - Proposal 4 (6-membered ring pathway)

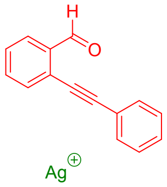

Symbolic Z-matrix:

Charge = 1 Multiplicity = 1

C	-4.32876	0.28877	0.04598
C	-2.97841	0.68199	0.00941
C	-1.98935	-0.28861	0.02508
C	-2.31106	-1.6568	0.07901
C	-3.64617	-2.03855	0.11315
C	-4.67214	-1.06204	0.09663
H	-5.10865	1.04286	0.03352
H	-2.7192	1.73504	-0.03275
H	-3.90343	-3.09543	0.15328
H	-5.70844	-1.3688	0.12241
C	-1.32544	-2.83484	0.11374
H	-1.92938	-3.79936	0.15254
O	-0.1176	-2.85417	0.1028
C	-0.57947	-0.03945	-0.0261
C	0.69521	-0.1903	-0.0451
C	2.02421	-0.72939	0.03708
C	2.59855	-1.84491	-0.61765
C	2.82103	0.19387	0.73944
C	3.99517	-1.97593	-0.58374
H	1.96163	-2.56621	-1.12166
C	4.19593	0.06012	0.74694
H	2.33451	1.02987	1.23853
C	4.79415	-1.02552	0.08064
H	4.46707	-2.81829	-1.08109
H	4.80611	0.78532	1.26908
H	5.86876	-1.12944	0.07489

Ag	0.10618	2.29184	-0.13763

- Transition state – Step 1 - Proposal 4 (5-membered ring pathway)

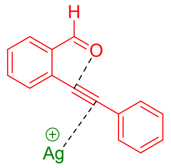

Symbolic Z-matrix:

Charge = 1 Multiplicity = 1

C	3.933	0.70822	0.42987
C	2.53401	0.66544	0.43702
C	1.90227	-0.5579	0.14654
C	2.69311	-1.69808	-0.14056
C	4.09999	-1.65385	-0.14713
C	4.71702	-0.43698	0.14159
H	4.43372	1.64482	0.65263
H	1.95119	1.55117	0.66659
H	4.67771	-2.54436	-0.37082
H	5.79817	-0.3599	0.14776
C	1.82828	-2.8137	-0.40641
H	2.15576	-3.81904	-0.66413
O	0.57699	-2.54276	-0.32232
C	0.477	-0.80341	0.10266
C	-0.6757	-0.22364	0.24404
C	-2.07649	-0.6335	0.16934
C	-3.07965	0.36224	0.2
C	-2.46834	-1.9932	0.07986
C	-4.43632	0.01635	0.12964
H	-2.78842	1.40622	0.27763
C	-3.82422	-2.33678	0.02083
H	-1.70731	-2.76548	0.06001
C	-4.81199	-1.3345	0.04176
H	-5.19517	0.79177	0.14823
H	-4.11491	-3.38049	-0.04325
H	-5.86191	-1.60522	-0.00711
Ag	-0.361	2.02064	0.61557

- Transition state – Step 1 - Proposal 4 (6-membered ring pathway)

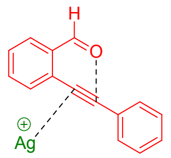

Symbolic Z-matrix:

Charge = 1 Multiplicity = 1

C	4.75283	-1.47588	-0.35945
C	3.44784	-1.81616	-0.74579
C	2.33955	-1.23973	-0.09747
C	2.54486	-0.296	0.93909
C	3.85292	0.03565	1.31806
C	4.95839	-0.549	0.67679
H	5.60215	-1.93012	-0.85941
H	3.2774	-2.53551	-1.54127
H	4.00672	0.75555	2.11955
H	5.96612	-0.28241	0.97854
C	1.36943	0.38103	1.64539
H	1.74514	0.93707	2.52324
O	0.26271	-0.34497	1.89032
C	0.96458	-1.60699	-0.43997
C	-0.14316	-1.48084	0.16918
C	-1.56021	-1.75215	0.34917
C	-2.37161	-0.96372	1.19175
C	-2.13568	-2.82543	-0.37084
C	-3.73992	-1.24692	1.30712
H	-1.90347	-0.16105	1.74912
C	-3.50456	-3.09766	-0.25213
H	-1.50856	-3.43414	-1.0152
C	-4.31226	-2.30941	0.58665
H	-4.35952	-0.64081	1.96071
H	-3.93929	-3.92248	-0.80742
H	-5.37222	-2.52369	0.67888
Ag	0.06145	0.54789	-1.4748

- Molecular complex (products) - Step 1 - Proposal 4 (5-membered ring pathway)

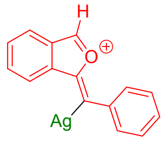

Symbolic Z-matrix:

Charge = 1 Multiplicity = 1

C	-4.07798	-0.81193	0.04766
C	-2.6766	-0.81054	0.04286
C	-2.00252	0.4277	-0.02816
C	-2.78876	1.62109	-0.09327
C	-4.2042	1.61536	-0.09044
C	-4.84272	0.38887	-0.01758
H	-4.60175	-1.75828	0.10298
H	-2.13166	-1.74489	0.0944
H	-4.7518	2.54869	-0.14337
H	-5.92592	0.33386	-0.01143
C	-1.89289	2.69237	-0.169
H	-2.07341	3.76787	-0.24653
O	-0.6278	2.26425	-0.14416
C	-0.57973	0.84802	-0.05478
C	0.57142	0.02782	-0.02447
C	1.93976	0.6253	-0.02075
C	3.01918	-0.16843	-0.50884
C	2.25352	1.90527	0.4989
C	4.33459	0.3168	-0.52162
H	2.81236	-1.16787	-0.88048
C	3.57297	2.37878	0.51239
H	1.46377	2.52555	0.9084
C	4.61659	1.59535	-0.00769
H	5.13463	-0.29973	-0.91222

H	3.78565	3.3594	0.92251
H	5.63739	1.96979	-0.002
Ag	0.47515	-2.2052	0.08605

- Molecular complex (products) - Step 1 - Proposal 4 (6-membered ring pathway)

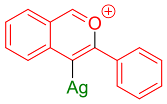

Symbolic Z-matrix:
Charge = 1 Multiplicity = 1

C	-4.29006	0.27587	0.04598
C	-2.95261	0.66909	0.00941
C	-1.89905	-0.28861	0.02508
C	-2.28526	-1.6697	0.07901
C	-3.65907	-2.06435	0.11315
C	-4.64634	-1.10074	0.09663
H	-5.06995	1.02996	0.03352
H	-2.6805	1.72214	-0.03275
H	-3.90343	-3.12123	0.15328
H	-5.69554	-1.3817	0.12241
C	-1.26094	-2.61554	0.10084
H	-1.41338	-3.70906	0.13964
O	0.0243	-2.24787	0.0899
C	-0.48917	0.17985	-0.0132
C	0.39851	-1.003	0.0452
C	1.93391	-0.85839	0.03708
C	2.62435	-1.89651	-0.61765
C	2.69203	0.14227	0.70074
C	4.02097	-1.97593	-0.59664
H	2.06483	-2.65651	-1.14746
C	4.10563	0.06012	0.72114
H	2.19261	0.95247	1.22563
C	4.76835	-0.99972	0.08064
H	4.51867	-2.79249	-1.10689
H	4.67711	0.82402	1.24328
H	5.85586	-1.06494	0.11359
Ag	0.10618	2.27894	-0.13763

- Molecular complex (reagents) - Step 2 - Proposal 4 (5-membered ring pathway)

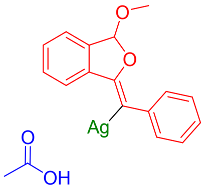

Symbolic Z-matrix:
Charge = 1 Multiplicity = 1

C	4.029	0.48702	0.27323
C	2.63611	0.41072	0.20084
C	2.04341	-0.86964	0.13199
C	2.87899	-2.03273	0.16925
C	4.2946	-1.94673	0.21604
C	4.85971	-0.67705	0.27005
H	4.50185	1.46437	0.33579
H	2.03689	1.32201	0.19844
H	4.90794	-2.8436	0.2314
H	5.92915	-0.56071	0.32721
C	2.02573	-3.15066	0.14361
H	2.25172	-4.20816	0.11959
O	0.75099	-2.77467	0.14648
C	0.66582	-1.28924	0.09171
C	-1.84937	-1.1785	0.13651
C	-2.95571	-0.37832	-0.24978
C	-2.12366	-2.46668	0.68012
C	-4.27066	-0.86452	-0.17481
H	-2.75678	0.62637	-0.61842
C	-3.43437	-2.94816	0.76836
H	-1.30816	-3.09625	1.0255
C	-4.51383	-2.15208	0.32545
H	-5.10397	-0.23507	-0.49387
H	-3.63067	-3.93874	1.17863
H	-5.52737	-2.53197	0.3923
C	-0.51172	-0.5789	-0.0409
Ag	-0.28927	1.21891	-1.41557
H	-0.54085	1.20904	1.1908
O	-0.57756	1.78667	1.80946
C	-0.49033	3.04657	1.26017
O	-0.39865	3.21583	0.04037
C	-0.5125	4.1114	2.30383
H	-1.45743	4.05968	2.8538
H	0.29823	3.94734	3.01984
H	-0.41179	5.08419	1.83758

- Molecular complex (reagents) - Step 2 - Proposal 4 (6-membered ring pathway)

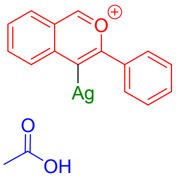

Symbolic Z-matrix:
Charge = 1 Multiplicity = 1

C	-4.22546	0.12486	0.14468
C	-2.86637	0.38223	-0.02309
C	-1.92154	-0.67117	0.05899
C	-2.42717	-2.00395	0.36489
C	-3.83388	-2.25559	0.49476
C	-4.71215	-1.2044	0.38648
H	-4.93576	0.94403	0.08347
H	-2.51068	1.39333	-0.23008
H	-4.17317	-3.28092	0.68855
H	-5.78221	-1.37379	0.47492
C	-1.49199	-3.0347	0.5264
H	-1.74638	-4.07715	0.73895
O	-0.19685	-2.79513	0.46512

C	-0.49954	-0.41332	-0.17319
C	0.3322	-1.50475	0.11046
C	1.81676	-1.55287	0.1227
C	2.49693	-2.78669	0.06355
C	2.56836	-0.36933	0.21874
C	3.89743	-2.83258	0.08587
H	1.92762	-3.71314	-0.00637
C	3.97673	-0.41051	0.24311
H	2.08273	0.59293	0.29905
C	4.63871	-1.64185	0.17039
H	4.40729	-3.78749	0.034
H	4.54067	0.51446	0.32803
H	5.72315	-1.68105	0.18968
C	0.48136	3.06951	1.27388
O	0.54052	3.24333	0.0568
O	0.12623	1.84181	1.81395
C	0.77242	4.06905	2.33407
H	-0.13124	4.24196	2.92874
H	1.54541	3.69167	3.01759
H	1.09461	5.00014	1.88561
Ag	0.0093	1.28846	-1.46011
H	-0.01502	1.30559	1.19552

- Transition state – Step 2 - Proposal 4 (5-membered ring pathway)

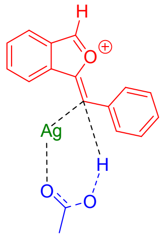

Symbolic Z-matrix:
Charge = 1 Multiplicity = 1

C	4.02928	0.61295	0.27952
C	2.63878	0.51264	0.23966
C	2.07304	-0.77523	0.18572
C	2.92893	-1.92133	0.17674
C	4.34198	-1.81127	0.21429
C	4.87872	-0.53423	0.26645
H	4.48636	1.59597	0.3219
H	2.02171	1.40571	0.24753
H	4.96676	-2.69707	0.2052
H	5.95276	-0.39329	0.29849
C	2.08877	-3.04777	0.12154
H	2.32527	-4.10329	0.08043
O	0.80536	-2.69372	0.11222
C	0.69736	-1.22955	0.15091
C	-1.81466	-1.21463	0.21802
C	-2.93739	-0.4396	-0.17136
C	-2.04641	-2.53617	0.68763
C	-4.23155	-0.97345	-0.15126
H	-2.79179	0.59074	-0.49108
C	-3.34317	-3.05877	0.733
H	-1.21644	-3.14312	1.02677

C	-4.43828	-2.2865	0.30288
H	-5.07292	-0.36709	-0.46908
H	-3.50402	-4.06658	1.10144
H	-5.44122	-2.69967	0.33574
C	-0.49448	-0.56848	0.16031
Ag	-0.32102	1.23203	-1.43576
H	-0.58426	0.8626	0.97985
O	-0.67551	1.65048	1.70643
C	-0.59362	2.90045	1.23691
O	-0.46862	3.14712	0.00504
C	-0.66143	3.97112	2.28245
H	-1.60714	3.89094	2.82695
H	0.14909	3.83116	3.00386
H	-0.58159	4.95566	1.82259

- Transition state – Step 2 - Proposal 4 (6-membered ring pathway)

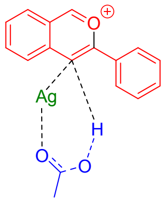

Symbolic Z-matrix:
Charge = 1 Multiplicity = 1

C	4.21827	0.06684	-0.17525
C	2.86454	0.36021	-0.13176
C	1.89834	-0.68156	-0.21191
C	2.38731	-2.03191	-0.33235
C	3.78865	-2.32055	-0.3744
C	4.68813	-1.27925	-0.29867
H	4.94451	0.87058	-0.11335
H	2.52152	1.38659	-0.02897
H	4.11711	-3.35122	-0.46266
H	5.75466	-1.46966	-0.32912
C	1.44217	-3.0533	-0.39639
H	1.67704	-4.10838	-0.48393
O	0.14198	-2.78956	-0.35057
C	0.48099	-0.39647	-0.17431
C	-0.3707	-1.48033	-0.24158
C	-1.84643	-1.50005	-0.22696
C	-2.55007	-2.7188	-0.12092
C	-2.57364	-0.29444	-0.32629
C	-3.95124	-2.72771	-0.10584
H	-2.00778	-3.65415	-0.04756
C	-3.97319	-0.30795	-0.31184
H	-2.05694	0.65463	-0.4283
C	-4.66871	-1.52428	-0.19978
H	-4.48007	-3.67112	-0.02105
H	-4.51895	0.62607	-0.39252
H	-5.75362	-1.53284	-0.18846
C	-0.32018	2.97724	-1.20547
O	-0.41077	3.1708	0.03836
O	-0.04646	1.77278	-1.71968
C	-0.52528	4.06182	-2.21889

H	0.31844	4.08569	-2.91373
H	-1.42821	3.8453	-2.79928
H	-0.6328	5.02727	-1.72509
Ag	-0.04202	1.28052	1.47908
H	0.1084	0.9775	-1.01185

- Molecular complex (products) - Step 2 - Proposal 4 (5-membered ring pathway)

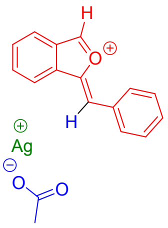

Symbolic Z-matrix:

Charge = 1 Multiplicity = 1

C	4.04566	0.89947	0.49441
C	2.69146	0.58413	0.62493
C	2.30848	-0.72981	0.30926
C	3.27608	-1.68875	-0.12936
C	4.64781	-1.35527	-0.25824
C	5.0147	-0.05386	0.05921
H	4.37943	1.90398	0.73267
H	1.96184	1.31838	0.95912
H	5.37274	-2.09144	-0.59039
H	6.05056	0.25225	-0.0174
C	2.57773	-2.88424	-0.36116
H	2.91376	-3.85795	-0.69321
O	1.26764	-2.73507	-0.10539
C	1.02483	-1.39014	0.31976
C	-1.50911	-1.55584	0.61656
C	-2.62136	-0.74392	0.95995
C	-1.72187	-2.91334	0.26446
C	-3.91695	-1.26938	0.94688
H	-2.45309	0.29317	1.2407
C	-3.01897	-3.4339	0.25545
H	-0.88626	-3.55096	0.00145
C	-4.11672	-2.61632	0.59501
H	-4.75998	-0.64265	1.20997
H	-3.1833	-4.47293	-0.01557
H	-5.11823	-3.03347	0.58403
C	-0.20656	-0.91354	0.6424
Ag	-1.0899	1.17606	-1.37807
H	-0.1841	0.14145	0.94054
O	-0.14409	2.02076	1.47163
C	-0.45798	3.1075	0.87146
O	-0.88536	3.14562	-0.36111
C	-0.33257	4.42367	1.61299
H	-0.94994	4.39607	2.51655
H	0.70491	4.55999	1.93224
H	-0.63667	5.26001	0.98359

- Molecular complex (products) - Step 2 - Proposal 4 (6-membered ring pathway)

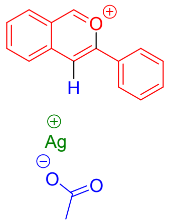

Symbolic Z-matrix:

Charge = 1 Multiplicity = 1

C	-4.08396	0.68226	0.88595
C	-2.6998	0.67244	0.96086
C	-1.99339	-0.51161	0.62798
C	-2.739	-1.66939	0.20758
C	-4.16848	-1.63874	0.14336
C	-4.82421	-0.47401	0.4801
H	-4.62822	1.58577	1.14081
H	-2.1415	1.55212	1.26294
H	-4.70866	-2.52504	-0.17204
H	-5.90581	-0.42113	0.43643
C	-2.0186	-2.80578	-0.14092
H	-2.45484	-3.74181	-0.47127
O	-0.68416	-2.81613	-0.10676
C	-0.56854	-0.5779	0.68054
C	0.08187	-1.7253	0.28389
C	1.52563	-1.9669	0.20779
C	2.02462	-3.25234	-0.09369
C	2.42041	-0.8985	0.43125
C	3.40683	-3.46437	-0.16561
H	1.3419	-4.07595	-0.26683
C	3.79989	-1.1208	0.35531
H	2.04656	0.09534	0.66645
C	4.29831	-2.40135	0.05881
H	3.78827	-4.45336	-0.39359
H	4.48498	-0.29739	0.52735
H	5.36881	-2.56893	0.002
C	1.4168	2.84407	0.92606
O	1.20488	3.017	-0.35137
O	0.94927	1.85054	1.58106
C	2.27012	3.87595	1.63842
H	1.67678	4.3609	2.42033
H	3.11248	3.3762	2.12717
H	2.64235	4.62939	0.9427
Ag	-0.11177	1.53973	-1.34335
H	-0.02073	0.29859	1.00556

- Molecular complex (reagents) - Step 3 - Proposal 4 (5-membered ring pathway)

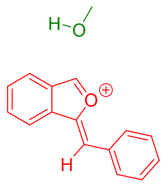

Symbolic Z-matrix:
Charge = 1 Multiplicity = 1

C	3.59639	-2.03435	0.39901
C	2.20728	-1.98482	0.44375
C	1.58091	-0.86639	-0.12737
C	2.37343	0.15793	-0.77035
C	3.7957	0.10924	-0.77514
C	4.39461	-0.99051	-0.17896
H	4.11423	-2.89093	0.81713
H	1.63999	-2.78099	0.90829
H	4.37423	0.90662	-1.22687
H	5.47355	-1.08225	-0.1636
H	1.644	2.10629	-1.67697
O	0.23837	0.77375	-0.97342
C	0.20383	-0.46294	-0.2544
C	-0.96246	-1.04855	0.13789
C	-2.32854	-0.59893	0.00864
C	-3.33488	-1.51393	0.40845
C	-2.69999	0.68357	-0.45603
C	-4.68827	-1.16633	0.31933
H	-3.04651	-2.49732	0.76966
C	-4.05488	1.02742	-0.53001
H	-1.94875	1.41433	-0.74351
C	-5.04725	0.10717	-0.14605
H	-5.44907	-1.87899	0.61901
H	-4.33997	2.01226	-0.88481
H	-6.0953	0.38174	-0.21698
H	-0.8184	-2.01277	0.6326
C	1.47601	1.02879	-1.35193
O	1.6136	2.95388	0.90567
H	2.52622	3.17466	1.22688
C	0.9461	2.13652	1.93998
H	-0.071	1.92068	1.58108
H	1.44295	1.16077	2.12904
H	0.86013	2.68147	2.95027

- Molecular complex (reagents) - Step 3 - Proposal 4 (6-membered ring pathway)

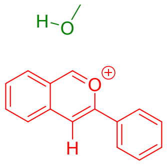

Symbolic Z-matrix:
Charge = 1 Multiplicity = 1

C	-3.87746	-1.75075	0.19134
C	-2.536	-1.96926	0.46299
C	-1.5738	-1.00935	0.04496
C	-2.02293	0.14692	-0.66957
C	-3.41668	0.37111	-0.9006
C	-4.32672	-0.57758	-0.48144
H	-4.61217	-2.49299	0.50249
H	-2.21042	-2.85991	0.99153
H	-3.72833	1.27592	-1.41948
H	-5.38923	-0.43818	-0.66634
C	-1.05758	1.02321	-1.1538
H	-1.27635	1.98218	-1.67309
O	0.22311	0.82106	-0.93991
C	-0.17689	-1.16331	0.28483
C	0.72506	-0.25869	-0.21322
C	2.18712	-0.26425	-0.1265
C	2.92768	0.90562	-0.38662
C	2.86543	-1.4531	0.22244
C	4.32055	0.89332	-0.30105
H	2.41478	1.8286	-0.6548
C	4.2625	-1.46446	0.30987
H	2.31261	-2.36883	0.40542
C	4.99858	-0.28888	0.04949
H	4.88293	1.79831	-0.50532
H	4.77678	-2.37706	0.57103
H	6.08154	-0.30164	0.11476
H	0.17881	-2.00741	0.86048
O	-1.20855	2.53837	0.71613
H	-0.39388	3.09745	0.84921
C	-1.61824	1.96134	1.9796
H	-2.4238	1.25209	1.76705
H	-0.79839	1.42578	2.47421
H	-2.0027	2.73616	2.67506

- Transition state – Step 3 - Proposal 4 (5-membered ring pathway)

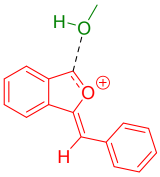

Symbolic Z-matrix:
Charge = 1 Multiplicity = 1

C	3.71078	-1.98126	0.29787
C	2.30885	-1.94092	0.33015
C	1.68477	-0.76216	-0.09284
C	2.46161	0.33979	-0.52912
C	3.8624	0.29959	-0.57217
C	4.48174	-0.88066	-0.14812
H	4.22174	-2.8831	0.61819
H	1.73346	-2.79616	0.66571
H	4.43848	1.1517	-0.91505
H	5.5627	-0.95959	-0.16025
H	1.70251	2.19531	-1.59869
O	0.26244	0.95763	-0.71209
C	0.2758	-0.39314	-0.21059
C	-0.84963	-1.07601	0.07223
C	-2.24772	-0.6512	-0.03392
C	-3.24031	-1.60465	0.29289
C	-2.65179	0.64731	-0.42937
C	-4.59988	-1.27836	0.22251
H	-2.93699	-2.60171	0.60076
C	-4.01349	0.9692	-0.49726
H	-1.91071	1.39569	-0.68154
C	-4.99143	0.01192	-0.1738
H	-5.34867	-2.02165	0.47507
H	-4.31367	1.96676	-0.80114
H	-6.04423	0.26929	-0.22851
H	-0.6837	-2.08821	0.43832
C	1.53082	1.38429	-0.9021

O	1.07343	2.51577	0.50766
H	1.67209	3.27787	0.69077
C	0.58271	1.91053	1.76465
H	-0.42485	1.54365	1.58126
H	1.2536	1.10585	2.07759
H	0.55986	2.69156	2.52688

- Transition state – Step 3 - Proposal 4 (6-membered ring pathway)

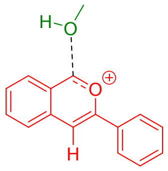

Symbolic Z-matrix:

Charge = 1 Multiplicity = 1

C	-3.89334	-1.76806	0.15244
C	-2.53896	-1.96874	0.42342
C	-1.59625	-0.98584	0.0531
C	-2.05798	0.19442	-0.58124
C	-3.42557	0.39479	-0.85598
C	-4.34229	-0.58983	-0.49103
H	-4.615	-2.52623	0.43739
H	-2.20041	-2.87611	0.91275
H	-3.75043	1.3101	-1.34154
H	-5.39832	-0.45513	-0.69511
C	-1.0777	1.21587	-0.89586
H	-1.30979	1.99646	-1.61289
O	0.23534	0.87918	-0.9117
C	-0.17348	-1.1418	0.29267
C	0.71661	-0.24291	-0.19565
C	2.18512	-0.26407	-0.12263
C	2.9283	0.90204	-0.39803
C	2.86154	-1.45181	0.2281
C	4.32748	0.88114	-0.31363
H	2.41717	1.81785	-0.67264
C	4.25859	-1.46549	0.31484
H	2.30728	-2.36507	0.4189
C	4.99715	-0.29969	0.04528
H	4.8916	1.78335	-0.52534
H	4.77098	-2.38338	0.58287
H	6.08012	-0.31431	0.11002
H	0.17468	-2.00164	0.85163
O	-1.17379	2.3406	0.54451
H	-0.48763	3.04726	0.53536
C	-1.56333	1.89431	1.89758
H	-2.41629	1.22718	1.77523
H	-0.72777	1.37949	2.37527
H	-1.86397	2.77589	2.46535

- Molecular complex (products) - Step 3 - Proposal 4 (5-membered ring pathway)

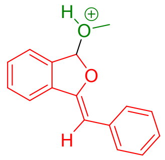

Symbolic Z-matrix:

Charge = 1 Multiplicity = 1

C	3.61429	-2.07015	0.30951
C	2.20728	-1.98482	0.35425
C	1.61671	-0.79479	-0.09157
C	2.40923	0.28323	-0.51975
C	3.7957	0.19874	-0.59614
C	4.39461	-1.00841	-0.17896
H	4.09633	-2.98043	0.65603
H	1.60419	-2.81679	0.71139
H	4.39213	1.03192	-0.95837
H	5.47355	-1.11805	-0.2173
H	1.6619	2.14209	-1.65907
O	0.20257	0.93485	-0.77652
C	0.20383	-0.40924	-0.2007
C	-0.92666	-1.04855	0.11999
C	-2.32854	-0.59893	0.00864
C	-3.33488	-1.51393	0.39055
C	-2.71789	0.68357	-0.45603
C	-4.68827	-1.16633	0.31933
H	-3.04651	-2.49732	0.75176
C	-4.07278	1.02742	-0.53001
H	-1.96665	1.39643	-0.76141
C	-5.06515	0.10717	-0.14605
H	-5.44907	-1.87899	0.61901
H	-4.35787	2.01226	-0.88481
H	-6.1132	0.38174	-0.19908
H	-0.7826	-2.04857	0.5252
C	1.49391	1.45839	-0.77913
O	1.5778	2.34528	0.33287
H	2.49042	2.74506	0.38558
C	0.964	2.06492	1.68938
H	-0.0889	1.84908	1.50948
H	1.47875	1.21447	2.16484
H	1.07493	3.00367	2.28797

- Molecular complex (products) - Step 3 - Proposal 4 (6-membered ring pathway)

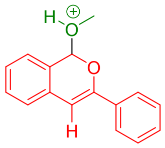

Symbolic Z-matrix:

Charge = 1 Multiplicity = 1

C	-3.90066	-1.78555	0.14494
C	-2.536	-1.96926	0.41659

C	-1.6086	-0.97455	0.06816
C	-2.08093	0.21652	-0.51877
C	-3.42828	0.39431	-0.831
C	-4.34992	-0.61238	-0.49304
H	-4.61217	-2.55099	0.42129
H	-2.18722	-2.88311	0.88713
H	-3.76313	1.31072	-1.30348
H	-5.40083	-0.48458	-0.71274
C	-1.09239	1.34801	-0.713
H	-1.33435	1.98218	-1.59189
O	0.24631	0.91386	-0.89351
C	-0.16529	-1.12851	0.30803
C	0.71346	-0.23549	-0.17842
C	2.18712	-0.26425	-0.1149
C	2.92768	0.90562	-0.38662
C	2.86543	-1.4531	0.22244
C	4.33215	0.88172	-0.30105
H	2.41478	1.817	-0.6548
C	4.2625	-1.46446	0.30987
H	2.31261	-2.36883	0.40542
C	4.99858	-0.30048	0.04949
H	4.89453	1.78671	-0.50532
H	4.77678	-2.38865	0.57103
H	6.08154	-0.31324	0.11476
H	0.17881	-1.99582	0.86048
O	-1.16215	2.20197	0.37973
H	-0.56788	3.01625	0.25761
C	-1.54864	1.88014	1.8056
H	-2.4354	1.24049	1.75545
H	-0.70559	1.39098	2.28861
H	-1.7939	2.85216	2.25746

- Acetate

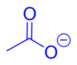

Symbolic Z-matrix:
Charge = -1 Multiplicity = 1

C	0.48076	-1.75941	0.80145
C	1.24812	-3.09461	0.80145
H	0.5512	-3.90652	0.79949
H	1.86463	-3.15167	-0.07123
H	1.8617	-3.15335	1.67607
O	1.1078	-0.66836	0.80355
O	-0.94923	-1.75941	0.79904

- Acetic acid

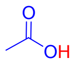

Symbolic Z-matrix:
Charge = 0 Multiplicity = 1

C	0.48076	-1.75941	0.80145
C	1.24812	-3.09461	0.80145
H	0.5512	-3.90652	0.79949
H	1.86463	-3.15167	-0.07123
H	1.8617	-3.15335	1.67607
O	1.1078	-0.66836	0.80355
O	-0.94923	-1.75941	0.79904
H	-1.26969	-0.85447	0.79938

- Product - Proposal 4 (5-membered ring pathway)

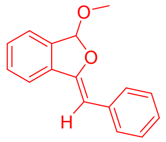

Symbolic Z-matrix:
Charge = 0 Multiplicity = 1

C	3.54828	-2.11692	0.26134
C	2.1486	-2.01122	0.28447
C	1.57918	-0.7857	-0.08033
C	2.39752	0.29558	-0.45199
C	3.78836	0.20199	-0.48048
C	4.36182	-1.02787	-0.11659
H	4.01566	-3.05671	0.53537
H	1.53079	-2.85555	0.57024
H	4.40571	1.04574	-0.77064
H	5.43971	-1.14434	-0.12757
H	1.6909	2.07329	-1.62981
O	0.18982	0.98431	-0.67558
C	0.17724	-0.36396	-0.17015
C	-0.95962	-1.01783	0.12329
C	-2.35083	-0.55359	0.04089
C	-3.36171	-1.45639	0.44499
C	-2.73174	0.73234	-0.41186
C	-4.71316	-1.09163	0.40193
H	-3.07919	-2.44634	0.79365
C	-4.0857	1.09351	-0.45386
H	-1.97699	1.44082	-0.72909
C	-5.08131	0.18773	-0.0485
H	-5.47403	-1.79828	0.71694
H	-4.36531	2.08244	-0.80311
H	-6.12734	0.47446	-0.08362
H	-0.81817	-2.03692	0.47946
C	1.51056	1.46236	-0.74746
O	1.60892	2.47623	0.35362
C	1.2666	2.08796	1.75769
H	0.25541	1.68713	1.7219
H	1.99645	1.3582	2.10859
H	1.29827	3.00737	2.34004

- Product - Proposal 4 (6-membered ring pathway)

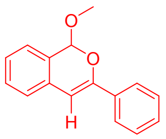

Symbolic Z-matrix:

Charge = 0 Multiplicity = 1

C	-1.16304	-4.43605	-0.10162
C	0.0817	-3.81003	0.03088
C	0.18318	-2.40534	-0.05143
C	-0.9881	-1.64522	-0.25478
C	-2.23326	-2.27276	-0.39491
C	-2.32512	-3.67004	-0.3193
H	-1.23314	-5.51724	-0.03976
H	0.97959	-4.39921	0.19304
H	-3.12462	-1.67208	-0.55621
H	-3.28717	-4.1596	-0.42666
C	-0.90584	-0.13768	-0.27435
H	-1.56958	0.31076	-1.01433
O	0.42008	0.35323	-0.5703
C	1.46488	-1.71247	0.05612
C	1.55456	-0.38766	-0.20652
C	2.77749	0.43988	-0.18657
C	2.67485	1.84565	-0.16473
C	4.05657	-0.15739	-0.18298
C	3.83073	2.63995	-0.12777
H	1.69386	2.30675	-0.17457
C	5.20777	0.63867	-0.14358
H	4.15722	-1.23733	-0.22387
C	5.1005	2.0411	-0.11474
H	3.73951	3.72115	-0.10851
H	6.18595	0.16899	-0.14335
H	5.99463	2.65542	-0.08741
H	2.34689	-2.27718	0.33321
O	-1.35727	0.44286	0.97777
C	-0.6155	0.10096	2.19007
H	-1.15283	0.56354	3.01884
H	-0.59188	-0.984	2.32916
H	0.39976	0.5051	2.13768

- Molecular complex (reagents) - Proposal 5 (5-membered ring pathway)

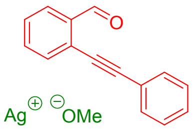

Symbolic Z-matrix:

Charge = 0 Multiplicity = 1

C	-3.72711	-2.01384	-0.6624
C	-2.34621	-1.85175	-0.57826
C	-1.7427	-0.57176	-0.42235
C	-2.54435	0.62837	-0.37654
C	-3.94516	0.41079	-0.47162
C	-4.54433	-0.8587	-0.59356
H	-4.15903	-3.00777	-0.77065
H	-1.68966	-2.72891	-0.63152
H	-4.58466	1.29276	-0.44145
H	-5.62857	-0.95552	-0.6449
C	-2.15735	2.14271	-0.28976
H	-3.13422	2.66927	-0.69485
O	-0.98299	2.69603	-0.74242
C	-0.28464	-0.65202	-0.30777
C	0.89807	0.05777	-0.32034
C	2.1954	0.63586	-0.33578
C	3.42351	-0.04358	-0.25401
C	2.11898	2.0467	-0.45701
C	4.60908	0.70468	-0.28251
H	3.46654	-1.12711	-0.16373
C	3.32017	2.77395	-0.4842
H	1.12198	2.48703	-0.51393
C	4.55586	2.10364	-0.40315
H	5.56765	0.2001	-0.21908
H	3.29227	3.85516	-0.57698
H	5.48449	2.68105	-0.43335
Ag	0.99306	-2.41339	0.51188
O	-2.42676	2.371	1.24801
C	-2.07181	3.72905	1.58829
H	-2.67126	4.46045	1.00944
H	-2.2894	3.86778	2.65611
H	-1.01673	3.92747	1.38768

- Transition state - Proposal 5 (5-membered ring pathway)

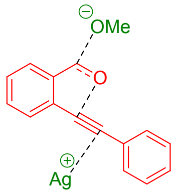

Symbolic Z-matrix:

Charge = 0 Multiplicity = 1

C	-4.10388	-0.81563	0.04766
C	-2.7025	-0.81794	0.04286
C	-2.02102	0.4092	-0.02816
C	-2.77766	1.60629	-0.09327
C	-4.182	1.61166	-0.09044
C	-4.84642	0.38517	-0.01758
H	-4.63135	-1.76198	0.10298
H	-2.15756	-1.75599	0.0944
H	-4.7259	2.54869	-0.14337
H	-5.92962	0.34866	-0.01143
C	-1.89659	2.74417	-0.1727
H	-2.22881	3.77897	-0.25023
O	-0.59034	2.44588	-0.15437
C	-0.58343	0.60382	-0.04368
C	0.60102	0.08332	-0.02447
C	1.96936	0.6105	-0.01705
C	3.03398	-0.17583	-0.51254
C	2.25722	1.89787	0.4989
C	4.34569	0.3168	-0.52162
H	2.82716	-1.17157	-0.89898
C	3.57297	2.37878	0.50869
H	1.45267	2.50705	0.8936
C	4.62029	1.59535	-0.00769
H	5.14943	-0.29603	-0.91592
H	3.78195	3.3631	0.91511
H	5.63739	1.97349	-0.002

Ag	0.47515	-2.2089	0.08605
O	-1.80092	2.78569	1.62427
C	-0.90042	3.8269	2.06103
H	-1.2351	4.81583	1.70887
H	-0.90148	3.81926	3.15485
H	0.11759	3.65549	1.69213

- Molecular complex (products) - Proposal 5 (5-membered ring pathway)

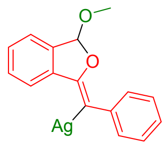

Symbolic Z-matrix:

Charge = 0 Multiplicity = 1

C	-3.46711	-1.97385	-0.6424
C	-2.10621	-1.59175	-0.59826
C	-1.7827	-0.21176	-0.48235
C	-2.84435	0.70837	-0.39654
C	-4.18516	0.35079	-0.43162
C	-4.50433	-1.0187	-0.57356
H	-3.71903	-3.02777	-0.75065
H	-1.32966	-2.34891	-0.67152
H	-4.96466	1.11276	-0.36145
H	-5.54857	-1.33552	-0.6249
C	-2.21735	2.06271	-0.20976
H	-2.65422	2.88927	-0.87485
O	-0.80299	1.81603	-0.50242
C	-0.40464	0.40798	-0.44777
C	0.89807	-0.30224	-0.28034
C	2.1354	0.53586	-0.31578
C	3.40351	-0.08358	-0.23401
C	2.09898	1.9467	-0.43701
C	4.58908	0.66468	-0.28251
H	3.44654	-1.16711	-0.14373
C	3.28017	2.69395	-0.4842
H	1.12198	2.42703	-0.49393
C	4.53586	2.06364	-0.40315
H	5.54765	0.1601	-0.21908
H	3.23227	3.77516	-0.57698
H	5.44449	2.64105	-0.43335
Ag	0.99306	-2.37339	0.49188
O	-2.46676	2.431	1.16801
C	-1.97181	3.74905	1.50829
H	-2.45126	4.52045	0.88944
H	-2.2094	3.92778	2.55611
H	-0.87673	3.80747	1.36768

- Molecular complex (reagents) - Proposal 5 (6-membered ring pathway)

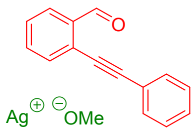

Symbolic Z-matrix:

Charge = 0 Multiplicity = 1

C	-4.16062	-0.92671	-0.69007
C	-2.78197	-1.17332	-0.56008
C	-1.89958	-0.09198	-0.3833
C	-2.35185	1.25929	-0.32284
C	-3.73532	1.45807	-0.46508
C	-4.63548	0.39211	-0.6491
H	-4.85261	-1.75204	-0.83039
H	-2.40624	-2.18817	-0.61483
H	-4.10648	2.47722	-0.42885
H	-5.69374	0.59982	-0.75457
C	-1.44551	2.51051	-0.13185
H	-2.21807	3.40052	-0.10663
O	-0.35572	2.69072	-0.8624
O	-1.21188	2.38653	1.42877
C	-0.33137	3.43775	1.85552
H	-0.69606	4.43667	1.53337
H	-0.28244	3.4201	2.94934
H	0.68663	3.31633	1.43662
C	-0.49127	-0.17334	-0.33121
C	0.88219	-0.34237	-0.25758
C	2.19813	0.24302	-0.35389
C	3.21035	-0.72028	-0.28483
C	2.49292	1.63421	-0.50472
C	4.55213	-0.3431	-0.34492
H	2.93136	-1.77017	-0.18684
C	3.8588	1.99857	-0.55473
H	1.63408	2.32769	-0.57502
C	4.88318	1.0125	-0.47857
H	5.33572	-1.0981	-0.29187
H	4.14295	3.04322	-0.65502
H	5.92707	1.31475	-0.50851
Ag	0.22596	-2.54011	0.44927

- Transition state - Proposal 5 (6-membered ring pathway)

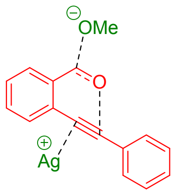

Symbolic Z-matrix:

Charge = 0 Multiplicity = 1

C	3.84786	1.8767	-0.60479
C	2.45069	1.83534	-0.66875
C	1.75592	0.62514	-0.45904
C	2.47288	-0.55912	-0.16749
C	3.87603	-0.50288	-0.1131
C	4.56613	0.69855	-0.32892
H	4.37392	2.81123	-0.77044
H	1.87997	2.73324	-0.88794
H	4.43023	-1.41297	0.10644
H	5.65019	0.71929	-0.28067
C	1.62993	-1.81672	0.10046

H	2.17763	-2.69034	-0.29412
O	0.32311	-1.88833	-0.26925
O	2.45847	-1.48175	1.67444
C	2.4731	-2.87347	2.11879
H	3.39303	-3.36389	1.78052
H	2.44505	-2.86568	3.21011
H	1.60153	-3.41282	1.72942
C	0.30109	0.57776	-0.56635
C	-0.59188	-0.35149	-0.6229
C	-1.95423	-0.83724	-0.72199
C	-2.95974	0.07994	-1.11242
C	-2.30501	-2.16978	-0.41714
C	-4.29675	-0.33147	-1.17658
H	-2.68917	1.10015	-1.36761
C	-3.64599	-2.5717	-0.49274
H	-1.51773	-2.8607	-0.14021
C	-4.64571	-1.65806	-0.86689
H	-5.06244	0.37784	-1.4733
H	-3.91014	-3.59832	-0.25999
H	-5.68223	-1.97464	-0.92128
Ag	-0.51848	2.67742	-0.97111

- Molecular complex (products) - Proposal 5 (6-membered ring pathway)

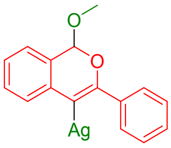

Symbolic Z-matrix:
Charge = 0 Multiplicity = 1

C	-4.16062	-0.90671	-0.69007
C	-2.80197	-1.19332	-0.54008
C	-1.83958	-0.17198	-0.3633
C	-2.29185	1.15929	-0.32284
C	-3.65532	1.45807	-0.46508
C	-4.59548	0.43211	-0.6491
H	-4.87261	-1.71204	-0.83039
H	-2.44624	-2.22817	-0.55483
H	-3.98648	2.49722	-0.42885
H	-5.65374	0.65982	-0.75457
C	-1.28551	2.2705	-0.07185
H	-1.65807	3.24052	-0.54663
O	0.00428	2.01072	-0.6624
O	-1.23188	2.42653	1.36877
C	-0.31137	3.45775	1.81552
H	-0.61606	4.43667	1.43337
H	-0.36244	3.4601	2.90934
H	0.70663	3.23633	1.49662
C	-0.39127	-0.59334	-0.19121
C	0.50219	0.61763	-0.41758
C	2.01812	0.60302	-0.41389
C	2.89035	-0.54028	-0.32483
C	2.63292	1.87421	-0.52472
C	4.29213	-0.3631	-0.34492
H	2.49136	-1.55017	-0.24684
C	4.0188	2.03857	-0.55473
H	1.97408	2.72769	-0.59502
C	4.86318	0.9325	-0.45857
H	4.93572	-1.2381	-0.27187
H	4.42295	3.04322	-0.65502
H	5.94707	1.05475	-0.48851
Ag	0.20596	-2.54011	0.44927

17. REFERENCES

1 T. Hao, L. Huang, Y. Wei and M. Shi, *Org. Lett.*, 2021, **23**, 5133–5137.

2 M. Dell'Acqua, B. Castano, C. Cecchini, T. Pedrazzini, V. Pirovano, E. Rossi, A. Caselli and G. Abbiati, *J. Org. Chem.*, 2014, **79**, 3494–3505.

3 P. C. Too and S. Chiba, *Chem. Commun.*, 2012, **48**, 7634.

4 C. Kingston, M. D. Palkowitz, Y. Takahira, J. C. Vantourout, B. K. Peters, Y. Kawamata and P. S. Baran, *Acc. Chem. Res.*, 2020, **53**, 72–83.

5 C. Zhang, G. Wang, L. Zhan, X. Yang, J. Wang, Y. Wei, S. Xu, M. Shi and J. Zhang, *ACS Catal.*, 2020, **10**, 6682–6690.

6 S. Liu, H. Qian, T. Zhang, H. Xie, Z. Han, W. Guo, H. Huang and J. Sun, *Angew. Chemie Int. Ed.*, 2021, **60**, 21272–21276.

7 H. Zhan, M. Hou, Y. Li, Z. Chen, Y. Wei and S. Liu, *ChemistrySelect*, 2021, **6**, 11537–11540.

8 N. Nardangeli, N. Topolovčan, R. Simionescu and T. Hudlický, *European J. Org. Chem.*, 2020, **2020**, 227–233.

9 M. Dell'Acqua, D. Facoetti, G. Abbiati and E. Rossi, *Synthesis (Stuttg).*, 2010, **2010**, 2367–2378.

10 M. J. Frisch, G. W. Trucks, H. B. Schlegel, G. E. Scuseria, M. A. Robb, J. R. Cheeseman, G. Scalmani, V. Barone, B. Mennucci, G. A. Petersson, H. Nakatsuji, M. Caricato, X. Li, H. P. Hratchian, A. F. Izmaylov, J. Bloino, G. Zheng, J. L. Sonnenberg, M. Hada, M. Ehara, K. Toyota, R. Fukuda, J. Hasegawa, M. Ishida, T. Nakajima, Y. Honda, O. Kitao, H. Nakai, T. Vreven, J. A. Montgomery, J. J. E. Peralta, F. Ogliaro, M. Bearpark, J. J. Heyd, E. Brothers, K. N. Kudin, V. N. Staroverov, T. Keith, R. Kobayashi, J. Normand, K. Raghavachari, A. Rendell, J. C. Burant, S. S. Iyengar, J. Tomasi, M. Cossi, N. Rega, J. M. Millam, M. Klene, J. E. Knox, J. B. Cross, V. Bakken, C. Adamo, J. Jaramillo, R. Gomperts, R. E. Stratmann, O. Yazyev, A. J. Austin, R. Cammi, C. Pomelli, J. W. Ochterski, R. L. Martin, K. Morokuma, V. G. Zakrzewski, G. A. Voth, P. Salvador, J. J. Dannenberg, S. Dapprich, A. D. Daniels, O. Farkas, J. B. Foresman, J. V. Ortiz, J. Cioslowski and F. D. J., 2013.

11 Y. Zhao and D. G. Truhlar, *Theor. Chem. Acc.*, 2008, **120**, 215–241.

12 S. Maeda, Y. Harabuchi, Y. Ono, T. Taketsugu and K. Morokuma, *Int. J. Quantum Chem.*, 2015, **115**, 258–269.
